# Synergistic inflammatory signaling by cGAS may be involved in the development of atherosclerosis

**DOI:** 10.18632/aging.202491

**Published:** 2021-02-11

**Authors:** Guan-Feng Lu, Sheng-Cai Chen, Yuan-Peng Xia, Zi-Ming Ye, Fei Cao, Bo Hu

**Affiliations:** 1 Department of Neurology, Union Hospital, Tongji Medical College, Huazhong University of Science and Technology, Wuhan 430022, China; 2 Department of Neurology, The First Affiliated Hospital, Guangxi Medical University, Nanning 530021, Guangxi, China

**Keywords:** cGAS, atherosclerosis, RNA-seq, M1 polarization, synergistic inflammatory signaling

## Abstract

Inappropriate activation or overactivation of cyclic GMP-AMP synthase (cGAS) by double-stranded deoxyribonucleic acid (dsDNA) initiates a regulatory signaling cascade triggering a variety of inflammatory responses, which are a great threat to human health. This study focused on identifying the role of cGAS in atherosclerosis and its potential mechanisms. The relationship between cGAS and atherosclerosis was identified in an ApoE ^-/-^ mouse model. Meanwhile, RNA sequencing (RNA-seq) analysis of the underlying mechanisms of atherosclerosis in RAW264.7 macrophages treated with cGAS inhibition was conducted. Results showed that cGAS was positively correlated with atherosclerotic plaque area, and was mainly distributed in macrophages. RNA-seq analysis revealed that inflammatory response, immune response and cytokine–cytokine receptor interaction may play important roles in the development of atherosclerosis. Real-time quantitative polymerase chain reaction (RT-qPCR) results showed that the expression of the pro-inflammatory factors, signal transducer and activator of transcription (*Stat*), interferon regulatory factor (*Irf*), toll-like receptors (*Tlrs*), and type I interferons (*Ifns*) were synergistically reduced when cGAS was inhibited. Furthermore, cGAS inhibition significantly inhibited RAW264.7 macrophage M1 polarization. These results demonstrate that cGAS may contribute to the development of atherosclerosis through synergistic inflammatory signaling of TLRs, STAT/IRF as well as IFNs, leading to macrophage M1 polarization.

## INTRODUCTION

The identification of exogenous deoxyribonucleic acid (DNA) is the basic function of host defense. Intracellular signaling induced by innate DNA sensing can lead to strong anti-infective immune responses [1]. Cyclic GMP–AMP synthase (cGAS) located in the cytoplasm can bind to double-stranded DNA (dsDNA). Activated cGAS can catalyze the synthesis of cyclic dinucleotide GMP–AMP (cGAMP), which mediates the canonical stimulator of interferon response cGAMP interactor (STING), and the noncanonical signaling pathways [2, 3]. Although cGAS was initially identified as a critical part of anti-infective immune defense [4, 5], recent research has revealed that mis-localized self-DNA triggers improper activation or overactivation of cGAS and thus provokes endogenous inflammation, ultimately, leading to disease [3, 6–9].

Atherosclerosis, the primary etiological factor of cardio-cerebrovascular diseases which are the leading cause of death worldwide, is a chronic inflammatory process with characteristic modified low-density lipoprotein (mLDL) deposition which drives the recruitment of circulating immune cells that subsequently trigger inflammatory cascades [10]. Great efforts have been made to elucidate the mechanisms of atherosclerosis, but nevertheless, it has not yet been fully clarified. Accumulating evidence has shown that mis-localized self-DNA and DNA damage occur in atherosclerosis. The Health 2000 Survey showed that in women without hormone replacement therapy (HRT), higher circulating cell-free DNA levels indicate worse arterial elasticity [11]. Mitochondrial DNA(mtDNA), a small double-stranded circular form of DNA, is increasingly recognized as a potent stimulus in innate immune responses and inflammation [12]. A study carried out by Zhang et al. demonstrated that human atherosclerotic plasma contains higher concentrations of mtDNA than those in healthy controls [13]. Furthermore, mtDNA is released into the cytoplasm under oxidized LDL(oxLDL) stimulation [14]. In addition, it is reported that increased plaque necrosis core and induction by mtDNA damage are attributable to apoptosis of smooth muscle cells and monocytes rather than reactive oxygen species [15]. Furthermore, mtDNA can directly provoke inflammatory response by engaging the cGAS-STING pathway. Besides, extracellular release of oxidized mtDNA is also demonstrated to be pro-inflammatory *in vitro*, and type-I interferon (IFN) signaling was stimulated in response to the DNA sensor when oxidized mtDNA is injected into mice [16]. Hypothetically therefore, cGAS may play a pivotal role in the development of atherosclerosis. Exploring the role of cGAS in atherosclerosis and the possible underlying mechanisms may provide a new potential therapeutic target for atherosclerosis.

Bioinformatics technology is increasingly applied to unearth the potential targets of diseases, thereby enabling researchers to identify the underlying mechanisms. Macrophages are critical in immune inflammation and play a central role in the development of atherosclerosis, and a study carried out by Bai et al. found that the cGAS-cGAMP-STING pathway is activated in macrophages from high-fat diet (HFD)-induced obese mice [17]. These imply that cGAS may regulate atherosclerosis through macrophages.

This study focused on exploring the relationship between cGAS and atherosclerosis using *in vivo* and *in vitro* atherosclerosis model experiments and identifying the underlying differentially-expressed genes (DEGs) and signaling of cGAS inhibition in macrophages using RNA-sequencing (RNA-seq) analysis, which were associated with atherosclerosis.

## RESULTS

### Mitochondrial DNA damage in atherosclerosis

**Figure 1:**
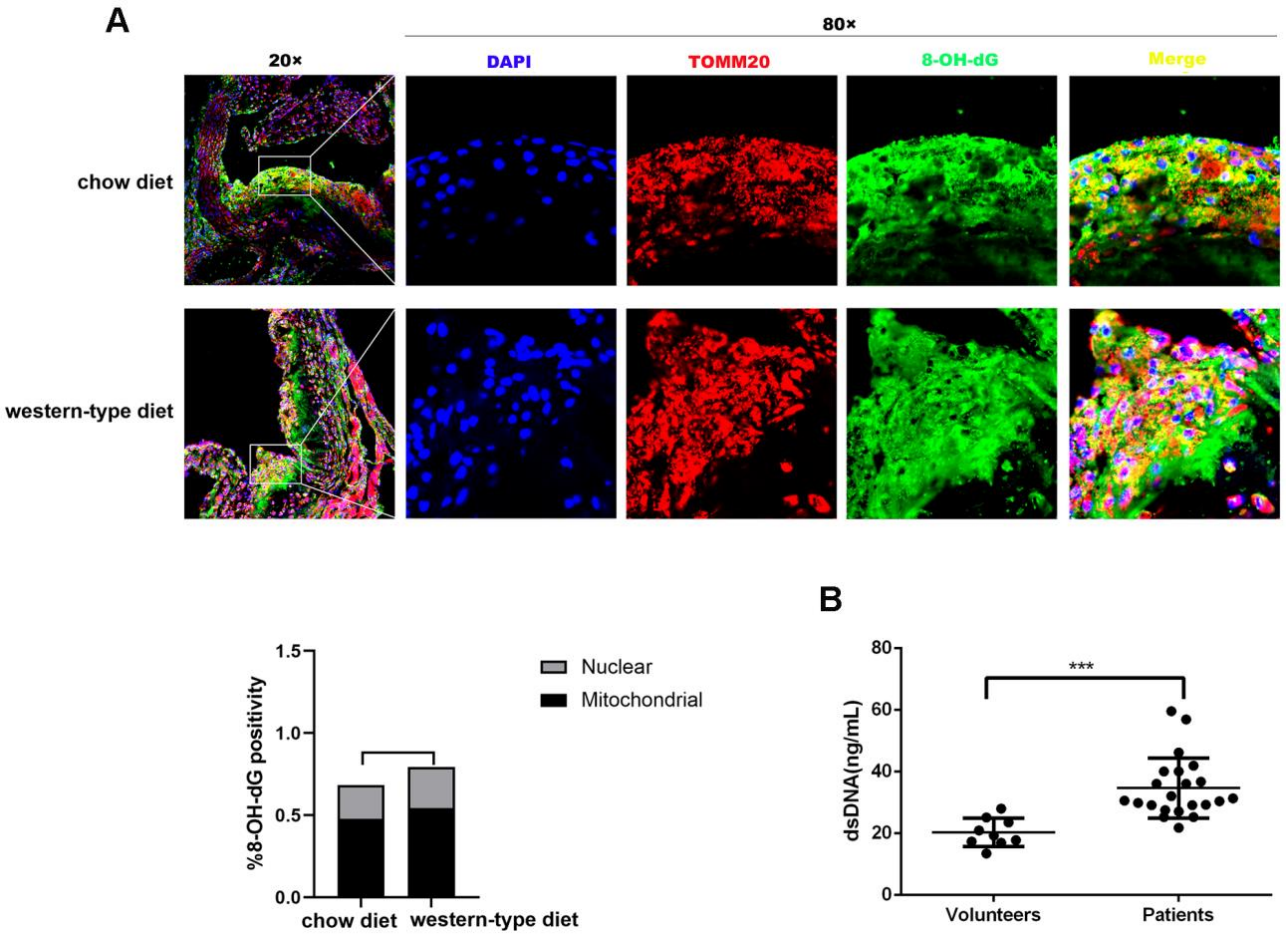
**Mitochondrial DNA damage in atherogenesis.** ApoE ^-/-^ mice were fed a western-type diet or a chow diet for 16 weeks. Immunofluorescence was used to analyze aortic root plaques. (**A**) Oxidative damage to DNA was measured by immunostaining of 8-OH-dG (green) (n = 6, Scale bars: 100μm for 20× images and 10μm for 80× images). Quantitative analysis of 8-OH-dG+ cells in selected areas showed that strong 8-OH-dG staining was discovered mainly in mitochondria (TOMM20, red) compared with nuclei (DAPI, blue) (*P* = 0.037 in the chow diet group, and *P* = 0.018 in the western-type diet group). (**B**) The plasma dsDNA levels of atherosclerosis patients and volunteers were analyzed using a PicoGreen ® dsDNA quantitative kit. ***P< 0.001. Data are means ± SD. 8-OH-dG, 8-Oxo-2′-deoxyguanosine; SD, standard deviation.

It is well known that mtDNA damage correlates with atherosclerosis progression [15, 18, 19]. DNA with oxidative damage is resistant to degradation by cytosolic nuclease three prime repair exonuclease 1(TREX1), and enhances STING-dependent immune sensing [20]. Released oxidized mtDNA acts as a powerful inflammatory stimulus [16]. In accordance with previous findings, immunofluorescence revealed that 8-Oxo-2′-deoxyguanosine (8-OH-dG), a marker for oxidative damage to DNA, can be detected in the plaque of ApoE ^-/-^ mice and strong 8-OH-dG staining was discovered mainly in mitochondria, irrespective of diet. In addition, part of the 8-OH-dG staining overlapped with neither nucleus nor mitochondria (Figure 1A), suggesting that oxidized DNA can be released into the cytoplasm or outside the cell. Additionally, human atherosclerotic plasma contained higher concentrations of dsDNA than those in healthy controls (Figure 1B), consistent with results demonstrated by previous studies [11, 13]. This released DNA could be the actuator for the development of atherosclerosis.

### Differences in cGAS expression identified by database analysis based on the Gene Expression Omnibus

Although cGAS acts mainly as a DNA sensor and the role in broadly eliciting immune responses and inflammation has also been reported, whether cGAS contributes to atherosclerosis is yet to be uncovered. In the present study, the dataset GSE40156 from the Gene Expression Omnibus (GEO) was re-analyzed by bioinformatics methods and it was found that the expression of cGAS was increased in the total aorta of ApoE ^-/-^ mice. Furthermore, cGAS expression in ApoE ^-/-^ mice was significantly increased with age (Table 1). It is well known that macrophages are of great importance in the initiation, progression and regression of atherosclerosis and different subtypes of macrophages affect the outcome of atherosclerosis [21]. To explore the difference of cGAS in macrophage polarization, GSE57614 from the GEO was assayed and it was found that cGAS expression remarkably increased in M1 macrophages when compared with M2 macrophages (Table 2). Collectively, the results of these bioinformatics analyses results mentioned above imply that cGAS may play a pivotal role in atherosclerosis.

**Table 1:** cGAS expression in aorta.

**Comparison**	**log2FC**	***P* value**
ApoE-Aorta-6-weeks vs wt-Aorta-6-weeks	1.204	0.947
ApoE-Aorta-32-weeks vs wt-Aorta-32-weeks	0.294	0.599
ApoE-Aorta-78-weeks vs wt-Aorta-78-weeks	1.373	0.067
ApoE-Aorta-32-weeks vs ApoE-Aorta-6-weeks	1.117	0.073
ApoE-Aorta-78-weeks vs ApoE-Aorta-6-weeks	2.671	0.000

# 0.000 means < 0.0001.

**Table 2:** cGAS expression in macrophage polarization.

**Comparison**	**log2FC**	***P* value**
M2a macrophage 6h vs M1 macrophage 6h	-3.200	0.000
M2c macrophage 6h vs M1 macrophage 6h	-2.560	0.001
M2a macrophage 12h vs M1 macrophage 12h	-2.610	0.000
M2c macrophage 12h vs M1 macrophage 12h	-2.250	0.001
M2a macrophage 24h vs M1 macrophage 24h	-2.70	0.001
M2c macrophage 24h vs M1 macrophage 24h	-2.420	0.002

# Human macrophage M1 polarization was obtained by Lipopolysaccharide (LPS) and interferon gamma (IFNγ), M2a by IL4, whereas IL10 induced a “deactivated” state (M2c). 0.000 means < 0.0001.

### cGAS as a novel factor for modulating atherosclerosis

**Figure 2:**
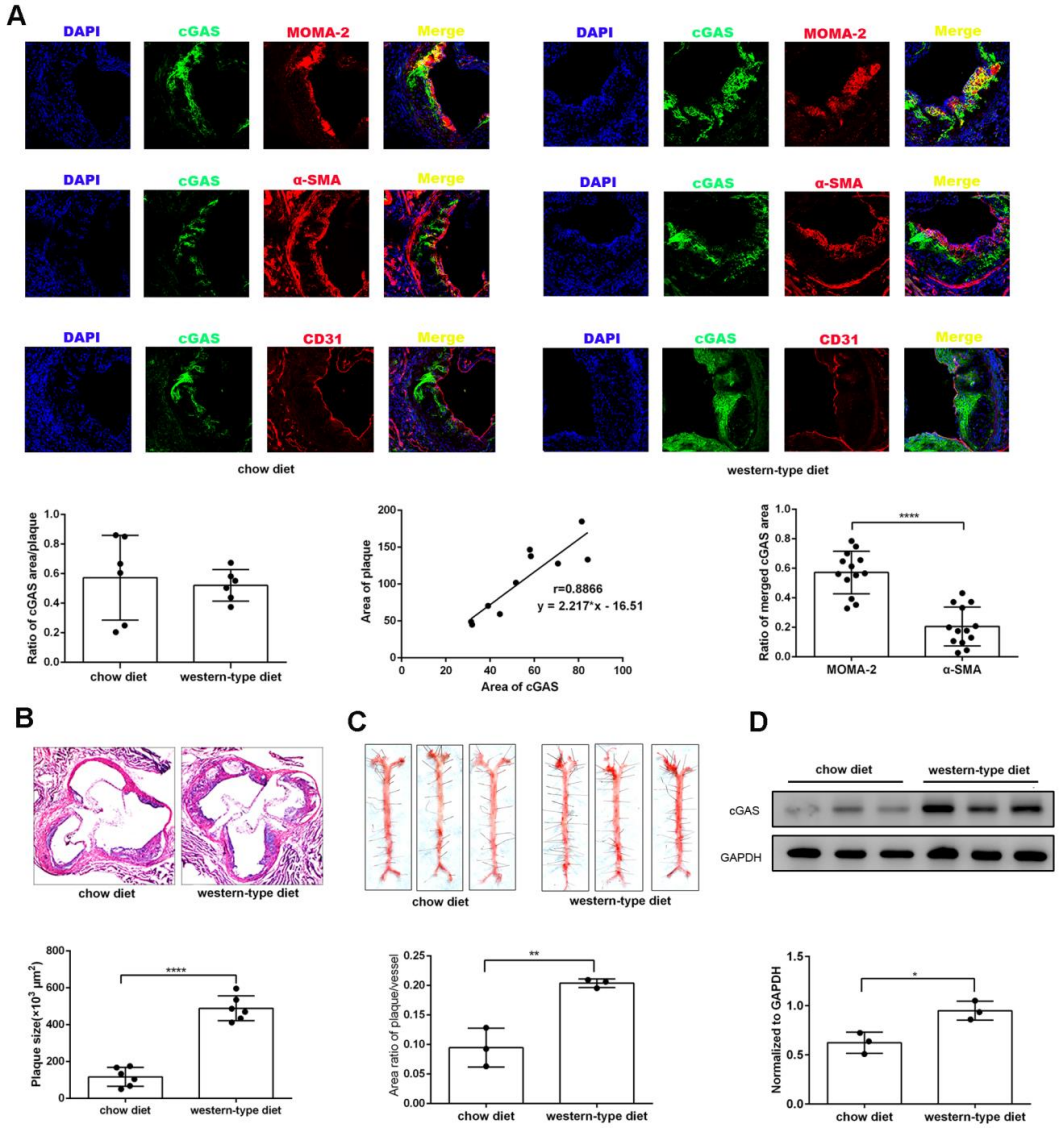
**cGAS was positively correlated with atherosclerosis.** ApoE ^-/-^ mice were fed either a western-type diet or a chow diet for 16 weeks. (**A**) Immunostaining of cGAS (green) and the macrophage marker MOMA-2 (red), the smooth muscle cell marker α-SMA (red), the endothelial cell marker CD31 (red) and their co-localization in atherosclerotic plaques. (n = 6, Scale bar: 100 μm). (**B**) Histological analysis of the aortic root stained with hematoxylin and eosin. (**C**) Oil Red-O analysis of lesion area as a percentage of in total aortic area in ApoE ^-/-^ mice. (**D**) The protein expression of cGAS in aorta was detected by western blotting. * *P* < 0.05, ** *P* < 0.01, *** *P* < 0.001, **** *P* < 0.0001. Data are means ± SD.

**Figure 3:**
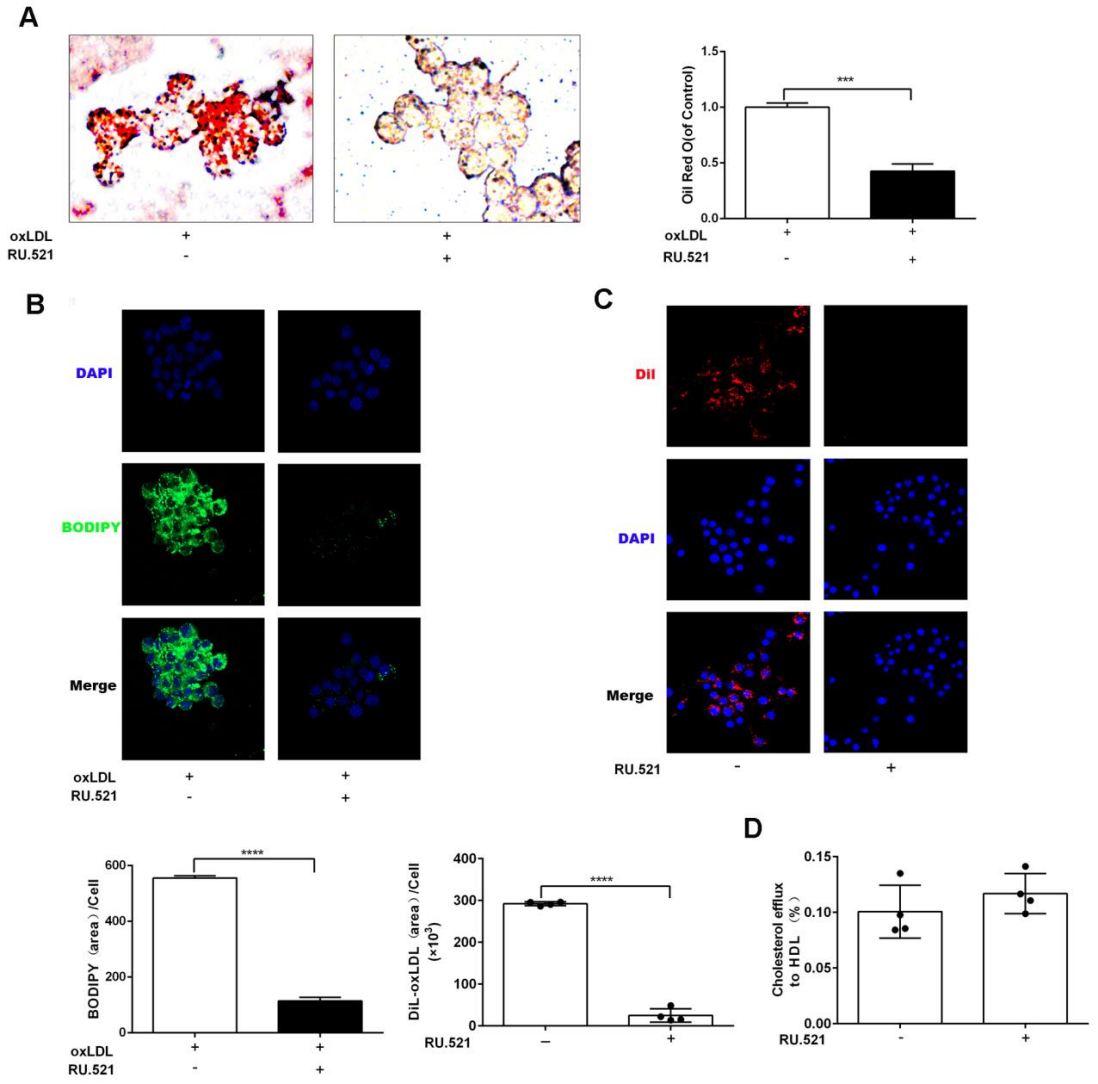
**cGAS inhibition reduced lipid deposition and foam cell formation in RAW264.7 macrophages.** RAW264.7 cells were treated for 24 hours with oxLDL(100 μg/mL) and with or without RU.521(cGAS inhibitor 2 μg/mL). (**A**) Oil Red-O staining or (**B**) BODIPY staining were used to identify lipid deposition and foam cell formation. RAW264.7 macrophages were treated with RU.521 (2 μg/mL) for 24 h before analysis. (**C**) Cholesterol uptake was determined using DiI-oxLDL (Scale bar: 20 μm). (**D**) HDL-mediated cholesterol efflux was assayed using NBD 485/535 (Scale bar: 20 μm). *** *P* < 0.001, **** *P* < 0.0001. Data are means ± SD.

To explore the effects of cGAS on atherosclerosis, ApoE ^-/-^ mice were kept on a western-type or chow diet for 16 weeks. Results demonstrated that cGAS was expressed in the plaque of ApoE ^-/-^ mice regardless of diet, and was positively correlated with plaque area, and mainly distributed in macrophages (Figure 2A, 2B), whereas, the protein expression of cGAS in the aorta of ApoE ^-/-^ mice fed a western-type diet was higher than that of ApoE ^-/-^ mice fed a chow diet (Figure 2C, 2D). Importantly, cGAS inhibition reduced lipid deposition and foam cell formation in RAW264.7 macrophages (Figure 3A, 3B). These data demonstrated that cGAS contributed to atherosclerosis, and suggested that exploring the underlying mechanism could be of great significance.

### Transcriptome profiling of RU.521-treated RAW264.7 macrophages

**Figure 4:**
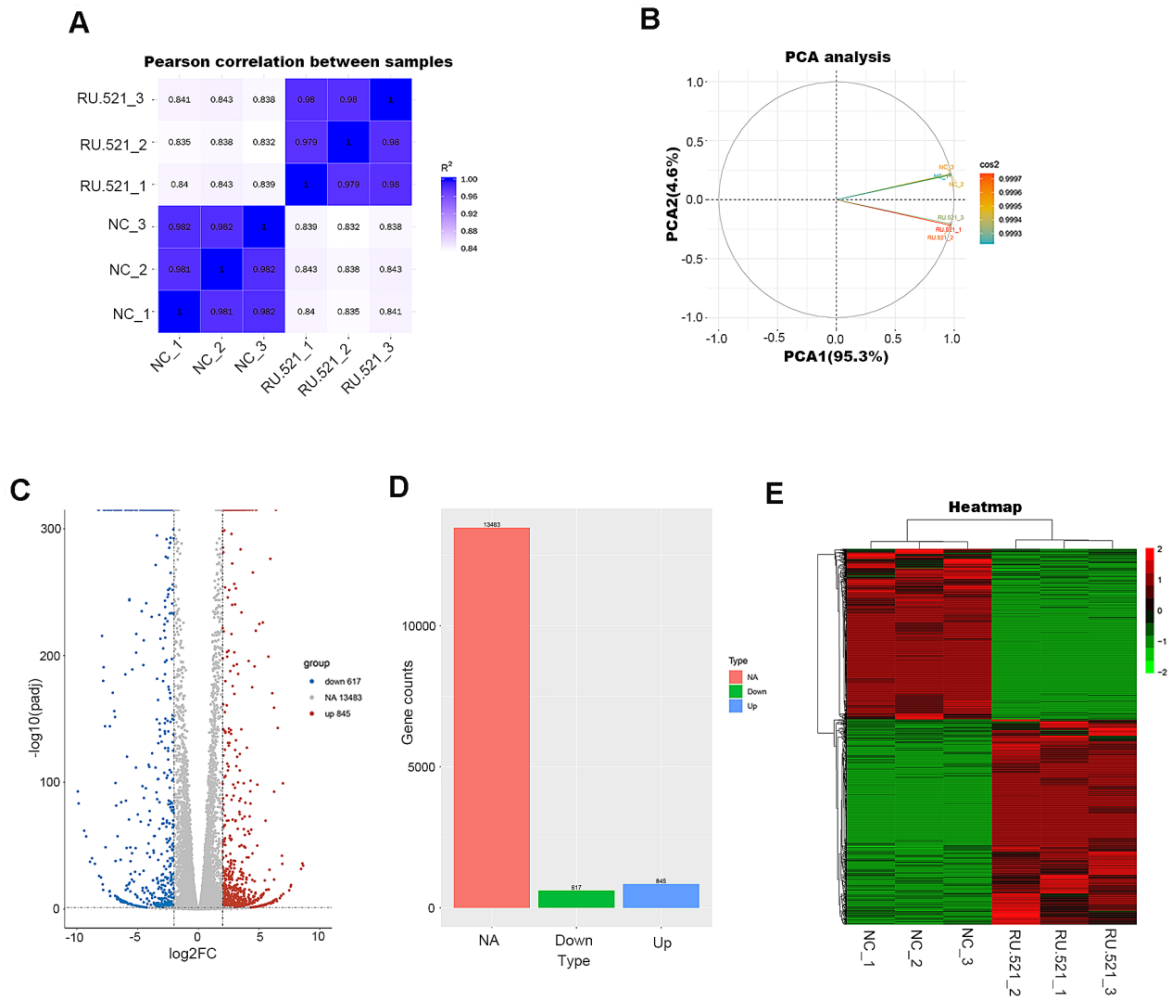
**Transcriptome profiling of RU.521-treated RAW264.7 macrophages.** (**A**) Pearson’s correlation analysis of samples. The color reflects the intensity of the correlation. The higher the correlation coefficient between samples, the closer the expression pattern. (**B**) PCA analysis of samples. Principal component 1 (PC1) and principal component 2 (PC2) are indicated on the X-axis and Y-axis, respectively. Cos2 stands for variables. The closer the two samples were to each other, the smaller the difference was between the two samples in terms of gene expression patterns. (**C**) Volcano plot of the DEGs. The X-axis represents the difference in log 2 conversion, the Y-axis represents the significant difference after log 10 conversion, the blue represents the down-regulated DEGs, the red represents the up-regulated DEGs, and the gray represents the non-DEGs. The DEGs were defined according to the criteria of log2FC ≥2 and *P*adj <0.005. (**D**) Bar graph showing DEGs. The X-axis represents the type, the Y-axis represents gene counts, the green represents the downregulated DEGs, the blue represents the upregulated DEGs, and the red is the non-DEGs. (**E**) Hierarchical clustering heatmap. Red and green represent high and low relative expression, respectively. Rows represent DEGs, and columns represent samples. DEGs, differentially-expressed genes; PCA, principal component analysis.

In order to have a better view of the mechanisms of atherosclerosis mediated by cGAS, we performed RNA-seq transcriptomic analysis of the underlying DEGs and the mechanisms in RAW264.7 macrophages treated for 12 h with or without RU.521, a cGAS inhibitor. The genes expressed were sequenced using the Illumia platform. DEGs between the cGAS inhibition group and the control group were detected. First, the intra-group samples showed a strong correlation (Figure 4A). Further, the repeatability of intra-group data based on principal component analysis (PCA) was acceptable, where the distances of intra-group samples were close. (Figure 4B).

A total of 1,462 DEGs were identified with the criteria of log2 (Fold Change, log2FC) ≥ 2 and *P*adj < 0.05, including 845 upregulated genes and 617 down-regulated genes (Figure 4C, 4D and Supplementary Table 2). Good distinction of DEGs between different groups was presented in the hierarchical clustering heatmap (Figure 4E).

### DEGs and functional enrichment analysis associated with atherosclerosis

In order to understand whether cGAS inhibition changes expression of the genes involved in modulating atherosclerosis, we identified DEGs in cGAS inhibition probably linked to atherosclerosis according to research published in the PubMed database. A total of 275 DEGs associated with atherosclerosis were found when cGAS was inhibited (Supplementary Table 3). These DEGs included colony-stimulating factors (*Csfs* such as *Csf2*, *Csf2rb*, *Csf1r,* and *Lif*.), interleukins (*Ils,* including *Il1b* ,*Il7*, *Il18*, *Il10*.), tumor necrosis factor family members (such as TNF superfamily members–*Tnfsf*–*Tnfsf10*, *Tnfsf8*, and *Tnfsf11a*.), toll-like receptors (*Tlrs* including *Tlr3*, *Tlr5*, and *Tlr7*), interferon regulatory factors (*Irfs*, *Irf5*, *Irf7*, and *Irf9*), genes related to cholesterol metabolism (*Msr1*, *Acat2*, *Apoa1*, *Abca1* and *Abcg5*), signal transducers and activators of transcription (*Stats*, such as *Stat1*, and *Stat2*), IFN and IFN-stimulated genes (*Isgs*, *Ifna*, *Ifnb*, *Ifit1*, *Ifit2*, and *Isg15*.) and so on. In addition, expression of *Il6, Tlr9, Irf3, Cd36, Acat1* and *Sting* were also decreased despite absolute log2FC < 2.

**Figure 5:**
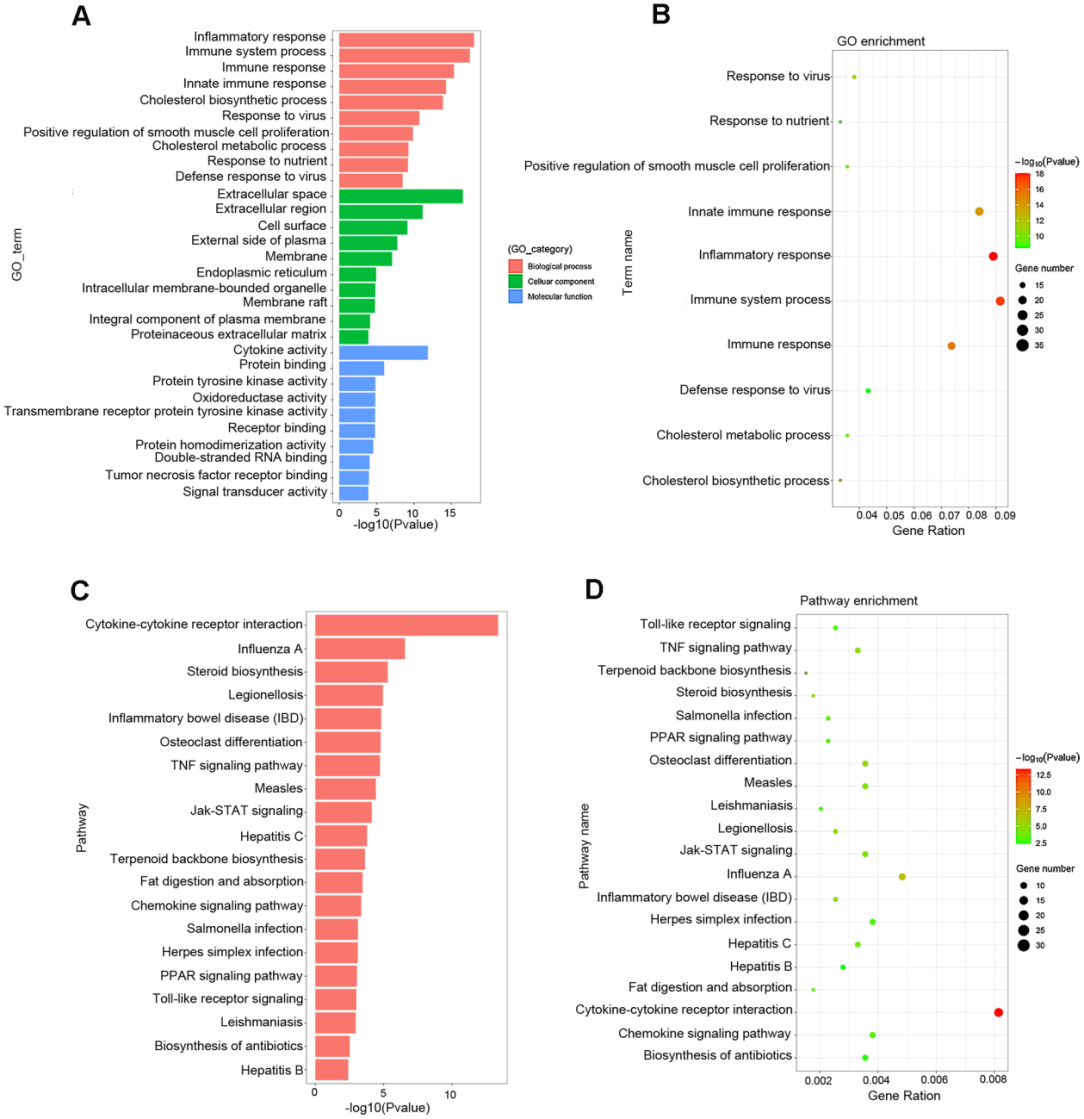
**Functional enrichment analysis associated with atherosclerosis.** (**A**, **B**) The GO function classification map and bubble chart of DEGs associated with atherosclerosis. (**C**, **D**) KEGG pathway classification map and bubble chart of DEGs. The X-axis represents the functional classification, and Y-axis represents the significant differences after the log 10 conversion or gene ratio. GO, Gene Ontology; KEGG, Kyoto Encyclopedia of Genes and Genomes.

Gene Ontology (GO) analysis showed that the main functional enrichments occurred in biological processes (BP), including inflammatory response, immune response, cholesterol biosynthetic process and so on, which are of great importance in the formation and progression of atherosclerotic plaque. In addition, GO terms linked to molecular function (MF) and cellular compartments (CC) were also discovered, such as extracellular space, cell surface, endoplasmic reticulum, and cytokine activity (Figure 5A, 5B and Table 3). Kyoto Encyclopedia of Genes and Genomes (KEGG) pathway analysis showed comparable results, with the most significant KEGG terms associated with atherosclerosis being cytokine–cytokine receptor interaction, Jak–STAT signaling pathway, steroid biosynthesis, chemokine signaling pathway, TNF signaling pathway, fat digestion and absorption, PPAR signaling pathway and TLR signaling pathway (Figure 5C, 5D and Table 4).

**Table 3:** GO enrichment analysis.

**Category**	**Term**	***P* value**	**Fold enrichment**
Biologyical process	GO:0006954~inflammatory response	0.000	6.942
Biologyical process	GO:0002376~immune system process	0.000	6.414
Biologyical process	GO:0006955~immune response	0.000	7.275
Biologyical process	GO:0045087~innate immune response	0.000	5.629
Biologyical process	GO:0006695~cholesterol biosynthetic process	0.000	27.720
Biologyical process	GO:0009615~response to virus	0.000	12.185
Biologyical process	GO:0048661~positive regulation of smooth muscle cell proliferation	0.000	11.941
Biologyical process	GO:0008203~cholesterol metabolic process	0.000	10.733
Biologyical process	GO:0007584~response to nutrient	0.000	12.151
Biologyical process	GO:0051607~defense response to virus	0.000	6.946
Celluar component	GO:0005615~extracellular space	0.000	3.196
Celluar component	GO:0005576~extracellular region	0.000	2.576
Celluar component	GO:0009986~cell surface	0.000	3.705
Celluar component	GO:0009897~external side of plasma membrane	0.000	4.824
Celluar component	GO:0016020~membrane	0.000	1.446
Celluar component	GO:0005783~endoplasmic reticulum	0.000	2.147
Celluar component	GO:0043231~intracellular membrane-bounded organelle	0.000	2.618
Celluar component	GO:0045121~membrane raft	0.000	4.169
Celluar component	GO:0005887~integral component of plasma membrane	0.000	2.134
Celluar component	GO:0005578~proteinaceous extracellular matrix	0.000	3.457
Molecular function	GO:0005125~cytokine activity	0.000	7.129
Molecular function	GO:0005515~protein binding	0.000	1.572
Molecular function	GO:0004713~protein tyrosine kinase activity	0.000	6.030
Molecular function	GO:0016491~oxidoreductase activity	0.000	2.746
Molecular function	GO:0004714~transmembrane receptor protein tyrosine kinase activity	0.000	9.827
Molecular function	GO:0005102~receptor binding	0.000	3.220
Molecular function	GO:0042803~protein homodimerization activity	0.000	2.411
Molecular function	GO:0003725~double-stranded RNA binding	0.000	7.581
Molecular function	GO:0005164~tumor necrosis factor receptor binding	0.000	12.438
Molecular function	GO:0004871~signal transducer activity	0.000	2.457

# 0.000 means < 0.0001.

**Table 4:** KEGG pathway analysis.

**Term**	***P* value**	**Fold enrichment**
mmu04060: Cytokine-cytokine receptor interaction	0.000	5.189
mmu05164: Influenza A	0.000	4.360
mmu00100: Steroid biosynthesis	0.000	14.457
mmu05134: Legionellosis	0.000	6.884
mmu05321: Inflammatory bowel disease (IBD)	0.000	6.651
mmu04380: Osteoclast differentiation	0.000	4.360
mmu04668: TNF signaling pathway	0.000	4.680
mmu05162: Measles	0.000	4.039
mmu04630: Jak-STAT signaling pathway	0.000	3.789
mmu05160: Hepatitis C	0.000	3.751
mmu00900: Terpenoid backbone biosynthesis	0.000	10.236
mmu04975: Fat digestion and absorption	0.000	7.228
mmu04062: Chemokine signaling pathway	0.000	3.003
mmu05132: Salmonella infection	0.001	4.528
mmu05168: Herpes simplex infection	0.001	2.830
mmu03320: PPAR signaling pathway	0.001	4.414
mmu04620: Toll-like receptor signaling pathway	0.001	3.885
mmu05140: Leishmaniasis	0.001	4.905
mmu01130: Biosynthesis of antibiotics	0.003	2.567
mmu05161: Hepatitis B	0.004	2.956

# 0.000 means <0.0001.

**Figure 6:**
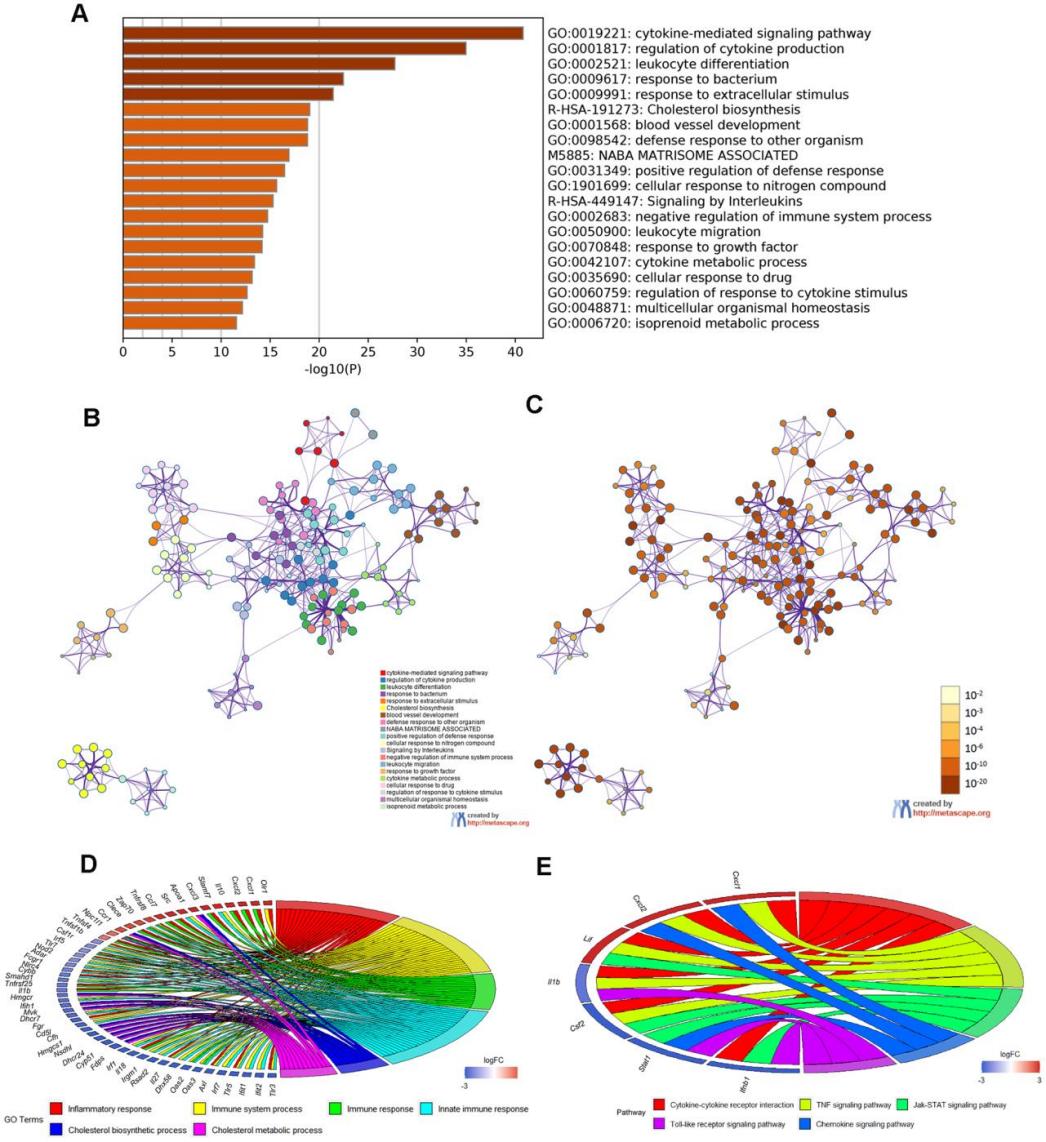
**Enrichment analysis of Metascape and overlapped DEGs.** (**A**) Bar graph of enriched terms, colored according to *P* value. Networks of enriched terms: (**B**) colored according to cluster ID and (**C**) colored according to *P* value. (**D**, **E**) Chord plot of the relationship between the overlapped DEGs and their corresponding GO and KEGG terms, together with the log2FC of the genes. Left half of Chord indicates whether the gene expression was up- or down-regulated. The right half represents different terms with different colors.

Moreover, Metascape analysis showed remarkable enrichment in regulation of the cytokine-mediated signaling pathway (Figure 6A–6C). There were 22 overlapped DEGs in both GO enriched terms “Inflammatory response” and “Immune system process”/ “Immune response”/ “Innate immune response” and seven overlapped DEGs in the KEGG analysis terms “Cytokine–cytokine receptor interaction”, “TNF signaling pathway”, “Jak–STAT signaling pathway”, “ Chemokine signaling pathway” and “Toll-like receptor signaling pathway”(Figure 6D, 6E and Tables 5, 6).

**Table 5:** GO enrichment analysis of overlapped DEGs.

**Gene**	**GO term**
*Olr1*	GO:0006954~inflammatory response
	GO:0002376~immune system process
*Cxcl1*	GO:0006954~inflammatory response
	GO:0006955~immune response
*Cxcl2*	GO:0006954~inflammatory response
	GO:0006955~immune response
*Il10*	GO:0006954~inflammatory response
	GO:0006955~immune response
*Cxcl3*	GO:0006954~inflammatory response
	GO:0006955~immune response
*Ccl7*	GO:0006954~inflammatory response
	GO:0006955~immune response
*Tnfrsf8*	GO:0006954~inflammatory response
	GO:0002376~immune system process
	GO:0006955~immune response
	GO:0045087~innate immune response
*Clece*	GO:0006954~inflammatory response
	GO:0006955~immune response
*Ccr1*	GO:0006954~inflammatory response
	GO:0006955~immune response
*Tnfsf4*	GO:0006954~inflammatory response
	GO:0006955~immune response
*Tnfrsf1b*	GO:0006954~inflammatory response
	GO:0006955~immune response
*Tlr7*	GO:0006954~inflammatory response
	GO:0002376~immune system process
	GO:0006955~immune response
	GO:0045087~innate immune response
*Nlrc4*	GO:0006954~inflammatory response
	GO:0045087~innate immune response
*Samhd1*	GO:0006954~inflammatory response
	GO:0006955~immune response
*Tnfrsf25*	GO:0006954~inflammatory response
	GO:0006955~immune response
*Il1b*	GO:0006954~inflammatory response
	GO:0006955~immune response
*Cd5l*	GO:0006954~inflammatory response
	GO:0002376~immune system process
*Il18*	GO:0006954~inflammatory response
	GO:0006955~immune response
*Il27*	GO:0006954~inflammatory response
	GO:0002376~immune system process
	GO:0045087~innate immune response
*Axl*	GO:0006954~inflammatory response
	GO:0006955~immune response
	GO:0045087~innate immune response
*Tlr5*	GO:0006954~inflammatory response
	GO:0006955~immune response
	GO:0045087~innate immune response
*Tlr3*	GO:0006954~inflammatory response
	GO:0006955~immune response
	GO:0045087~innate immune response

**Table 6:** KEGG pathway analysis of overlapped DEGs.

**Gene**	**KEGG pathway**
*Cxcl1*	mmu04060: Cytokine-cytokine receptor interaction
	mmu04668: TNF signaling pathway
	mmu04062: Chemokine signaling pathway
*Cxcl2*	mmu04060: Cytokine-cytokine receptor interaction
	mmu04668: TNF signaling pathway
	mmu04062: Chemokine signaling pathway
*Lif*	mmu04060: Cytokine-cytokine receptor interaction
	mmu04668: TNF signaling pathway
	mmu04630: Jak-STAT signaling pathway
*Il1b*	mmu04060: Cytokine-cytokine receptor interaction
	mmu04668: TNF signaling pathway
	mmu04620: Toll-like receptor signaling pathway
*Csf2*	mmu04060: Cytokine-cytokine receptor interaction
	mmu04668: TNF signaling pathway
*Stat1*	mmu04630: Jak-STAT signaling pathway
	mmu04062: Chemokine signaling pathway
	mmu04620: Toll-like receptor signaling pathway
*Ifnb1*	mmu04060: Cytokine-cytokine receptor interaction
	mmu04630: Jak-STAT signaling pathway
	mmu04620: Toll-like receptor signaling pathway

### Protein–protein interaction network of DEGs associated with atherosclerosis

**Figure 7:**
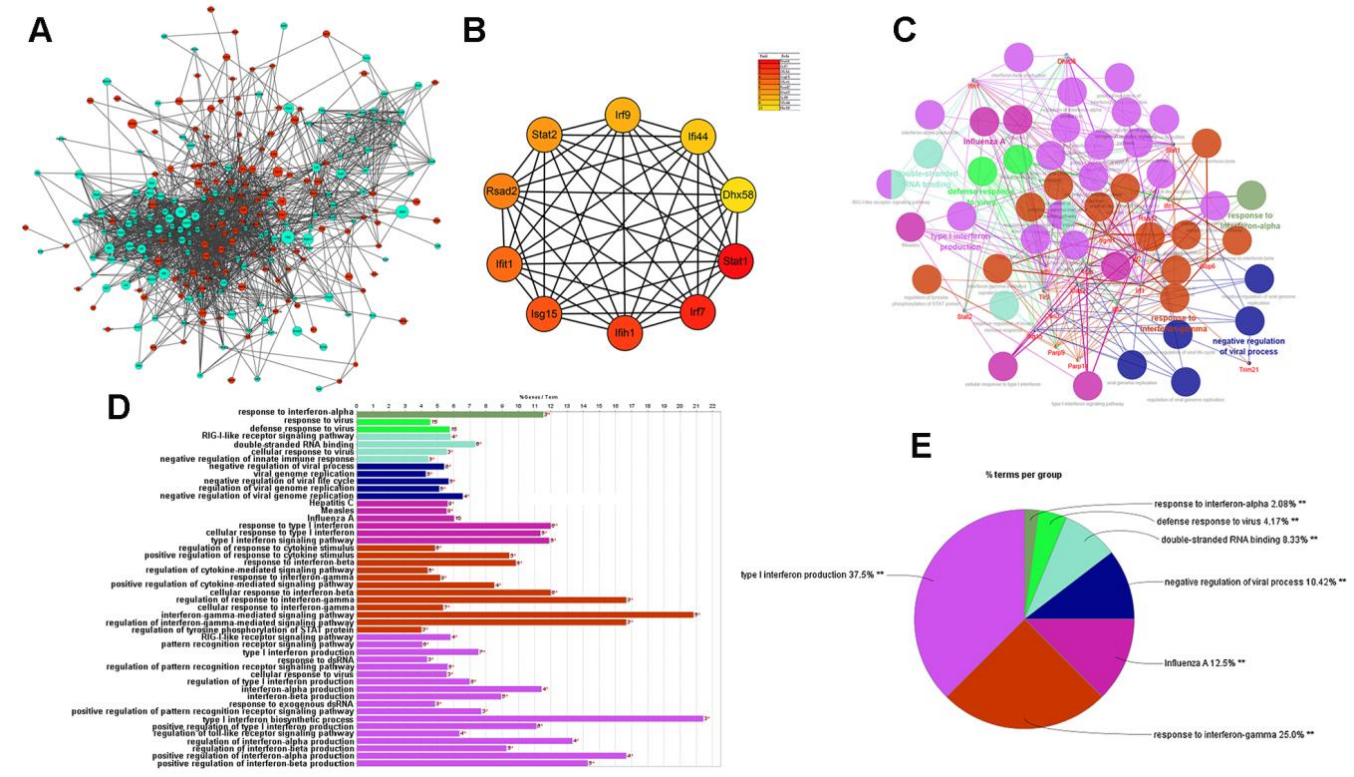
**Results of analysis of the PPI network associated with atherosclerosis.** (**A**) The PPI network of DEGs associated with atherosclerosis. The green circles represent down-regulated DEG-encoded proteins and the red circles represent upregulated DEG-encoded proteins. The size of the circle indicates the abundance of gene expression. (**B**) The top 10 hub genes in the PPI network by MCC method. (**C**–**E**) A function subnet module analysis performed by ClueGO. Terms are presented in different colors. PPI, Protein–Protein Interaction.

Next, the protein–protein interaction (PPI) network of DEGs was built as shown below (Figure 7A). The hub genes identified by the MCC method were identified as follows: *Stat1, Irf7, Ifih1, Isg15, Ifit1, Rsad2, Stat2, Irf9, Ifi44,* and *Dhx58*, exhibiting lower expression in cGAS inhibition (Figure 7B). A functional subnet module containing hub genes was selected from the PPI network and functional enrichment analysis was performed using ClueGO. The nodes in this module related to atherosclerosis were mainly enriched in the “response to interferon” (Figure 7C–7E).

### Verification of the genes expressed in synergistic inflammatory signaling

**Figure 8:**
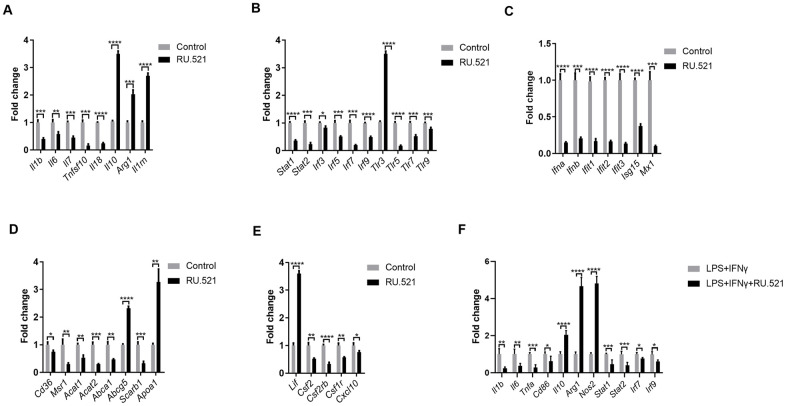
**Inflammatory signaling factors TLRs, STAT/IRF as well as IFNs were verified by RT-qPCR.** (**A**–**E**) RAW264.7 cells were treated for 12 h with RU.521 (2 μg/mL) or with DMSO as vehicle control. (**F**) RAW264.7 macrophages were pretreated with RU.521 for 12 h and then incubated for another 6 h with LPS (10 ng/mL) plus IFNγ (20 ng/mL) that polarizes macrophages to M1. Relative expression by RT-qPCR analysis. * *P* < 0.05, ** *P* < 0.01, *** *P* < 0.001, **** *P* < 0.0001, compared with control. Data are means ± SD. LPS, Lipopolysaccharide; IFNγ, interferon gamma; RT-qPCR, real time fluorescence quantitative PCR.

We sought to verify and extend the main findings suggested by RNA-seq. RAW264.7 cells were treated with or without the cGAS inhibitor RU.521(2 μg/mL) for 12 h and then real-time quantitative polymerase chain reaction (RT-qPCR) was performed. As shown in Figure 8A, a robust anti-inflammatory effect can be induced by cGAS inhibition via increasing the expression of anti-inflammatory factors (*Il10, Arg1, Lif, Il1rn*) and reducing inflammatory factors (*Il1b, Il7, Tnfsf10, Il18*). Additionally, *Il6* expression was also significantly decreased despite absolute log2FC < 2 in the RNA-seq results. In general, macrophages are classified as classical M1 or alternative M2. M1 macrophages are pro-inflammatory with high levels of cytokines such as IL 1B, IL6, and TNFα. Meanwhile M2 macrophages show anti-inflammatory properties, secreting anti-inflammatory factors, such as IL10 and ARG1. When stimulated by TLR ligands and IFNs, macrophages can transform into the M1 phenotype [22]. Furthermore, it is widely known that abnormal TLR signaling contributes to chronic inflammation [23]. Our results demonstrated that cGAS inhibition reduced the mRNA expression of *Tlrs* and, *Ifns* as well as the IFN-responsive genes (Figure 8B, 8C). These indicated that cGAS inhibition can restrain M1 polarization, promote M2 polarization, and that TLR or IFN signaling mediated by cGAS may participate in this process. Evidence suggests that M1 polarization can also be regulated by STAT1, IRF3, and IRF5 [24, 25], and inflammatory genes can be regulated by STAT1–IFN–IRF through over-expression in their promoters [26]. Previous studies have reported that STAT and IRF in the atherosclerotic tissues are displayed at significantly higher levels when compared with the matched normal tissues [27–31]. Here, *Stat* (*Stat1, Stat2*) and *Irf* (*Irf3, Irf5, Irf7, Irf9*) mRNA were significantly down regulated under cGAS inhibition (Figure 8B), consistent with the down regulation of inflammatory factors. RAW264.7 macrophages were pretreated with RU.521 for 12 h and then incubated for another 6 h with lipopolysaccharide (LPS, 10 ng/mL) plus interferon gamma (IFNγ, 20 ng/mL) that polarizes macrophages to the M1 phenotype. The results showed that cGAS significantly inhibited the M1 phenotype by decreasing *Il1b, Il6, Tnfa,* and *Cd86* while increasing *Il10, Arg1*, and *Nos2* mRNA expression which are characteristic of the M2 phenotype. Consistently, cGAS inhibition also inhibited the up-regulation of *Stat* and *Irf* in response to LPS plus IFNγ stimulation (Figure 8F). These results indicated that STAT and IRF signaling mediated by cGAS may be the key point of macrophage polarization through inflammatory regulation.

Given that foam cell formation acts as a hallmark of atherosclerosis, the expression of genes contributing to cholesterol uptake, cholesterol esterification and hydrolysis as well as cholesterol efflux was evaluated via RT-qPCR. cGAS inhibition blocked expression of *Cd36, Msr1, Acat1*, and *Acat2*, which mediate cholesterol uptake, cholesterol esterification and hydrolysis. In contrast, cGAS inhibition significantly increased the mRNA expression of *Apoa1* and *Abcg5*, which contribute to cholesterol efflux, consistent with the RNA-Seq results. However, cGAS failed to increase the expression of *Abca1* that promotes cholesterol efflux (Figure 8D). These results indicated that cGAS inhibition could suppress foam cell formation by hindering cholesterol uptake. Obviously, this assumption was confirmed in the RAW264.7 *in vitro* model (Figure 3C, 3D). In addition, the mRNA expression of colony-stimulating factors as well as chemokines was also verified (Figure 8E).

## DISCUSSION

As a chronic inflammatory disease, atherosclerosis shows the following characteristics: recruitment of circulating monocytes and their migration into the vascular intima, lipid accumulation, vascular local inflammation, smooth muscle cell (SMCs) proliferation, apoptosis, necrosis, and fibrosis [32–34]. Even in asymptomatic atherosclerosis, patients can suffer from coronary and carotid artery disease [35]. Therefore, identifying the molecular targets of atherosclerosis is imperative.

Following analysis of the microarray data of GSE40156 and GSE57614, cGAS was found to be increased in the total aorta of ApoE ^-/-^ mice and M1 macrophages. Consistently, our experiment first demonstrated that cGAS was expressed in the plaque of ApoE ^-/-^ mice regardless of diet, and was positively correlated with plaque area, mainly distributed in macrophages. Intriguingly, cGAS can also be found located in DAPI negative regions, and western blot results found that cGAS was clearly detected in the culture supernatant of RAW264.7 cells when treated with deoxyribonucleic acid sodium salt from herring testes (HT-DNA) or LPS plus IFNγ (Supplementary Figure 1), suggesting that cGAS may be secreted extracellularly. Further research is required to confirm this phenomenon and elaborate on how cGAS is secreted extracellularly. cGAS inhibition reduced foam cell formation in RAW264.7 macrophages and restrained M1 polarization as well as expression of inflammatory factors. Accordingly, cGAS may act as a critical molecule for the development of atherosclerosis via an inflammatory response mediated by macrophages. In order to reveal the possible mechanisms, RNA-seq was conducted in this research. A total of 1,462 DEGs were identified, 275 of which could be associated with atherosclerosis. Simultaneously, functional enrichment analysis and PPI network construction were performed on the 275 DEGs contributing to atherosclerosis, and some intriguing results were found. The DEGs were primarily concentrated in immune response, inflammatory response, and cytokine–cytokine receptor interaction signaling pathways, which have been proven to be critical in the development of atherosclerosis. We also found that the overlapped genes were mainly concentrated in the GO terms of “Immune response, Inflammatory response”, and “Cytokine–cytokine receptor interaction” pathways. The research results herein indicate that cGAS is probably a novel clue for the development of atherosclerosis through the inflammatory signaling pathway.

**Figure 9:**
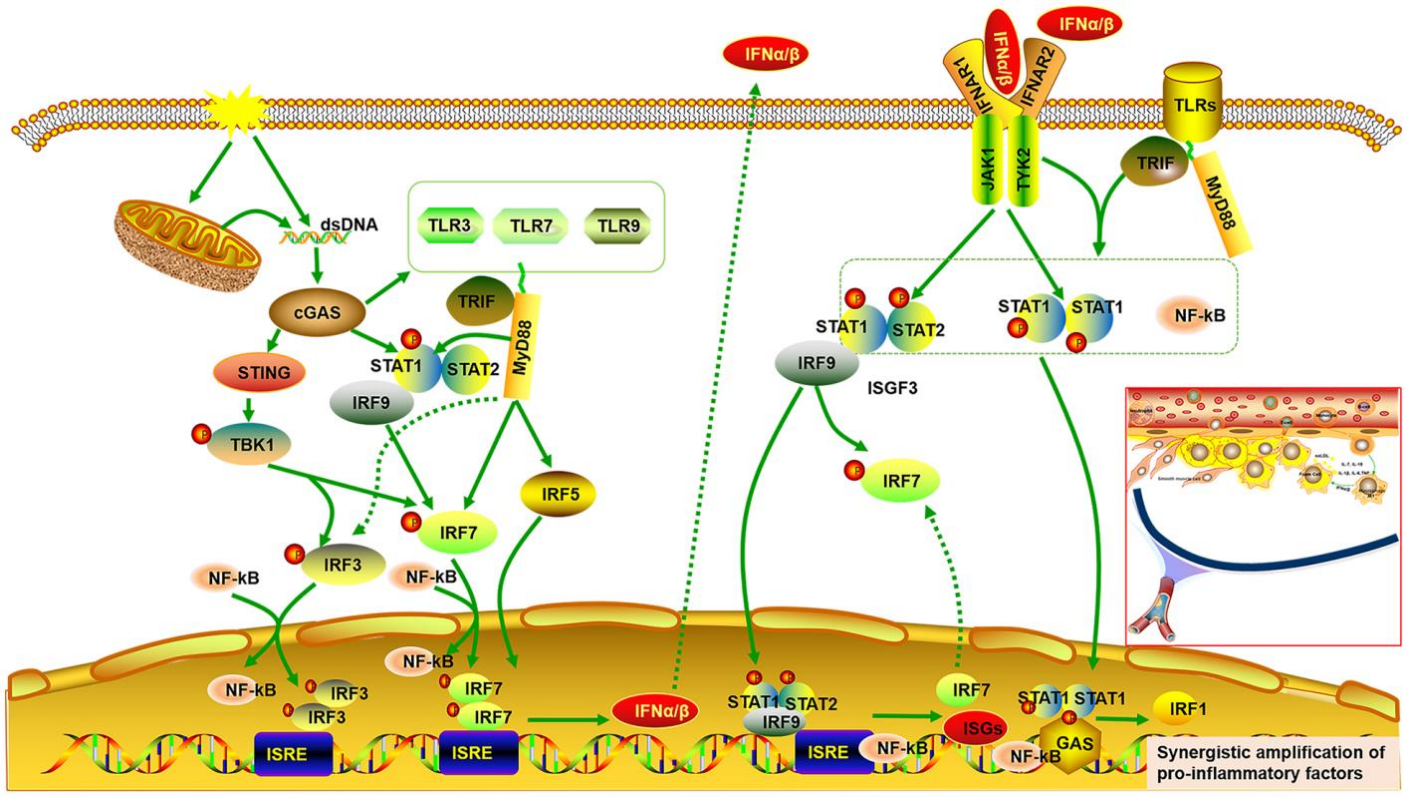
**Signal integrations of TLRs, STAT/IRF as well as type-I IFN exacerbate synergistic amplification of gene expression that leads to an inflammatory cascade and pro-atherogenic responses.** cGAS is activated by dsDNA and triggers the downstream TANK binding kinase (TBK)1, followed by phosphorylation of IRF3 as well as IRF7, which form homodimer and enter the nucleus from cytoplasm, accompany with or without other transcription factors such as STAT and NF-kB, thereby allowing initiation of the subsequent production of type I IFNα/β. On the other hand, the binding of type I IFN to the IFNα/β receptor (IFNAR)2 recruits IFNAR1. This complex enables activation of the receptor-associated JAK1 and tyrosine kinase (TYK)2, followed by STAT1 and STAT2 phosphorylation, which bind to IRF9, forming IFN-stimulated gene factor (ISGF)3 complex. The ISGF3 complex translocates into nucleus and promote the production of ISGs and IRF7 by binding to IFN-stimulated regulatory elements (ISRE) as well as IRF7 elements in DNA. Phosphorylated STAT1 can also form a homodimer, which binds to a comparable γ-activated sequence (GAS) in DNA, inducing the expression of IRF1 and pro-inflammatory genes. Moreover, cGAS can also result in up-regulated expression of TLRs (TLR3, TLR7, TLR9) and STAT (STAT1, STAT2). STAT1 phosphorylation can be induced by multiple TLRs dependent on MyD88 and TRIF signaling. Phosphorylated STAT1 translocates into the nucleus and augments TLR-NF-kB activation, promoting the expression of pro-inflammatory genes. Multiple TLRs also activate IRF5 and IRF7 as well as IRF3 via MyD88 and TRIF signaling.

It has been reported that atherosclerosis can be regulated by abnormal activation of the STAT and IRF signaling pathways [27, 28, 30, 36, 37]. It is universally acknowledged that inflammatory cytokines are mediators of atherosclerosis, and previous studies have demonstrated that inflammatory genes can be regulated by STAT1–NF-κB or STAT1–IRF through over-expression in their promoters [26]. Pro-inflammatory transcription can be enhanced by signal integration of IFN with TLR, which is involved in sequential recruitment of STAT1-complexes and NF-kB [38]. This suggests that type I IFN and TLR cross-talk could promote the process of atherogenesis. As the important hub genes, *Stat* and *Irf* mRNA expression were demonstrated to be reduced, accompanied by decreased expression of genes related to pro-inflammatory and immunoregulatory effects, and increased expression of genes related to anti-inflammatory effects. Meanwhile, cGAS inhibition also reduced expression of *Tlrs* and *Ifns*. Type I IFNs exert pro-inflammatory immune effects, subsequently promoting atherosclerosis [37, 39, 40]. Up-regulated ISGs are also detected in macrophages of mouse plaques, indicating a type-I IFN responsive subset [40]. Extensive study has shown that IRF3/7 activates the IFNα/β transcriptional promoter [40–42]. The cGAS–STING signaling pathway triggers the downstream TANK binding kinase (TBK)1, followed by phosphorylation of IRF3 as well as IRF7, which form homodimer and enter the nucleus from cytoplasm, accompany with or without other transcription factors, such as STAT and NF-kB, thereby allowing initiation of the subsequent production of CXCL10 and additional type I IFN [41, 43, 44]. On the other hand, the binding of type I IFN to the IFNα/β receptor (IFNAR)2 recruits IFNAR1. This complex enables activation of the receptor-associated JAK1 and tyrosine kinase (TYK)2, followed by STAT1 and STAT2 phosphorylation, which bind to IRF9, forming IFN-stimulated gene factor (ISGF)3 complex. The ISGF3 complex translocates into nucleus and promote the production of ISGs and IRF7 by binding to IFN-stimulated regulatory elements (ISRE) as well as IRF7 elements in DNA. Phosphorylated STAT1 can also form a homodimer, which binds to a comparable γ-activated sequence (GAS) in DNA, inducing the expression of IRF1 and pro-inflammatory genes [45, 46]. One study also found a positive correlation between lipid quantification by magnetic resonance imaging and the upregulation of genes of the IFN/STAT1 pathway [47]. TLRs, a crucial class of pattern recognition receptors contributing to inflammatory responses, can induce a subset of ISGs via activation of IRF [48]. Microbial membrane components such as lipoproteins can be recognized by cell surface TLRs. By contrast, the intracellular TLRs such as TLR3, TLR7, and TLR9 recognize pathogenic RNA or DNA [49]. In combination with our findings, these imply that cGAS inhibition can further downregulate IFNβ production and the subsequent inflammatory cascade by inhibiting TLRs. In addition, it has been reported that there is cross-talk between TLR and STAT signaling. STAT1 S727 phosphorylation can be induced by multiple TLRs dependent on MyD88/TRIF signaling instead of IRF and IFN signaling. Phosphorylated STAT1 transfers into the nucleus and augments TLR–NF-kB activation, promoting the expression of pro-inflammatory genes [50] (Figure 9).

Overall, signal integration of TLRs, STAT/IRF as well as IFN exacerbates synergistic amplification of gene expression, leading to inflammatory cascade and pro-atherogenic responses. It is well known that macrophages are of great importance in the initiation, progression and regression of atherosclerosis, and that different subtypes of macrophages affect the outcome of atherosclerosis. Classically, the pro-inflammatory M1 macrophage phenotype is responsible for atherosclerotic plaque vulnerability, whereas the anti-inflammatory M2 macrophage phenotype can increase atherosclerotic plaque stability [21]. Here, our result demonstrated that cGAS inhibition reduced expression of cytokines related to M1 polarization such as *Il1b* and *Il6*, while it increased expression of cytokines related to M2 polarization such as *Arg1* and *Il10*. Accumulating evidence suggests that STAT1, IRF3, and IRF5 contribute to M1 polarization. Conversely, STAT3, STAT6, and PPARγ contribute to M2 polarization [24, 25]. Moreover, the conversion from M2 to M1 can be regulated by activated IRF7 signaling [51]. IL10 inhibits production of IL1B and IL6 and promotes STAT3 phosphorylation [52–55], which causes the macrophage phenotype to revert from M1 to M2. Of note, LIF, a pleiotropic cytokine, has been proven to be anti-inflammatory, and increases IL10 expression, which can ameliorate atherosclerosis [56, 57]. Data have demonstrated that LIF is effective in not only inhibiting the formation of plaque but also retarding the progression of pre-existing atherosclerotic plaques in atherosclerosis. atherosclerosis [58].

APOA1, the main protein of high-density lipoprotein (HDL) which shows protective effects on atherosclerosis [59], was increased when cGAS was inhibited. Raising APOA1/HDL levels dampens the inflammatory activities of leukocytes and macrophages [60]. A previous study demonstrated that after infection with Salmonella (a Gram-negative bacterium that expresses LPS), the plasma levels of IFNβ significantly increase in ApoA1 deficient mice compared with wild-type mice [61]. RNA-seq transcriptomic analysis also showed that GO analysis for the rHDL-repressed genes revealed the following categories: cytokine biosynthetic process, cellular response to IFNβ, defense response to virus, and regulation of IL1B production [59]. Likewise, cGAS inhibition reduced *Cd36* and *Msr1*, by which the modified LDL is taken up. A previous study suggested STAT1 acetylation and its interaction with PPARγ induced by cholesterol crystals increase CD36 expression, which facilitates oxLDL uptake and foam cell formation [62]. Similarly, MSR1 upregulation is dependent on activation of the canonical Jak-STAT signaling pathway [63]. Furthermore, *Acat1* and *Acat2*, which mediate cholesterol esterification, were reduced when cGAS was inhibited, and their expression as well as overall activity in macrophages can be induced by IFNβ stimulation [64].

In conclusion, cGAS may contribute to the development of atherosclerosis. Gene expression profiling of cGAS inhibition in macrophages successfully identified important biological processes and pathways specific to atherosclerosis. The cGAS exacerbates the inflammatory cascade through synergistic signaling of TLRs, STAT, and IRF as well as IFN, triggering modulation of macrophage phenotypes to M1, thus increasing lipid deposition by up-regulating molecules related to cholesterol uptake. As the results are limited to sequencing analysis, a large number of experiments are still warranted. Despite this, these findings offer the theoretical foundation for future research into the underlying mechanisms of atherosclerosis and may provide potential therapeutic targets.

## MATERIALS AND METHODS

### Animals

Eight-week-old ApoE ^-/-^ male mice on a C57BL/6 background from Hua Fukang Experimental Animal Center (Beijing, China) were kept under standard conditions with constant humidity (approximately 40%) and temperature (approximately 22° C) as well as a 12-h light/dark cycle. A standard chow diet was offered for 1 week prior to all procedures, after which a western-type diet containing 0.21% cholesterol and 41 kCal% fat (Special Diet Services, Huafukang, China) or continuation on the standard chow diet was offered for 16weeks. Hearts and aortas were harvested for assessment of atherosclerotic burden.

### Cell culture and treatment

RAW 264.7 macrophages purchased from the Cell Resource Center of Shanghai Institute for Biological Sciences were grown in Dulbecco’s Modified Eagle’s Medium (DMEM, HyClone, Logan, UT, USA) containing 10% heat-inactivated fetal bovine serum (FBS, ScienCell Research Laboratories, Carlsbad, CA, USA), 1%penicillin and 1% streptomycin (HyClone). Cells were incubated under standard conditions with constant temperature (37° C) and an atmosphere of 5% CO_2_. Cells were grown to 60–80% confluence before use. In order to perform RNA-seq, cells were treated for 12 h with RU.521 (cGAS inhibitor 2 μg/mL, InvivoGen, San Diego, CA, USA) or with DMSO as vehicle control. In addition, RAW264.7 macrophages were pretreated with RU.521 (2 μg/mL) for 12 h and then incubated for another 6 h with LPS (10 ng/mL, HY-D1056, MedChemExpress, Monmouth Junction, NJ, USA) plus IFNγ (20 ng/mL, HY-P7071, MedChemExpress) that polarize macrophages to M1.

### Histologic and morphometric analysis

As previously described, mouse hearts were embedded in OCT and frozen at −80° C after perfusion, fixation, and dehydration. Then serial sections were cut at 10μm thickness using a cryostat (CryoStar NX50, Thermo Scientific, Waltham, USA). Hematoxylin and eosin (HE) staining of sections was performed for quantification of lesion areas.

### Immunofluorescent staining

Immunofluorescent staining was performed as previously described [65]. The primary antibodies used were as follows: cGAS (Mouse, 1:100, sc-515777, Santa Cruz Biotechnology, Santa Cruz, CA, USA), MOMA-2 (Rat, 1:100, ab33451, Abcam, Cambridge, UK), CD31 (1:100, AF3628, R&D Systems, Minneapolis, MN, USA) and α-SMA (Rabbit, 1:100, GB13044, Servicebio, Wuhan, China). 4′,6-diamidino-2-phenylindole (DAPI, Invitrogen, Carlsbad, CA, USA) was applied to stain nuclei. Fluorescent images were captured with a Nikon A1Si confocal microscope (Nikon, Tokyo, Japan), and analyzed with NIS Elements AR Imaging Software 4.10 (Nikon) and ImageJ 1.41 software (NIH, Bethesda, MD, USA).

### Dil-oxLDL uptake assay

Cells were treated with or without RU.521(2 μg/mL) for 24 h, followed by incubation with 20 μg/mL Dil-LDL (YB-0010, Yiyuan Corporation, Guangzhou, China) for 6 h or 8 h at 37° C. Then the cells were fixed and analyzed by immunofluorescent staining.

### Cholesterol efflux

Cholesterol efflux assays were performed as previously described [66]. Briefly, RAW264.7 macrophages were labeled with NBD (N1148, Life Technologies, Carlsbad, CA, USA) in the presence of RU.521 (2 μg/mL). After the cholesterol pools were equilibrated, cells were incubated with HDL (50 μg/mL, YB-003, Yiyuan Corporation) for 6 h. The fluorescence intensity of NBD was measured at 485nm excitation and 535nm emission. Efflux was measured as a percentage of fluorescence intensity in medium/(fluorescence intensity in medium + fluorescence intensity in cells) ×100%.

### Foam cell formation assays

RAW264.7 macrophages were incubated in DMEM containing oxLDL (100 μg/mL, YB-002, Yiyuan Corporation) with or without RU.521 (2 μg/mL) for 24 h. After fixation for 20 min, cells were blocked with 1% BSA, followed by staining with BODIPY 493/503 (D3922, Invitrogen) for 30 min. Next, nuclei visualized by staining DAPI for 10 min. Finally, antifade mounting medium (P0126, Beyotime Institute of Biotechnology, Jiangsu, China) was applied to prevent fluorescence quenching. Images were acquired using a Nikon A1Si confocal microscope. Analysis of different images was performed using ImageJ and Adobe Photoshop CC. Oil Red O staining was carried out as previously described [66, 67]. RAW264.7 macrophages were incubated in DMEM containing oxLDL (100 μg/mL) with or without RU.521 (2 μg/mL) for 24 h. After fixation for 30 min, cells were stained with oil red O (G1015, Servicebio) for 30 min, and then visualized by light microscopy (Olympus, Tokyo, Japan).

### Extraction and measurement of plasma dsDNA

Individuals (healthy volunteers and patients with atherosclerosis) were enrolled from Union Hospital, Tongji Medical College, Huazhong University of Science and Technology, Wuhan, China. Atherosclerosis was confirmed through ultrasonography or MRA/CTA/DSA imaging of the carotid or cerebral artery. Individuals with autoimmune diseases were excluded. Blood was collected and the plasma was isolated. Extracellular dsDNA in plasma was extracted using a DNA Extractor SP Kit (ZWK-296-60501, Wako Pure Chemical Industries Ltd., Osaka, Japan) and quantified using the Quant-iT™ PicoGreen ® dsDNA Reagent Kit (P7589, Invitrogen) following the manufacturer’s instructions.

### RNA extraction and real-time quantitative PCR analysis

Total cellular RNA was isolated and extracted using the TRIzol reagent (Vazyme, Nanjing, China). Quantitative RT-qPCR was carried out using the SYBR Green detection chemistry (Vazyme, Nanjing, China) in a 10μL reaction volume with an ABI 7500 Fast real-time PCR system. Glyceraldehyde 3-phosphate dehydrogenase (GAPDH) expression was used as the internal normalized reference gene. All samples were measured in triplicate, and the ΔΔCt method was applied to relative quantitative measurements. Primers are shown in Supplementary Table 1.

### Western blotting analysis

Western blotting analysis was conducted as previously described [65]. Primary antibodies against cGAS (Mouse, 1:1000, sc-515777, Santa Cruz Biotechnology) and, GAPDH (GAPDH, A01020, Abbkine, Redlands, CA, USA) were used. ImageJ software (NIH) was used to analyze protein expression levels which were normalized to GAPDH.

### Microarray data and data processing of DEGs

The microarray data of GSE40156 and GSE57614 derived from Gene Expression Omnibus (GEO) databases(https://www.ncbi.nlm.nih.gov/geo/query/acc.cgi?acc=GSE40156 and https://www.ncbi.nlm.nih.gov/geo/query/acc.cgi?acc=GSE57614) which were based on the GPL1261 [Mouse430_2] Affymetrix Mouse Genome 430 2.0 Array and GPL6480 Agilent-014850 Whole Human Genome Microarray 4x44K G4112F. DEGs were picked out using GEO2R online tools. A *P* value <0.05 and | log2FC | >1 were chosen as cut-off standards.

### RNA sequencing

RNA sequencing was performed following a previously published method [67]. Briefly, for each sample, a total amount of 1μg RNA was used as input material. The NEBNext^®^ Ultra™ RNA Library Prep Kit for Illumina^®^ (New England Biolabs (NEB), Ipswich, MA, USA) was applied to prepare sequencing libraries, followed by generation of clusters with the TruSeq PE Cluster Kit v3-cBot-HS (Illumina Inc., San Diego, CA, USA). The Illumina Novaseq platform was utilized to sequence libraries and then about 150 bp paired-end reads were obtained. Analysis of differential expression was performed using the edgeR R package. The Benjamini and Hochberg method was used to adjust *P* value. A *P*adj < 0.05 and | log2FC | ≥ 2 were set as the thresholds for significant differential expression.

### Functional enrichment analysis of DEGs

GO enrichment analysis and KEGG pathway analysis of DEGs was performed using the online tool DAVID (version: 6.8, https://david.ncifcrf.gov/) with thresholds of count ≥ 2 and *P* value < 0.05. Furthermore, Metascape (https://metascape.org/gp/index.html#/main/step1) was also used to obtain functional enrichment results.

### Protein–protein interaction network analysis of DEGs

The PPI network analysis of DEGs was based on the STRING database (https://string-db.org/), which functions as a tool for predicting protein–protein interactions. A threshold of PPI score (medium confidence) ≥ 0.4 was used to predict the DEG-encoded proteins. Then, Cytoscape software (version: 3.6.0) was used to visualize the PPI network. Module and hub gene analyses were carried out with MCODE, cytoHubba plugin as well as ClueGO of Cytoscape software.

### Statistical analyses

Results are expressed as mean ± standard deviation (SD). The analysis was completed using GraphPad Prism 6. In addition, the Student's t-test was used for comparison between the two groups. *P* values < 0.05 or *P*adj < 0.05 were considered statistically significant. R (3.6.2) software was used to display the distribution of DEGs as well as functional enrichment analysis.

### Ethics statement

This study was approved by the Medical Ethics Committee of Tongji Medical College and the Institutional Animal Care and Use Committee, Huazhong University of Science and Technology (HUST), Wuhan, China (S947, S2342). Written informed consents were obtained from Individuals (healthy volunteers and patients with atherosclerosis).

## Supplementary Material

Supplementary Figure 1

Supplementary Table 1

Supplementary Table 2

Supplementary Table 3
