# Supplementary material for: Synergistic inflammatory signaling by cGAS may be involved in the development of atherosclerosis
Source: Aging (Albany NY). 2021 Feb 11;13(4):5650–73. doi: 10.18632/aging.202491 (PMC7950297; doi:10.18632/aging.202491)
Supplement: Supplementary Figure 1 [file aging-13-202491-s001.pdf]

SUPPLEMENTARY FIGURE

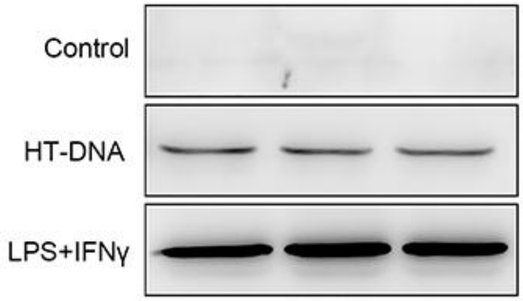

Supplementary Figure 1. cGAS may be secreted extracellularly. RAW264.7 cells were incubated for 12 h with either HT-DNA (8 µg/mL), or LPS (10 ng/mL) plus IFNγ (20 ng/ mL). The protein expression of cGAS in the culture supernatant was detected by western blotting (standardized at 60µg protein per sample). HT-DNA, deoxyribonucleic acid sodium salt from herring testes.
