# Supplementary material for: Synergistic inflammatory signaling by cGAS may be involved in the development of atherosclerosis
Source: Aging (Albany NY). 2021 Feb 11;13(4):5650–73. doi: 10.18632/aging.202491 (PMC7950297; doi:10.18632/aging.202491)
Supplement: Supplementary Table 1 [file aging-13-202491-s002.pdf]

Supplementary Table 1. List of primers.

Primer sequences	F	R
Apoa1	GGCACGTATGGCAGCAAGAT	CCAAGGAGGAGGATTCAAACTG
Cd36	GGAGGCATTCTCATGCCAGT	CTGCTGTTCTTTGCCACGTC
Msr1	GCACAATCTGTGATGATCGCT	CCCAGCATCTTCTGAATGTGAA
Acat1	TGGGTGCCACTTCGATGACT	TGATGTCACACCCACCATTG
Acat2	CCCGTGGTCATCGTCTCAG	GGACAGGGCACCATTGAAGG
Abca1	AACAGTTTGTGGCCCTTTTG	AGTTCCAGGCTGGGGTACTT
Abcg5	AGGGCCTCACATCAACAGAG	GCTGACGCTGTAGGACACAT
Scarb1	TTTGGAGTGGTAGTAAAAAGGGC	TGACATCAGGGACTCAGAGTAG
Il1b	GCAACTGTTCCTGAACTCAACT	ATCTTTTGGGGTCCGTCAACT
Il6	TAGTCCTTCCTACCCCAATTTCC	TTGGTCCTTAGCCACTCCTTC
Il7	TCAGCATCGATGAATTGGACA	GCGAGCAGCACGATTTAGAA
Tnfsf10	AGCAGCTGCAGGACAATTACT	CTGCAAGCAGGGTCTGTTCA
Tnfa	ATGTCTCAGCCTCTTCTCATTC	GCTTGTCACTCGAATTTTGAGA
Cd86	AGCTTCAGTTACTGTGGCCC	TCAGCGTTACTATCCCGCTC
Ifna	TTTCCCCTGACCCAGGAAGA	CTTCTGCTCTGACCACCTCC
Ifnb	CAGCTCCAAGAAAGGACGAAC	GGCAGTGTAACTCTTCTGCAT
Il18	CGACTTCACTGTACAACCGC	GGGGTTCACTGGCACTTTGA
Il10	TTCTTTCAAACAAAGGACCAGC	GCAACCCAAGTAACCCTTAAAG
Arg1	CTCCAAGCCAAAGTCCTTAGAG	AGGAGCTGTCATTAGGGACATC
Nos2	GGAGTGACGGCAAACATGACT	TCGATGCACAACTGGGTGAAC
Il1rn	GGGGACCCTACAGTCACCTAAT	GCTTGCATCTTGCAGGGTCT
Lif	AGAAGGTCCTGAACCCCACT	CCACACGGTACTTGTTGCAC
Csf2	GGCCTTGGAAGCATGTAGAGG	GGAGAACTCGTTAGAGACGACTT
Csf2rb	GGAGCAAGTGGAGCGAAGAG	TACAGAGACACAGCCAAAGCG
Csf1r	TGTCATCGAGCCTAGTGGC	CGGGAGATTCAGGGTCCAAG
Cxcl10	CAACTGCATCCATATCGATGAC	GATTCCGGATTCAGACATCTCT
Stat1	TCACAGTGGTTCGAGCTTCAG	GCAAACGAGACATCATAGGCA
Stat2	TCCTGCCAATGGACGTTCG	GTCCCACTGGTTCAGTTGGT
Irf3	GAGAGCCGAACGAGGTTCAG	CTTCCAGGTTGACACGTCCG
Irf5	GGTCAACGGGGAAAAGAAACT	CATCCACCCCTTCAGTGTACT
Irf7	GAGACTGGCTATTGGGGGAG	GACCGAAATGCTTCCAGGG
Irf9	GCCGAGTGGTGGGTAAGAC	GCAAAGGCGCTGAACAAAGAG
Tlr3	GTGAGATACAACGTAGCTGACTG	TCCTGCATCCAAGATAGCAAGT
Tlr5	GCCCCGTGTTGGTAATATCTC	ATCTGGGTGAGGTTACAGCCT
Tlr7	ATGTGGACACGGAAGAGACAA	GGTAAGGGTAAGATTGGTGGTG
Tlr9	ATGGTTCTCCGTCGAAGGACT	GAGGCTTCAGCTCACAGGG
Ifit1	CTGAGATGTCACTTCACATGGAA	GTGCATCCCCAATGGGTTCT
Ifit2	AGTACAACGAGTAAGGAGTCACT	AGGCCAGTATGTTGCACATGG
Ifit3	TCCTCCAAAAGGCAGCTCAG	ACGGCACATGACCAAAGAGT
Isg15	GGTGTCCGTGACTAACTCCAT	TGGAAAGGGTAAGACCGTCCT
Mx1	GACCATAGGGGTCTTGACCAA	AGACTTGCTCTTTCTGAAAAGCC
