# Supplementary material for: Synergistic inflammatory signaling by cGAS may be involved in the development of atherosclerosis
Source: Aging (Albany NY). 2021 Feb 11;13(4):5650–73. doi: 10.18632/aging.202491 (PMC7950297; doi:10.18632/aging.202491)
Supplement: Supplementary Table 2 [file aging-13-202491-s003.docx]

**Supplementary Table 2. The list of DEGs.**

| **Gene_ID** | **Gene_name** | **log2FC** | ***P* value** | ***P*adj** |
| --- | --- | --- | --- | --- |
| ENSMUSG00000015837 | *Sqstm1* | 2.671 | 0.000 | 0.000 |
| ENSMUSG00000070327 | *Rnf213* | -3.729 | 0.000 | 0.000 |
| ENSMUSG00000028691 | *Prdx1* | 2.246 | 0.000 | 0.000 |
| ENSMUSG00000058427 | *Cxcl2* | 4.226 | 0.000 | 0.000 |
| ENSMUSG00000025203 | *Scd2* | -4.570 | 0.000 | 0.000 |
| ENSMUSG00000005413 | *Hmox1* | 2.911 | 0.000 | 0.000 |
| ENSMUSG00000024621 | *Csf1r* | -2.026 | 0.000 | 0.000 |
| ENSMUSG00000015568 | *Lpl* | -3.524 | 0.000 | 0.000 |
| ENSMUSG00000032487 | *Ptgs2* | 2.184 | 0.000 | 0.000 |
| ENSMUSG00000003541 | *Ier3* | 3.404 | 0.000 | 0.000 |
| ENSMUSG00000027737 | *Slc7a11* | 2.693 | 0.000 | 0.000 |
| ENSMUSG00000040435 | *Ppp1r15a* | 2.233 | 0.000 | 0.000 |
| ENSMUSG00000022587 | *Ly6e* | -2.839 | 0.000 | 0.000 |
| ENSMUSG00000096054 | *Syne1* | 2.509 | 0.000 | 0.000 |
| ENSMUSG00000037169 | *Mycn* | -2.263 | 0.000 | 0.000 |
| ENSMUSG00000027580 | *Helz2* | -2.866 | 0.000 | 0.000 |
| ENSMUSG00000035969 | *Rusc2* | 2.069 | 0.000 | 0.000 |
| ENSMUSG00000028124 | *Gclm* | 2.144 | 0.000 | 0.000 |
| ENSMUSG00000027009 | *Itga4* | -2.867 | 0.000 | 0.000 |
| ENSMUSG00000024308 | *Tapbp* | -2.779 | 0.000 | 0.000 |
| ENSMUSG00000034422 | *Parp14* | -5.062 | 0.000 | 0.000 |
| ENSMUSG00000039501 | *Znfx1* | -2.988 | 0.000 | 0.000 |
| ENSMUSG00000032661 | *Oas3* | -5.934 | 0.000 | 0.000 |
| ENSMUSG00000027763 | *Mbnl1* | -2.321 | 0.000 | 0.000 |
| ENSMUSG00000004266 | *Ptpn6* | -2.376 | 0.000 | 0.000 |
| ENSMUSG00000033066 | *Gas7* | -2.775 | 0.000 | 0.000 |
| ENSMUSG00000027639 | *Samhd1* | -2.469 | 0.000 | 0.000 |
| ENSMUSG00000030275 | *Etnk1* | -2.091 | 0.000 | 0.000 |
| ENSMUSG00000021670 | *Hmgcr* | -2.641 | 0.000 | 0.000 |
| ENSMUSG00000030142 | *Clec4e* | 2.664 | 0.000 | 0.000 |
| ENSMUSG00000045932 | *Ifit2* | -7.743 | 0.000 | 0.000 |
| ENSMUSG00000005125 | *Ndrg1* | 3.707 | 0.000 | 0.000 |
| ENSMUSG00000025498 | *Irf7* | -6.012 | 0.000 | 0.000 |
| ENSMUSG00000010663 | *Fads1* | -2.104 | 0.000 | 0.000 |
| ENSMUSG00000060519 | *Tor3a* | -2.092 | 0.000 | 0.000 |
| ENSMUSG00000027951 | *Adar* | -2.102 | 0.000 | 0.000 |
| ENSMUSG00000026981 | *Il1rn* | 2.817 | 0.000 | 0.000 |
| ENSMUSG00000021306 | *Gpr137b* | 2.320 | 0.000 | 0.000 |
| ENSMUSG00000056429 | *Tgoln1* | 2.013 | 0.000 | 0.000 |
| ENSMUSG00000026833 | *Olfm1* | -2.509 | 0.000 | 0.000 |
| ENSMUSG00000070348 | *Ccnd1* | -3.485 | 0.000 | 0.000 |
| ENSMUSG00000038507 | *Parp12* | -4.544 | 0.000 | 0.000 |
| ENSMUSG00000020300 | *Cpeb4* | 2.353 | 0.000 | 0.000 |
| ENSMUSG00000046718 | *Bst2* | -3.199 | 0.000 | 0.000 |
| ENSMUSG00000032802 | *Srxn1* | 2.293 | 0.000 | 0.000 |
| ENSMUSG00000052684 | *Jun* | 2.678 | 0.000 | 0.000 |
| ENSMUSG00000024665 | *Fads2* | -3.542 | 0.000 | 0.000 |
| ENSMUSG00000020190 | *Mknk2* | 2.362 | 0.000 | 0.000 |
| ENSMUSG00000032193 | *Ldlr* | -5.955 | 0.000 | 0.000 |
| ENSMUSG00000040296 | *Ddx58* | -5.452 | 0.000 | 0.000 |
| ENSMUSG00000028037 | *Ifi44* | -6.939 | 0.000 | 0.000 |
| ENSMUSG00000002307 | *Daxx* | -2.562 | 0.000 | 0.000 |
| ENSMUSG00000030107 | *Usp18* | -6.672 | 0.000 | 0.000 |
| ENSMUSG00000036986 | *Pml* | -4.424 | 0.000 | 0.000 |
| ENSMUSG00000042726 | *Trafd1* | -3.093 | 0.000 | 0.000 |
| ENSMUSG00000028494 | *Plin2* | 2.060 | 0.000 | 0.000 |
| ENSMUSG00000015340 | *Cybb* | -2.465 | 0.000 | 0.000 |
| ENSMUSG00000019558 | *Slc6a8* | 3.101 | 0.000 | 0.000 |
| ENSMUSG00000046879 | *Irgm1* | -4.777 | 0.000 | 0.000 |
| ENSMUSG00000047735 | *Samd9l* | -3.106 | 0.000 | 0.000 |
| ENSMUSG00000033105 | *Lss* | -2.437 | 0.000 | 0.000 |
| ENSMUSG00000015947 | *Fcgr1* | -2.239 | 0.000 | 0.000 |
| ENSMUSG00000032690 | *Oas2* | -5.713 | 0.000 | 0.000 |
| ENSMUSG00000001467 | *Cyp51* | -3.720 | 0.000 | 0.000 |
| ENSMUSG00000025757 | *Hspa4l* | 2.692 | 0.000 | 0.000 |
| ENSMUSG00000022351 | *Sqle* | -2.406 | 0.000 | 0.000 |
| ENSMUSG00000036103 | *Colec12* | 2.140 | 0.000 | 0.000 |
| ENSMUSG00000053581 | *Zfand2a* | 2.319 | 0.000 | 0.000 |
| ENSMUSG00000028459 | *Cd72* | -2.100 | 0.000 | 0.000 |
| ENSMUSG00000035441 | *Myo1d* | 2.092 | 0.000 | 0.000 |
| ENSMUSG00000029762 | *Akr1b8* | 2.043 | 0.000 | 0.000 |
| ENSMUSG00000040033 | *Stat2* | -3.686 | 0.000 | 0.000 |
| ENSMUSG00000020641 | *Rsad2* | -5.071 | 0.000 | 0.000 |
| ENSMUSG00000032515 | *Csrnp1* | 2.524 | 0.000 | 0.000 |
| ENSMUSG00000054364 | *Rhob* | 2.232 | 0.000 | 0.000 |
| ENSMUSG00000024190 | *Dusp1* | 4.728 | 0.000 | 0.000 |
| ENSMUSG00000052776 | *Oas1a* | -2.746 | 0.000 | 0.000 |
| ENSMUSG00000022102 | *Dok2* | -2.251 | 0.000 | 0.000 |
| ENSMUSG00000053835 | *H2-T24* | -6.743 | 0.000 | 0.000 |
| ENSMUSG00000052160 | *Pld4* | -2.344 | 0.000 | 0.000 |
| ENSMUSG00000055675 | *Kbtbd11* | -3.395 | 0.000 | 0.000 |
| ENSMUSG00000035692 | *Isg15* | -3.323 | 0.000 | 0.000 |
| ENSMUSG00000063268 | *Parp10* | -4.420 | 0.000 | 0.000 |
| ENSMUSG00000033880 | *Lgals3bp* | -5.499 | 0.000 | 0.000 |
| ENSMUSG00000069516 | *Lyz2* | -2.002 | 0.000 | 0.000 |
| ENSMUSG00000022150 | *Dab2* | -2.266 | 0.000 | 0.000 |
| ENSMUSG00000024079 | *Eif2ak2* | -3.970 | 0.000 | 0.000 |
| ENSMUSG00000078566 | *Bnip3* | 3.574 | 0.000 | 0.000 |
| ENSMUSG00000096210 | *H1f0* | -2.121 | 0.000 | 0.000 |
| ENSMUSG00000049401 | *Ogfr* | -2.001 | 0.000 | 0.000 |
| ENSMUSG00000026355 | *Mcm6* | -2.082 | 0.000 | 0.000 |
| ENSMUSG00000054404 | *Slfn5* | -5.625 | 0.000 | 0.000 |
| ENSMUSG00000026896 | *Ifih1* | -2.693 | 0.000 | 0.000 |
| ENSMUSG00000053470 | *Kdm3a* | 2.461 | 0.000 | 0.000 |
| ENSMUSG00000029771 | *Irf5* | -2.048 | 0.000 | 0.000 |
| ENSMUSG00000037731 | *Themis2* | -4.157 | 0.000 | 0.000 |
| ENSMUSG00000020638 | *Cmpk2* | -7.681 | 0.000 | 0.000 |
| ENSMUSG00000004880 | *Lbr* | -2.436 | 0.000 | 0.000 |
| ENSMUSG00000034926 | *Dhcr24* | -3.700 | 0.000 | 0.000 |
| ENSMUSG00000032344 | *Mb21d1* | -2.627 | 0.000 | 0.000 |
| ENSMUSG00000042622 | *Maff* | 4.477 | 0.000 | 0.000 |
| ENSMUSG00000002233 | *Rhoc* | 2.041 | 0.000 | 0.000 |
| ENSMUSG00000034459 | *Ifit1* | -7.153 | 0.000 | 0.000 |
| ENSMUSG00000002227 | *Mov10* | -3.194 | 0.000 | 0.000 |
| ENSMUSG00000022906 | *Parp9* | -4.015 | 0.000 | 0.000 |
| ENSMUSG00000017830 | *Dhx58* | -5.650 | 0.000 | 0.000 |
| ENSMUSG00000005583 | *Mef2c* | -3.227 | 0.000 | 0.000 |
| ENSMUSG00000029561 | *Oasl2* | -6.770 | 0.000 | 0.000 |
| ENSMUSG00000041926 | *Rnpep* | -2.603 | 0.000 | 0.000 |
| ENSMUSG00000031387 | *Renbp* | 2.129 | 0.000 | 0.000 |
| ENSMUSG00000040511 | *Pvr* | 2.271 | 0.000 | 0.000 |
| ENSMUSG00000037321 | *Tap1* | -5.204 | 0.000 | 0.000 |
| ENSMUSG00000037936 | *Scarb1* | -3.033 | 0.000 | 0.000 |
| ENSMUSG00000004508 | *Gab2* | 2.349 | 0.000 | 0.000 |
| ENSMUSG00000066621 | *Tecpr1* | 2.307 | 0.000 | 0.000 |
| ENSMUSG00000055013 | *Agap1* | 2.049 | 0.000 | 0.000 |
| ENSMUSG00000037071 | *Scd1* | -6.491 | 0.000 | 0.000 |
| ENSMUSG00000035208 | *Slfn8* | -3.104 | 0.000 | 0.000 |
| ENSMUSG00000028680 | *Plk3* | 2.409 | 0.000 | 0.000 |
| ENSMUSG00000014905 | *Dnajb9* | 2.143 | 0.000 | 0.000 |
| ENSMUSG00000026222 | *Sp100* | -4.752 | 0.000 | 0.000 |
| ENSMUSG00000019082 | *Slc25a22* | -3.008 | 0.000 | 0.000 |
| ENSMUSG00000032596 | *Uba7* | -5.525 | 0.000 | 0.000 |
| ENSMUSG00000030103 | *Bhlhe40* | 3.738 | 0.000 | 0.000 |
| ENSMUSG00000049502 | *Dtx3l* | -4.454 | 0.000 | 0.000 |
| ENSMUSG00000067212 | *H2-T23* | -2.682 | 0.000 | 0.000 |
| ENSMUSG00000027611 | *Procr* | 3.667 | 0.000 | 0.000 |
| ENSMUSG00000027078 | *Ube2l6* | -3.593 | 0.000 | 0.000 |
| ENSMUSG00000026785 | *Pkn3* | 2.770 | 0.000 | 0.000 |
| ENSMUSG00000029919 | *Hpgds* | 2.565 | 0.000 | 0.000 |
| ENSMUSG00000040128 | *Pnrc1* | 3.195 | 0.000 | 0.000 |
| ENSMUSG00000037411 | *Serpine1* | 4.053 | 0.000 | 0.000 |
| ENSMUSG00000039936 | *Pik3cd* | -2.801 | 0.000 | 0.000 |
| ENSMUSG00000078771 | *Evi2a* | -2.280 | 0.000 | 0.000 |
| ENSMUSG00000074896 | *Ifit3* | -7.773 | 0.000 | 0.000 |
| ENSMUSG00000022216 | *Psme1* | -2.481 | 0.000 | 0.000 |
| ENSMUSG00000029816 | *Gpnmb* | 2.010 | 0.000 | 0.000 |
| ENSMUSG00000068245 | *Phf11d* | -6.643 | 0.000 | 0.000 |
| ENSMUSG00000058755 | *Osm* | 2.957 | 0.000 | 0.000 |
| ENSMUSG00000022051 | *Bnip3l* | 2.447 | 0.000 | 0.000 |
| ENSMUSG00000049932 | *H2afx* | -2.709 | 0.000 | 0.000 |
| ENSMUSG00000034041 | *Lyl1* | -2.815 | 0.000 | 0.000 |
| ENSMUSG00000093930 | *Hmgcs1* | -2.911 | 0.000 | 0.000 |
| ENSMUSG00000005686 | *Ampd3* | 3.868 | 0.000 | 0.000 |
| ENSMUSG00000031897 | *Psmb10* | -3.832 | 0.000 | 0.000 |
| ENSMUSG00000030091 | *Nup210* | -3.044 | 0.000 | 0.000 |
| ENSMUSG00000040204 | *Pclaf* | -2.727 | 0.000 | 0.000 |
| ENSMUSG00000034118 | *Tpst1* | -3.161 | 0.000 | 0.000 |
| ENSMUSG00000022951 | *Rcan1* | 2.455 | 0.000 | 0.000 |
| ENSMUSG00000027959 | *Sass6* | -2.913 | 0.000 | 0.000 |
| ENSMUSG00000004267 | *Eno2* | 4.779 | 0.000 | 0.000 |
| ENSMUSG00000018217 | *Pmp22* | 2.216 | 0.000 | 0.000 |
| ENSMUSG00000031453 | *Rasa3* | -2.305 | 0.000 | 0.000 |
| ENSMUSG00000035517 | *Tdrd7* | -2.377 | 0.000 | 0.000 |
| ENSMUSG00000031266 | *Gla* | 2.466 | 0.000 | 0.000 |
| ENSMUSG00000021752 | *Kctd6* | 2.928 | 0.000 | 0.000 |
| ENSMUSG00000039236 | *Isg20* | -4.061 | 0.000 | 0.000 |
| ENSMUSG00000039146 | *Ifi44l* | -7.668 | 0.000 | 0.000 |
| ENSMUSG00000054499 | *Dedd2* | 2.919 | 0.000 | 0.000 |
| ENSMUSG00000033355 | *Rtp4* | -5.416 | 0.000 | 0.000 |
| ENSMUSG00000024399 | *Ltb* | -3.237 | 0.000 | 0.000 |
| ENSMUSG00000050022 | *Amz1* | 2.682 | 0.000 | 0.000 |
| ENSMUSG00000069793 | *Slfn9* | -4.437 | 0.000 | 0.000 |
| ENSMUSG00000072812 | *Ahnak2* | 3.066 | 0.000 | 0.000 |
| ENSMUSG00000023832 | *Acat2* | -3.773 | 0.000 | 0.000 |
| ENSMUSG00000038550 | *Ciart* | 3.303 | 0.000 | 0.000 |
| ENSMUSG00000001281 | *Itgb7* | 2.749 | 0.000 | 0.000 |
| ENSMUSG00000070034 | *Sp110* | -3.964 | 0.000 | 0.000 |
| ENSMUSG00000026675 | *Hsd17b7* | -2.593 | 0.000 | 0.000 |
| ENSMUSG00000023009 | *Nckap5l* | 2.220 | 0.000 | 0.000 |
| ENSMUSG00000058258 | *Idi1* | -3.965 | 0.000 | 0.000 |
| ENSMUSG00000039994 | *Timeless* | -2.682 | 0.000 | 0.000 |
| ENSMUSG00000040253 | *Gbp7* | -7.276 | 0.000 | 0.000 |
| ENSMUSG00000028885 | *Smpdl3b* | -3.068 | 0.000 | 0.000 |
| ENSMUSG00000026321 | *Tnfrsf11a* | -2.930 | 0.000 | 0.000 |
| ENSMUSG00000001123 | *Lgals9* | -5.905 | 0.000 | 0.000 |
| ENSMUSG00000026535 | *Ifi202b* | -3.157 | 0.000 | 0.000 |
| ENSMUSG00000041220 | *Elovl6* | -3.663 | 0.000 | 0.000 |
| ENSMUSG00000010358 | *Ifi35* | -3.776 | 0.000 | 0.000 |
| ENSMUSG00000043067 | *Dpy19l1* | -3.078 | 0.000 | 0.000 |
| ENSMUSG00000022237 | *Ankrd33b* | 3.065 | 0.000 | 0.000 |
| ENSMUSG00000063445 | *Nmral1* | -3.075 | 0.000 | 0.000 |
| ENSMUSG00000040483 | *Xaf1* | -6.843 | 0.000 | 0.000 |
| ENSMUSG00000078853 | *Igtp* | -8.187 | 0.000 | 0.000 |
| ENSMUSG00000024601 | *Isoc1* | -2.897 | 0.000 | 0.000 |
| ENSMUSG00000031349 | *Nsdhl* | -3.429 | 0.000 | 0.000 |
| ENSMUSG00000069874 | *Irgm2* | -6.483 | 0.000 | 0.000 |
| ENSMUSG00000027514 | *Zbp1* | -6.396 | 0.000 | 0.000 |
| ENSMUSG00000026604 | *Ptpn14* | 6.382 | 0.000 | 0.000 |
| ENSMUSG00000024338 | *Psmb8* | -4.481 | 0.000 | 0.000 |
| ENSMUSG00000037419 | *Endod1* | -3.518 | 0.000 | 0.000 |
| ENSMUSG00000027952 | *Pmvk* | -3.414 | 0.000 | 0.000 |
| ENSMUSG00000062901 | *Klhl24* | 3.039 | 0.000 | 0.000 |
| ENSMUSG00000018899 | *Irf1* | -3.848 | 0.000 | 0.000 |
| ENSMUSG00000025743 | *Sdc3* | -8.242 | 0.000 | 0.000 |
| ENSMUSG00000057346 | *Apol9a* | -7.473 | 0.000 | 0.000 |
| ENSMUSG00000026080 | *Chst10* | -3.507 | 0.000 | 0.000 |
| ENSMUSG00000030921 | *Trim30a* | -6.811 | 0.000 | 0.000 |
| ENSMUSG00000020399 | *Havcr2* | 3.806 | 0.000 | 0.000 |
| ENSMUSG00000020156 | *Mum1* | -2.839 | 0.000 | 0.000 |
| ENSMUSG00000039853 | *Trim14* | -3.127 | 0.000 | 0.000 |
| ENSMUSG00000022014 | *Epsti1* | -6.350 | 0.000 | 0.000 |
| ENSMUSG00000026104 | *Stat1* | -4.464 | 0.000 | 0.000 |
| ENSMUSG00000078920 | *Ifi47* | -5.954 | 0.000 | 0.000 |
| ENSMUSG00000043421 | *Hilpda* | 3.491 | 0.000 | 0.000 |
| ENSMUSG00000062488 | *Ifit3b* | -10.298 | 0.000 | 0.000 |
| ENSMUSG00000026946 | *Nmi* | -3.772 | 0.000 | 0.000 |
| ENSMUSG00000052336 | *Cx3cr1* | -4.280 | 0.000 | 0.000 |
| ENSMUSG00000028268 | *Gbp3* | -6.611 | 0.000 | 0.000 |
| ENSMUSG00000068246 | *Apol9b* | -7.432 | 0.000 | 0.000 |
| ENSMUSG00000000386 | *Mx1* | -12.674 | 0.000 | 0.000 |
| ENSMUSG00000030966 | *Trim21* | -4.673 | 0.000 | 0.000 |
| ENSMUSG00000046733 | *Gprc5a* | 4.248 | 0.000 | 0.000 |
| ENSMUSG00000003545 | *Fosb* | 4.454 | 0.000 | 0.000 |
| ENSMUSG00000024677 | *Ms4a6b* | -5.681 | 0.000 | 0.000 |
| ENSMUSG00000031488 | *Rab11fip1* | 2.075 | 4.94065645841247e-322 | 4.3769275565076e-320 |
| ENSMUSG00000058454 | *Dhcr7* | -2.724 | 1.70905557792773e-316 | 1.46966259139594e-314 |
| ENSMUSG00000021273 | *Fdft1* | -2.318 | 1.28592945852643e-315 | 1.10176934192237e-313 |
| ENSMUSG00000085795 | *Zfp703* | 2.716 | 6.59942441437115e-315 | 5.63374863825596e-313 |
| ENSMUSG00000029298 | *Gbp9* | -6.963 | 4.70789753784615e-313 | 4.00444212313334e-311 |
| ENSMUSG00000068735 | *Trp53i11* | -4.196 | 8.86388948940718e-312 | 7.48520394436413e-310 |
| ENSMUSG00000073489 | *Ifi204* | -3.482 | 5.91051549543441e-311 | 0.000 |
| ENSMUSG00000096727 | *Psmb9* | -6.860 | 0.000 | 0.000 |
| ENSMUSG00000024339 | *Tap2* | -3.269 | 0.000 | 0.000 |
| ENSMUSG00000024378 | *Stard4* | -2.621 | 0.000 | 0.000 |
| ENSMUSG00000029366 | *Dck* | -2.280 | 0.000 | 0.000 |
| ENSMUSG00000044583 | *Tlr7* | -2.055 | 0.000 | 0.000 |
| ENSMUSG00000033863 | *Klf9* | 2.138 | 0.000 | 0.000 |
| ENSMUSG00000033159 | *Cnppd1* | 2.009 | 0.000 | 0.000 |
| ENSMUSG00000045005 | *Fzd5* | 2.728 | 0.000 | 0.000 |
| ENSMUSG00000006930 | *Hap1* | -3.408 | 0.000 | 0.000 |
| ENSMUSG00000006517 | *Mvd* | -2.281 | 0.000 | 0.000 |
| ENSMUSG00000020923 | *Ubtf* | -2.026 | 0.000 | 0.000 |
| ENSMUSG00000066861 | *Oas1g* | -2.617 | 0.000 | 0.000 |
| ENSMUSG00000052085 | *Dock8* | -2.304 | 0.000 | 0.000 |
| ENSMUSG00000002325 | *Irf9* | -2.327 | 0.000 | 0.000 |
| ENSMUSG00000028599 | *Tnfrsf1b* | 2.026 | 0.000 | 0.000 |
| ENSMUSG00000032698 | *Lmo2* | -2.397 | 0.000 | 0.000 |
| ENSMUSG00000028527 | *Ak4* | 3.479 | 0.000 | 0.000 |
| ENSMUSG00000026773 | *Pfkfb3* | 2.062 | 0.000 | 0.000 |
| ENSMUSG00000000682 | *Cd52* | -2.685 | 0.000 | 0.000 |
| ENSMUSG00000071350 | *Setdb2* | -2.522 | 0.000 | 0.000 |
| ENSMUSG00000038067 | *Csf3* | 5.927 | 0.000 | 0.000 |
| ENSMUSG00000023905 | *Tnfrsf12a* | 2.789 | 0.000 | 0.000 |
| ENSMUSG00000069892 | *9930111J21Rik2* | -3.514 | 0.000 | 0.000 |
| ENSMUSG00000036292 | *Gramd1c* | 4.487 | 0.000 | 0.000 |
| ENSMUSG00000027715 | *Ccna2* | -2.285 | 0.000 | 0.000 |
| ENSMUSG00000056698 | *Elmod3* | 2.959 | 0.000 | 0.000 |
| ENSMUSG00000030802 | *Bckdk* | -2.110 | 0.000 | 0.000 |
| ENSMUSG00000034765 | *Dusp5* | 2.200 | 0.000 | 0.000 |
| ENSMUSG00000035299 | *Mid1* | 3.093 | 0.000 | 0.000 |
| ENSMUSG00000042082 | *Arsb* | -2.358 | 0.000 | 0.000 |
| ENSMUSG00000025915 | *Sgk3* | -2.484 | 0.000 | 0.000 |
| ENSMUSG00000039997 | *Ifi203* | -2.113 | 0.000 | 0.000 |
| ENSMUSG00000024330 | *Col11a2* | 2.539 | 0.000 | 0.000 |
| ENSMUSG00000037997 | *Parp11* | -2.471 | 0.000 | 0.000 |
| ENSMUSG00000056091 | *St3gal5* | -2.526 | 0.000 | 0.000 |
| ENSMUSG00000041308 | *Sntb2* | 2.423 | 0.000 | 0.000 |
| ENSMUSG00000079017 | *Ifi27l2a* | -5.677 | 0.000 | 0.000 |
| ENSMUSG00000048779 | *P2ry6* | -5.681 | 0.000 | 0.000 |
| ENSMUSG00000027339 | *Rassf2* | -2.748 | 0.000 | 0.000 |
| ENSMUSG00000029798 | *Herc6* | -4.289 | 0.000 | 0.000 |
| ENSMUSG00000032410 | *Xrn1* | -2.153 | 0.000 | 0.000 |
| ENSMUSG00000026826 | *Nr4a2* | 3.022 | 0.000 | 0.000 |
| ENSMUSG00000022389 | *Tef* | 2.784 | 0.000 | 0.000 |
| ENSMUSG00000031378 | *Abcd1* | -2.733 | 0.000 | 0.000 |
| ENSMUSG00000024013 | *Fgd2* | -4.661 | 0.000 | 0.000 |
| ENSMUSG00000090877 | *Hspa1b* | 3.550 | 0.000 | 0.000 |
| ENSMUSG00000020897 | *Aurkb* | -2.190 | 0.000 | 0.000 |
| ENSMUSG00000028702 | *Rad54l* | -2.346 | 0.000 | 0.000 |
| ENSMUSG00000030102 | *Itpr1* | -2.028 | 0.000 | 0.000 |
| ENSMUSG00000027605 | *Acss2* | -3.218 | 0.000 | 0.000 |
| ENSMUSG00000073491 | *Ifi213* | -3.841 | 0.000 | 0.000 |
| ENSMUSG00000041939 | *Mvk* | -2.707 | 0.000 | 0.000 |
| ENSMUSG00000018819 | *Lsp1* | -2.544 | 0.000 | 0.000 |
| ENSMUSG00000050824 | *Sstr5* | 5.308 | 0.000 | 0.000 |
| ENSMUSG00000027848 | *Olfml3* | 5.025 | 0.000 | 0.000 |
| ENSMUSG00000037816 | *Fbxw17* | -2.792 | 0.000 | 0.000 |
| ENSMUSG00000019806 | *Aig1* | 2.001 | 0.000 | 0.000 |
| ENSMUSG00000025044 | *Msr1* | -2.812 | 0.000 | 0.000 |
| ENSMUSG00000028214 | *Gem* | 4.731 | 0.000 | 0.000 |
| ENSMUSG00000054293 | *P2ry10b* | 2.082 | 0.000 | 0.000 |
| ENSMUSG00000043263 | *Ifi209* | -5.110 | 0.000 | 0.000 |
| ENSMUSG00000000730 | *Dnmt3l* | 2.727 | 0.000 | 0.000 |
| ENSMUSG00000027322 | *Siglec1* | -7.931 | 0.000 | 0.000 |
| ENSMUSG00000031442 | *Mcf2l* | -2.724 | 0.000 | 0.000 |
| ENSMUSG00000056116 | *H2-T22* | -3.127 | 0.000 | 0.000 |
| ENSMUSG00000057143 | *Trim12c* | -4.497 | 0.000 | 0.000 |
| ENSMUSG00000058099 | *Nfam1* | -2.893 | 0.000 | 0.000 |
| ENSMUSG00000020284 | *1810043G02Rik* | 2.495 | 0.000 | 0.000 |
| ENSMUSG00000028972 | *Car6* | 2.357 | 0.000 | 0.000 |
| ENSMUSG00000028238 | *Atp6v0d2* | 2.957 | 0.000 | 0.000 |
| ENSMUSG00000052821 | *Cysltr1* | -5.123 | 0.000 | 0.000 |
| ENSMUSG00000004791 | *Pgf* | 5.765 | 0.000 | 0.000 |
| ENSMUSG00000025372 | *Baiap2* | 2.372 | 0.000 | 0.000 |
| ENSMUSG00000019947 | *Arid5b* | 2.010 | 0.000 | 0.000 |
| ENSMUSG00000051212 | *Gpr183* | -2.181 | 0.000 | 0.000 |
| ENSMUSG00000052688 | *Rab7b* | -2.613 | 0.000 | 0.000 |
| ENSMUSG00000050931 | *Sgms2* | 4.141 | 0.000 | 0.000 |
| ENSMUSG00000038295 | *Atg9b* | -2.742 | 0.000 | 0.000 |
| ENSMUSG00000053040 | *Aph1c* | 3.174 | 0.000 | 0.000 |
| ENSMUSG00000039217 | *Il18* | -4.527 | 0.000 | 0.000 |
| ENSMUSG00000037868 | *Egr2* | 2.908 | 0.000 | 0.000 |
| ENSMUSG00000026479 | *Lamc2* | 4.629 | 0.000 | 0.000 |
| ENSMUSG00000058163 | *Gm5431* | -7.798 | 0.000 | 0.000 |
| ENSMUSG00000091649 | *Phf11b* | -5.439 | 0.000 | 0.000 |
| ENSMUSG00000039770 | *Ypel5* | 2.631 | 0.000 | 0.000 |
| ENSMUSG00000038508 | *Gdf15* | 3.277 | 0.000 | 0.000 |
| ENSMUSG00000041936 | *Agrn* | -2.745 | 0.000 | 0.000 |
| ENSMUSG00000002602 | *Axl* | -6.006 | 0.000 | 0.000 |
| ENSMUSG00000040276 | *Pacsin1* | -3.398 | 0.000 | 0.000 |
| ENSMUSG00000031639 | *Tlr3* | -7.830 | 0.000 | 0.000 |
| ENSMUSG00000026657 | *Frmd4a* | -2.899 | 0.000 | 0.000 |
| ENSMUSG00000021775 | *Nr1d2* | 2.225 | 0.000 | 0.000 |
| ENSMUSG00000029385 | *Ccng2* | 2.824 | 0.000 | 0.000 |
| ENSMUSG00000030223 | *Ptpro* | -2.456 | 0.000 | 0.000 |
| ENSMUSG00000044468 | *Tent5c* | 4.433 | 0.000 | 0.000 |
| ENSMUSG00000055184 | *Fam72a* | -2.423 | 0.000 | 0.000 |
| ENSMUSG00000040264 | *Gbp2b* | -7.049 | 0.000 | 0.000 |
| ENSMUSG00000039531 | *Zup1* | -2.324 | 0.000 | 0.000 |
| ENSMUSG00000021367 | *Edn1* | 6.089 | 0.000 | 0.000 |
| ENSMUSG00000031493 | *Ggn* | 2.961 | 0.000 | 0.000 |
| ENSMUSG00000039699 | *Batf2* | -10.882 | 0.000 | 0.000 |
| ENSMUSG00000055612 | *Cdca7* | -2.311 | 0.000 | 0.000 |
| ENSMUSG00000026069 | *Il1rl1* | -2.299 | 0.000 | 0.000 |
| ENSMUSG00000020838 | *Slc6a4* | -3.319 | 0.000 | 0.000 |
| ENSMUSG00000027171 | *Prrg4* | 2.050 | 0.000 | 0.000 |
| ENSMUSG00000074063 | *Osgin1* | 2.342 | 0.000 | 0.000 |
| ENSMUSG00000028341 | *Nr4a3* | 6.235 | 0.000 | 0.000 |
| ENSMUSG00000037921 | *Ddx60* | -6.923 | 0.000 | 0.000 |
| ENSMUSG00000048249 | *Crebrf* | 2.183 | 0.000 | 0.000 |
| ENSMUSG00000021591 | *Glrx* | -2.134 | 0.000 | 0.000 |
| ENSMUSG00000079419 | *Ms4a6c* | -6.858 | 0.000 | 0.000 |
| ENSMUSG00000079197 | *Psme2* | -2.705 | 0.000 | 0.000 |
| ENSMUSG00000041774 | *Ydjc* | -2.139 | 0.000 | 0.000 |
| ENSMUSG00000032066 | *Bco2* | -3.424 | 0.000 | 0.000 |
| ENSMUSG00000024036 | *Slc37a1* | -2.040 | 0.000 | 0.000 |
| ENSMUSG00000044066 | *Cep68* | -2.541 | 0.000 | 0.000 |
| ENSMUSG00000025742 | *Prps2* | -2.298 | 0.000 | 0.000 |
| ENSMUSG00000004698 | *Hdac9* | -2.759 | 0.000 | 0.000 |
| ENSMUSG00000046329 | *Slc25a23* | -3.192 | 0.000 | 0.000 |
| ENSMUSG00000090215 | *Trim34b* | -7.680 | 0.000 | 0.000 |
| ENSMUSG00000028270 | *Gbp2* | -7.284 | 0.000 | 0.000 |
| ENSMUSG00000040797 | *Iqsec3* | 6.539 | 0.000 | 0.000 |
| ENSMUSG00000031504 | *Rab20* | 2.519 | 0.000 | 0.000 |
| ENSMUSG00000000056 | *Narf* | 2.037 | 0.000 | 0.000 |
| ENSMUSG00000033022 | *Cdo1* | 3.540 | 0.000 | 0.000 |
| ENSMUSG00000062075 | *Lmnb2* | -3.177 | 0.000 | 0.000 |
| ENSMUSG00000022848 | *Dirc2* | 2.678 | 0.000 | 0.000 |
| ENSMUSG00000006445 | *Epha2* | 2.095 | 0.000 | 0.000 |
| ENSMUSG00000028655 | *Mfsd2a* | -2.767 | 0.000 | 0.000 |
| ENSMUSG00000025058 | *5430427O19Rik* | -2.470 | 0.000 | 0.000 |
| ENSMUSG00000002996 | *Hbp1* | 2.218 | 0.000 | 0.000 |
| ENSMUSG00000048388 | *Fam171b* | 2.522 | 0.000 | 0.000 |
| ENSMUSG00000032657 | *Fam189b* | 2.225 | 0.000 | 0.000 |
| ENSMUSG00000025036 | *Sfxn2* | -2.745 | 0.000 | 0.000 |
| ENSMUSG00000023034 | *Nr4a1* | 2.696 | 0.000 | 0.000 |
| ENSMUSG00000009585 | *Apobec3* | -2.056 | 0.000 | 0.000 |
| ENSMUSG00000021175 | *Cdca7l* | -3.148 | 0.000 | 0.000 |
| ENSMUSG00000038217 | *Tlcd2* | 3.096 | 0.000 | 0.000 |
| ENSMUSG00000025076 | *Casp7* | -3.026 | 0.000 | 0.000 |
| ENSMUSG00000020658 | *Efr3b* | -2.120 | 0.000 | 0.000 |
| ENSMUSG00000037239 | *Spred3* | 2.450 | 0.000 | 0.000 |
| ENSMUSG00000022602 | *Arc* | 2.554 | 0.000 | 0.000 |
| ENSMUSG00000027360 | *Hdc* | -3.293 | 0.000 | 0.000 |
| ENSMUSG00000022346 | *Myc* | 2.508 | 0.000 | 0.000 |
| ENSMUSG00000024827 | *Gldc* | 3.995 | 0.000 | 0.000 |
| ENSMUSG00000054676 | *1600014C10Rik* | -2.739 | 0.000 | 0.000 |
| ENSMUSG00000031562 | *Dctd* | -2.490 | 0.000 | 0.000 |
| ENSMUSG00000009905 | *Kdsr* | 2.181 | 0.000 | 0.000 |
| ENSMUSG00000013483 | *Card14* | 3.028 | 0.000 | 0.000 |
| ENSMUSG00000073409 | *H2-Q6* | -2.086 | 0.000 | 0.000 |
| ENSMUSG00000035206 | *Sppl2b* | -2.489 | 0.000 | 0.000 |
| ENSMUSG00000059743 | *Fdps* | -3.804 | 0.000 | 0.000 |
| ENSMUSG00000028575 | *Eqtn* | 3.418 | 0.000 | 0.000 |
| ENSMUSG00000029314 | *Gpat3* | 2.065 | 0.000 | 0.000 |
| ENSMUSG00000048806 | *Ifnb1* | -5.875 | 0.000 | 0.000 |
| ENSMUSG00000020653 | *Klf11* | 3.197 | 0.000 | 0.000 |
| ENSMUSG00000028064 | *Sema4a* | -2.485 | 0.000 | 0.000 |
| ENSMUSG00000029442 | *Wdr66* | 2.379 | 0.000 | 0.000 |
| ENSMUSG00000059089 | *Fcgr4* | -4.092 | 0.000 | 0.000 |
| ENSMUSG00000040828 | *Catsperd* | 3.125 | 0.000 | 0.000 |
| ENSMUSG00000041842 | *Fhdc1* | 2.484 | 0.000 | 0.000 |
| ENSMUSG00000023206 | *Il15ra* | -4.038 | 0.000 | 0.000 |
| ENSMUSG00000044701 | *Il27* | -5.141 | 0.000 | 0.000 |
| ENSMUSG00000026536 | *Ifi211* | -3.526 | 0.000 | 0.000 |
| ENSMUSG00000074649 | *BC029722* | -2.352 | 0.000 | 0.000 |
| ENSMUSG00000018849 | *Wwc1* | 3.414 | 0.000 | 0.000 |
| ENSMUSG00000006219 | *Fblim1* | 2.431 | 0.000 | 0.000 |
| ENSMUSG00000078153 | *Psme2b* | -2.230 | 0.000 | 0.000 |
| ENSMUSG00000020593 | *Lpin1* | -2.803 | 0.000 | 0.000 |
| ENSMUSG00000091144 | *Phf11c* | -2.530 | 0.000 | 0.000 |
| ENSMUSG00000020034 | *Tcp11l2* | 2.229 | 0.000 | 0.000 |
| ENSMUSG00000047749 | *Zc3hav1l* | 2.151 | 0.000 | 0.000 |
| ENSMUSG00000033033 | *Calhm2* | -2.603 | 0.000 | 0.000 |
| ENSMUSG00000041064 | *Pif1* | -2.072 | 0.000 | 0.000 |
| ENSMUSG00000039713 | *Plekhg5* | -3.291 | 0.000 | 0.000 |
| ENSMUSG00000069893 | *9930111J21Rik1* | -3.331 | 0.000 | 0.000 |
| ENSMUSG00000021384 | *Susd3* | -2.148 | 0.000 | 0.000 |
| ENSMUSG00000026205 | *Slc23a3* | 4.296 | 0.000 | 0.000 |
| ENSMUSG00000038167 | *Plekhg6* | 3.269 | 0.000 | 0.000 |
| ENSMUSG00000039304 | *Tnfsf10* | -6.820 | 0.000 | 0.000 |
| ENSMUSG00000026715 | *Serpinc1* | 3.738 | 0.000 | 0.000 |
| ENSMUSG00000031538 | *Plat* | 6.965 | 0.000 | 0.000 |
| ENSMUSG00000049871 | *Nlrc3* | 2.010 | 0.000 | 0.000 |
| ENSMUSG00000024589 | *Nedd4l* | 2.872 | 0.000 | 0.000 |
| ENSMUSG00000038224 | *Serpinf2* | 4.858 | 0.000 | 0.000 |
| ENSMUSG00000036353 | *P2ry12* | -4.602 | 0.000 | 0.000 |
| ENSMUSG00000032265 | *Tent5a* | -2.351 | 0.000 | 0.000 |
| ENSMUSG00000037428 | *Vgf* | 3.012 | 0.000 | 0.000 |
| ENSMUSG00000078349 | *AW011738* | -4.321 | 0.000 | 0.000 |
| ENSMUSG00000020334 | *Slc22a4* | 4.277 | 0.000 | 0.000 |
| ENSMUSG00000053846 | *Lipg* | -9.920 | 0.000 | 0.000 |
| ENSMUSG00000022441 | *Efcab6* | 6.238 | 0.000 | 0.000 |
| ENSMUSG00000019846 | *Lama4* | -3.447 | 0.000 | 0.000 |
| ENSMUSG00000062101 | *Zfp119b* | 2.329 | 0.000 | 0.000 |
| ENSMUSG00000038776 | *Ephx1* | 2.901 | 0.000 | 0.000 |
| ENSMUSG00000071068 | *Treml2* | -4.481 | 0.000 | 0.000 |
| ENSMUSG00000043154 | *Ppp2r3a* | 2.033 | 0.000 | 0.000 |
| ENSMUSG00000028128 | *F3* | 3.478 | 0.000 | 0.000 |
| ENSMUSG00000046295 | *Ankle1* | -3.387 | 0.000 | 0.000 |
| ENSMUSG00000051682 | *Treml4* | 2.733 | 0.000 | 0.000 |
| ENSMUSG00000001911 | *Nfix* | -2.438 | 0.000 | 0.000 |
| ENSMUSG00000067297 | *Ifit1bl2* | -5.960 | 0.000 | 0.000 |
| ENSMUSG00000029361 | *Nos1* | 3.387 | 0.000 | 0.000 |
| ENSMUSG00000028551 | *Cdkn2c* | -2.301 | 0.000 | 0.000 |
| ENSMUSG00000039783 | *Kmo* | -6.347 | 0.000 | 0.000 |
| ENSMUSG00000017493 | *Igfbp4* | -3.195 | 0.000 | 0.000 |
| ENSMUSG00000003882 | *Il7r* | 3.875 | 0.000 | 0.000 |
| ENSMUSG00000036249 | *Rbm43* | -2.237 | 0.000 | 0.000 |
| ENSMUSG00000023982 | *Guca1a* | -2.157 | 0.000 | 0.000 |
| ENSMUSG00000105504 | *Gbp5* | -9.861 | 0.000 | 0.000 |
| ENSMUSG00000079652 | *Fam71f2* | 4.288 | 0.000 | 0.000 |
| ENSMUSG00000071713 | *Csf2rb* | -2.292 | 0.000 | 0.000 |
| ENSMUSG00000063894 | *Zkscan8* | -2.471 | 0.000 | 0.000 |
| ENSMUSG00000025348 | *Itga7* | 2.070 | 0.000 | 0.000 |
| ENSMUSG00000040703 | *Cyp2s1* | -6.759 | 0.000 | 0.000 |
| ENSMUSG00000071547 | *Nt5dc2* | -2.105 | 0.000 | 0.000 |
| ENSMUSG00000059791 | *Nrm* | -2.292 | 0.000 | 0.000 |
| ENSMUSG00000023074 | *Mospd1* | 2.008 | 0.000 | 0.000 |
| ENSMUSG00000055994 | *Nod2* | -2.057 | 0.000 | 0.000 |
| ENSMUSG00000068101 | *Cenpm* | -2.198 | 0.000 | 0.000 |
| ENSMUSG00000043822 | *Adamtsl5* | 2.042 | 0.000 | 0.000 |
| ENSMUSG00000026955 | *Sapcd2* | -2.114 | 0.000 | 0.000 |
| ENSMUSG00000049823 | *Zbtb12* | -2.165 | 0.000 | 0.000 |
| ENSMUSG00000054640 | *Slc8a1* | -4.014 | 0.000 | 0.000 |
| ENSMUSG00000044165 | *Bcl2l15* | 3.382 | 0.000 | 0.000 |
| ENSMUSG00000041115 | *Iqsec2* | -2.025 | 0.000 | 0.000 |
| ENSMUSG00000060509 | *Xcr1* | 3.403 | 0.000 | 0.000 |
| ENSMUSG00000001166 | *Oas1c* | -4.986 | 0.000 | 0.000 |
| ENSMUSG00000055301 | *Adh7* | 2.188 | 0.000 | 0.000 |
| ENSMUSG00000006567 | *Atp7b* | 4.226 | 0.000 | 0.000 |
| ENSMUSG00000028167 | *Bdh2* | 3.806 | 0.000 | 0.000 |
| ENSMUSG00000079173 | *Zan* | 3.621 | 0.000 | 0.000 |
| ENSMUSG00000026365 | *Cfh* | -2.898 | 0.000 | 0.000 |
| ENSMUSG00000039741 | *Bahcc1* | -5.712 | 0.000 | 0.000 |
| ENSMUSG00000034488 | *Edil3* | 2.821 | 0.000 | 0.000 |
| ENSMUSG00000066456 | *Hmgn3* | -3.960 | 0.000 | 0.000 |
| ENSMUSG00000039154 | *Shd* | 3.072 | 0.000 | 0.000 |
| ENSMUSG00000024799 | *Tm7sf2* | -2.073 | 0.000 | 0.000 |
| ENSMUSG00000067786 | *Nnat* | 2.474 | 0.000 | 0.000 |
| ENSMUSG00000028758 | *Kif17* | -2.590 | 0.000 | 0.000 |
| ENSMUSG00000044716 | *Dok7* | 5.533 | 0.000 | 0.000 |
| ENSMUSG00000055945 | *Prr18* | 2.350 | 0.000 | 0.000 |
| ENSMUSG00000070385 | *Ampd1* | 3.055 | 0.000 | 0.000 |
| ENSMUSG00000032531 | *Amotl2* | -4.745 | 0.000 | 0.000 |
| ENSMUSG00000054072 | *Iigp1* | -10.281 | 0.000 | 0.000 |
| ENSMUSG00000023961 | *Enpp4* | -2.596 | 0.000 | 0.000 |
| ENSMUSG00000036030 | *Prtg* | -2.392 | 0.000 | 0.000 |
| ENSMUSG00000020808 | *Pimreg* | -2.363 | 0.000 | 0.000 |
| ENSMUSG00000006360 | *Crip1* | -2.863 | 0.000 | 0.000 |
| ENSMUSG00000042106 | *Inka1* | -2.970 | 0.000 | 0.000 |
| ENSMUSG00000041491 | *Cep78* | -2.089 | 0.000 | 0.000 |
| ENSMUSG00000054263 | *Lifr* | -4.098 | 0.000 | 0.000 |
| ENSMUSG00000032281 | *Acsbg1* | 2.182 | 0.000 | 0.000 |
| ENSMUSG00000037887 | *Dusp8* | 2.155 | 0.000 | 0.000 |
| ENSMUSG00000025010 | *Ccnj* | -2.024 | 0.000 | 0.000 |
| ENSMUSG00000028211 | *Trp53inp1* | 2.109 | 0.000 | 0.000 |
| ENSMUSG00000049709 | *Nlrp10* | -2.638 | 0.000 | 0.000 |
| ENSMUSG00000037260 | *Hgsnat* | -2.116 | 0.000 | 0.000 |
| ENSMUSG00000027002 | *Nckap1* | 2.026 | 0.000 | 0.000 |
| ENSMUSG00000006611 | *Hfe* | -3.216 | 0.000 | 0.000 |
| ENSMUSG00000027646 | *Src* | 3.244 | 0.000 | 0.000 |
| ENSMUSG00000034394 | *Lif* | 3.744 | 0.000 | 0.000 |
| ENSMUSG00000022330 | *Osr2* | 4.355 | 0.000 | 0.000 |
| ENSMUSG00000022548 | *Apod* | -9.398 | 0.000 | 0.000 |
| ENSMUSG00000029193 | *Cckar* | 4.104 | 0.000 | 0.000 |
| ENSMUSG00000035004 | *Igsf6* | -2.069 | 0.000 | 0.000 |
| ENSMUSG00000030494 | *Rhpn2* | 4.200 | 0.000 | 0.000 |
| ENSMUSG00000047604 | *Frat2* | 3.834 | 0.000 | 0.000 |
| ENSMUSG00000087141 | *Plcxd2* | 2.605 | 0.000 | 0.000 |
| ENSMUSG00000047139 | *Cd24a* | -3.588 | 0.000 | 0.000 |
| ENSMUSG00000049625 | *Tifab* | -3.644 | 0.000 | 0.000 |
| ENSMUSG00000056144 | *Trim34a* | -6.649 | 0.000 | 0.000 |
| ENSMUSG00000024675 | *Ms4a4c* | -9.235 | 0.000 | 0.000 |
| ENSMUSG00000039081 | *Zfp503* | 2.586 | 0.000 | 0.000 |
| ENSMUSG00000021876 | *Rnase4* | -2.333 | 0.000 | 0.000 |
| ENSMUSG00000030055 | *Rab43* | 2.196 | 0.000 | 0.000 |
| ENSMUSG00000030745 | *Il21r* | -2.616 | 0.000 | 0.000 |
| ENSMUSG00000043541 | *Casc1* | 2.727 | 0.000 | 0.000 |
| ENSMUSG00000057135 | *Scimp* | -2.325 | 0.000 | 0.000 |
| ENSMUSG00000025880 | *Smad7* | 2.731 | 0.000 | 0.000 |
| ENSMUSG00000010051 | *Hyal1* | 2.130 | 0.000 | 0.000 |
| ENSMUSG00000034460 | *Six4* | 2.689 | 0.000 | 0.000 |
| ENSMUSG00000062007 | *Hsh2d* | -2.439 | 0.000 | 0.000 |
| ENSMUSG00000021701 | *Plk2* | 2.142 | 0.000 | 0.000 |
| ENSMUSG00000029491 | *Pde6b* | 2.035 | 0.000 | 0.000 |
| ENSMUSG00000000881 | *Dlg3* | -2.561 | 0.000 | 0.000 |
| ENSMUSG00000038037 | *Socs1* | -2.085 | 0.000 | 0.000 |
| ENSMUSG00000026832 | *Cytip* | -4.103 | 0.000 | 0.000 |
| ENSMUSG00000050592 | *Fam78a* | -2.129 | 0.000 | 0.000 |
| ENSMUSG00000034586 | *Hid1* | 2.049 | 0.000 | 0.000 |
| ENSMUSG00000040722 | *Scamp5* | -2.154 | 0.000 | 0.000 |
| ENSMUSG00000044244 | *Il20rb* | 2.218 | 0.000 | 0.000 |
| ENSMUSG00000032238 | *Rora* | 3.546 | 0.000 | 0.000 |
| ENSMUSG00000006386 | *Tek* | 4.852 | 0.000 | 0.000 |
| ENSMUSG00000025380 | *Fscn2* | 3.382 | 0.000 | 0.000 |
| ENSMUSG00000043782 | *Bicdl2* | 3.753 | 0.000 | 0.000 |
| ENSMUSG00000022540 | *Rogdi* | -2.058 | 0.000 | 0.000 |
| ENSMUSG00000012123 | *Crybg2* | -2.335 | 0.000 | 0.000 |
| ENSMUSG00000066363 | *Serpina3f* | -7.643 | 0.000 | 0.000 |
| ENSMUSG00000030137 | *Tuba8* | -5.598 | 0.000 | 0.000 |
| ENSMUSG00000030376 | *Slc8a2* | 3.869 | 0.000 | 0.000 |
| ENSMUSG00000021262 | *Evl* | -5.908 | 0.000 | 0.000 |
| ENSMUSG00000070501 | *Ifi214* | -5.161 | 0.000 | 0.000 |
| ENSMUSG00000028621 | *Cyb5rl* | -2.163 | 0.000 | 0.000 |
| ENSMUSG00000028102 | *Pex11b* | -2.072 | 0.000 | 0.000 |
| ENSMUSG00000030431 | *Tmem238* | -2.141 | 0.000 | 0.000 |
| ENSMUSG00000043740 | *B430306N03Rik* | -3.689 | 0.000 | 0.000 |
| ENSMUSG00000035829 | *Ppp1r26* | -2.561 | 0.000 | 0.000 |
| ENSMUSG00000026435 | *Slc45a3* | 2.008 | 0.000 | 0.000 |
| ENSMUSG00000046152 | *Fut10* | -3.673 | 0.000 | 0.000 |
| ENSMUSG00000046031 | *Calhm6* | -4.210 | 0.000 | 0.000 |
| ENSMUSG00000036362 | *P2ry13* | -5.457 | 0.000 | 0.000 |
| ENSMUSG00000001930 | *Vwf* | 4.452 | 0.000 | 0.000 |
| ENSMUSG00000030256 | *Bhlhe41* | 2.629 | 0.000 | 0.000 |
| ENSMUSG00000023909 | *Paqr4* | -3.032 | 0.000 | 0.000 |
| ENSMUSG00000037820 | *Tgm2* | 6.882 | 0.000 | 0.000 |
| ENSMUSG00000015850 | *Adamtsl4* | 2.286 | 0.000 | 0.000 |
| ENSMUSG00000026764 | *Kif5c* | -3.989 | 0.000 | 0.000 |
| ENSMUSG00000040205 | *Cuzd1* | 3.989 | 0.000 | 0.000 |
| ENSMUSG00000054594 | *Oscar* | 4.130 | 0.000 | 0.000 |
| ENSMUSG00000110218 | *Gm20219* | 2.051 | 0.000 | 0.000 |
| ENSMUSG00000046688 | *Tifa* | -2.130 | 0.000 | 0.000 |
| ENSMUSG00000078921 | *Tgtp2* | -8.758 | 0.000 | 0.000 |
| ENSMUSG00000024887 | *Asah2* | -2.335 | 0.000 | 0.000 |
| ENSMUSG00000036106 | *Prr5* | -2.111 | 0.000 | 0.000 |
| ENSMUSG00000013584 | *Aldh1a2* | -6.394 | 0.000 | 0.000 |
| ENSMUSG00000037583 | *Nr0b2* | -3.302 | 0.000 | 0.000 |
| ENSMUSG00000042675 | *Ypel3* | 2.686 | 0.000 | 0.000 |
| ENSMUSG00000090698 | *Apold1* | 2.919 | 0.000 | 0.000 |
| ENSMUSG00000026433 | *Rab29* | -2.105 | 0.000 | 0.000 |
| ENSMUSG00000031955 | *Bcar1* | 3.024 | 0.000 | 0.000 |
| ENSMUSG00000038700 | *Hoxb5* | -2.527 | 0.000 | 0.000 |
| ENSMUSG00000041481 | *Serpina3g* | -6.813 | 0.000 | 0.000 |
| ENSMUSG00000079339 | *Ifit1bl1* | -5.582 | 0.000 | 0.000 |
| ENSMUSG00000049038 | *Mterf2* | 2.457 | 0.000 | 0.000 |
| ENSMUSG00000078616 | *Trim30c* | -8.920 | 0.000 | 0.000 |
| ENSMUSG00000033717 | *Adra2a* | 3.604 | 0.000 | 0.000 |
| ENSMUSG00000028111 | *Ctsk* | 2.716 | 0.000 | 0.000 |
| ENSMUSG00000068740 | *Celsr2* | 2.660 | 0.000 | 0.000 |
| ENSMUSG00000044674 | *Fzd1* | 8.563 | 0.000 | 0.000 |
| ENSMUSG00000025104 | *Hdgfl3* | -2.281 | 0.000 | 0.000 |
| ENSMUSG00000058470 | *Gm8369* | -8.513 | 0.000 | 0.000 |
| ENSMUSG00000020080 | *Hkdc1* | 6.917 | 0.000 | 0.000 |
| ENSMUSG00000109293 | *Dcst2* | 2.700 | 0.000 | 0.000 |
| ENSMUSG00000020183 | *Cpm* | 2.943 | 0.000 | 0.000 |
| ENSMUSG00000036882 | *Arhgap33* | -2.307 | 0.000 | 0.000 |
| ENSMUSG00000105053 | *Gm43064* | 8.634 | 0.000 | 0.000 |
| ENSMUSG00000087598 | *Zfp111* | -2.311 | 0.000 | 0.000 |
| ENSMUSG00000054517 | *Trim65* | -2.369 | 0.000 | 0.000 |
| ENSMUSG00000047473 | *Zfp30* | 2.590 | 0.000 | 0.000 |
| ENSMUSG00000045502 | *Hcar2* | -2.817 | 0.000 | 0.000 |
| ENSMUSG00000022912 | *Pros1* | -2.391 | 0.000 | 0.000 |
| ENSMUSG00000027602 | *Map1lc3a* | 2.033 | 0.000 | 0.000 |
| ENSMUSG00000024354 | *Slc23a1* | 2.965 | 0.000 | 0.000 |
| ENSMUSG00000001946 | *Esam* | 2.710 | 0.000 | 0.000 |
| ENSMUSG00000061531 | *Tmem236* | 2.305 | 0.000 | 0.000 |
| ENSMUSG00000032584 | *Mst1r* | 8.455 | 0.000 | 0.000 |
| ENSMUSG00000063594 | *Gng8* | 3.508 | 0.000 | 0.000 |
| ENSMUSG00000054206 | *Gzmm* | 2.439 | 0.000 | 0.000 |
| ENSMUSG00000037991 | *Rmi2* | -2.660 | 0.000 | 0.000 |
| ENSMUSG00000041592 | *Sdk2* | 5.855 | 0.000 | 0.000 |
| ENSMUSG00000027938 | *Creb3l4* | 2.090 | 0.000 | 0.000 |
| ENSMUSG00000016756 | *Cmah* | -4.510 | 0.000 | 0.000 |
| ENSMUSG00000021125 | *Arg2* | 2.271 | 0.000 | 0.000 |
| ENSMUSG00000071112 | *Spx* | 2.576 | 0.000 | 0.000 |
| ENSMUSG00000034452 | *Slc24a1* | 3.430 | 0.000 | 0.000 |
| ENSMUSG00000048852 | *Gm12185* | -5.208 | 0.000 | 0.000 |
| ENSMUSG00000004885 | *Crabp2* | 2.843 | 0.000 | 0.000 |
| ENSMUSG00000036091 | *Hyal3* | 2.133 | 0.000 | 0.000 |
| ENSMUSG00000034990 | *Otoa* | 3.595 | 0.000 | 0.000 |
| ENSMUSG00000037348 | *Paqr7* | -2.311 | 0.000 | 0.000 |
| ENSMUSG00000032482 | *Cspg5* | 2.842 | 0.000 | 0.000 |
| ENSMUSG00000023032 | *Slc4a8* | 2.029 | 0.000 | 0.000 |
| ENSMUSG00000020620 | *Abca8b* | 3.692 | 0.000 | 0.000 |
| ENSMUSG00000055866 | *Per2* | 2.514 | 0.000 | 0.000 |
| ENSMUSG00000015812 | *Gnrh1* | 2.242 | 0.000 | 0.000 |
| ENSMUSG00000033446 | *Lpar6* | -2.072 | 0.000 | 0.000 |
| ENSMUSG00000030088 | *Aldh1l1* | -5.574 | 0.000 | 0.000 |
| ENSMUSG00000015243 | *Abca1* | -2.919 | 0.000 | 0.000 |
| ENSMUSG00000086564 | *Cd101* | -4.829 | 0.000 | 0.000 |
| ENSMUSG00000003379 | *Cd79a* | 3.590 | 0.000 | 0.000 |
| ENSMUSG00000040428 | *Plekha4* | -2.185 | 0.000 | 0.000 |
| ENSMUSG00000040187 | *Arntl2* | 2.785 | 0.000 | 0.000 |
| ENSMUSG00000056648 | *Hoxb8* | -3.010 | 0.000 | 0.000 |
| ENSMUSG00000001020 | *S100a4* | -2.233 | 0.000 | 0.000 |
| ENSMUSG00000040904 | *Gm21988* | 3.541 | 0.000 | 0.000 |
| ENSMUSG00000032523 | *Hhatl* | 2.444 | 0.000 | 0.000 |
| ENSMUSG00000020892 | *Aloxe3* | 4.224 | 0.000 | 0.000 |
| ENSMUSG00000044566 | *Cage1* | 2.346 | 0.000 | 0.000 |
| ENSMUSG00000038400 | *Pmepa1* | 2.143 | 0.000 | 0.000 |
| ENSMUSG00000024019 | *Cmtr1* | -2.194 | 0.000 | 0.000 |
| ENSMUSG00000070904 | *Ifna4* | -8.165 | 0.000 | 0.000 |
| ENSMUSG00000050503 | *Fbxl22* | 2.010 | 0.000 | 0.000 |
| ENSMUSG00000025804 | *Ccr1* | 2.638 | 0.000 | 0.000 |
| ENSMUSG00000019326 | *Aoc3* | 2.507 | 0.000 | 0.000 |
| ENSMUSG00000074001 | *Klhl40* | 3.691 | 0.000 | 0.000 |
| ENSMUSG00000057948 | *Unc13d* | 2.524 | 0.000 | 0.000 |
| ENSMUSG00000026921 | *Egfl7* | -2.728 | 0.000 | 0.000 |
| ENSMUSG00000036244 | *Tbc1d21* | -2.239 | 0.000 | 0.000 |
| ENSMUSG00000028015 | *Ctso* | -2.646 | 0.000 | 0.000 |
| ENSMUSG00000105867 | *Gm42517* | 2.751 | 0.000 | 0.000 |
| ENSMUSG00000073555 | *Gm4951* | -8.014 | 0.000 | 0.000 |
| ENSMUSG00000046971 | *Pla2g4f* | 3.962 | 0.000 | 0.000 |
| ENSMUSG00000005824 | *Tnfsf14* | 2.536 | 0.000 | 0.000 |
| ENSMUSG00000052563 | *D930048N14Rik* | -2.503 | 0.000 | 0.000 |
| ENSMUSG00000072115 | *Ang* | -3.400 | 0.000 | 0.000 |
| ENSMUSG00000080316 | *Spaca6* | 2.172 | 0.000 | 0.000 |
| ENSMUSG00000022197 | *Pdzd2* | 3.300 | 0.000 | 0.000 |
| ENSMUSG00000097129 | *4930507D05Rik* | 3.446 | 0.000 | 0.000 |
| ENSMUSG00000025154 | *Arhgap19* | -2.194 | 0.000 | 0.000 |
| ENSMUSG00000073910 | *Mob3b* | -2.565 | 0.000 | 0.000 |
| ENSMUSG00000025020 | *Slit1* | 3.634 | 0.000 | 0.000 |
| ENSMUSG00000029108 | *Pcdh7* | -3.398 | 0.000 | 0.000 |
| ENSMUSG00000017400 | *Stac2* | 2.304 | 0.000 | 0.000 |
| ENSMUSG00000047953 | *Gp5* | 2.687 | 0.000 | 0.000 |
| ENSMUSG00000006362 | *Cbfa2t3* | -2.248 | 0.000 | 0.000 |
| ENSMUSG00000044083 | *Efcab8* | 2.538 | 0.000 | 0.000 |
| ENSMUSG00000044033 | *Ccdc141* | 3.964 | 0.000 | 0.000 |
| ENSMUSG00000048473 | *Sult6b2* | 6.237 | 0.000 | 0.000 |
| ENSMUSG00000040328 | *Olfr56* | -5.472 | 0.000 | 0.000 |
| ENSMUSG00000055177 | *Cstl1* | 2.791 | 0.000 | 0.000 |
| ENSMUSG00000059562 | *Ccdc154* | 4.441 | 0.000 | 0.000 |
| ENSMUSG00000055809 | *Dnaaf3* | -2.187 | 0.000 | 0.000 |
| ENSMUSG00000029925 | *Tbxas1* | -3.739 | 0.000 | 0.000 |
| ENSMUSG00000035373 | *Ccl7* | 3.174 | 0.000 | 0.000 |
| ENSMUSG00000028751 | *Pla2g2e* | 2.231 | 0.000 | 0.000 |
| ENSMUSG00000027186 | *Elf5* | 5.466 | 0.000 | 0.000 |
| ENSMUSG00000049608 | *Gpr55* | -2.882 | 0.000 | 0.000 |
| ENSMUSG00000025141 | *Myadml2* | -3.730 | 0.000 | 0.000 |
| ENSMUSG00000091491 | *Vmn2r97* | -3.066 | 0.000 | 0.000 |
| ENSMUSG00000020234 | *4930404N11Rik* | -2.296 | 0.000 | 0.000 |
| ENSMUSG00000023903 | *Mmp25* | 4.630 | 0.000 | 0.000 |
| ENSMUSG00000037849 | *Ifi206* | -5.308 | 0.000 | 0.000 |
| ENSMUSG00000074738 | *Fndc10* | -3.300 | 0.000 | 0.000 |
| ENSMUSG00000040093 | *Bmf* | -2.101 | 0.000 | 0.000 |
| ENSMUSG00000047591 | *Mafa* | 3.920 | 0.000 | 0.000 |
| ENSMUSG00000039021 | *Ttc16* | 2.603 | 0.000 | 0.000 |
| ENSMUSG00000078763 | *Slfn1* | -5.665 | 0.000 | 0.000 |
| ENSMUSG00000024395 | *Lims2* | 2.147 | 0.000 | 0.000 |
| ENSMUSG00000048424 | *Ranbp3l* | -2.499 | 0.000 | 0.000 |
| ENSMUSG00000109771 | *Gm35315* | -2.116 | 0.000 | 0.000 |
| ENSMUSG00000089844 | *A530032D15Rik* | -3.328 | 0.000 | 0.000 |
| ENSMUSG00000032021 | *Crtam* | 3.601 | 0.000 | 0.000 |
| ENSMUSG00000025279 | *Dnase1l3* | -7.874 | 0.000 | 0.000 |
| ENSMUSG00000025202 | *Scd3* | -5.740 | 0.000 | 0.000 |
| ENSMUSG00000044860 | *Gm1123* | 3.206 | 0.000 | 0.000 |
| ENSMUSG00000072612 | *Gm10382* | 2.053 | 0.000 | 0.000 |
| ENSMUSG00000023094 | *Msrb2* | -2.236 | 0.000 | 0.000 |
| ENSMUSG00000114278 | *Gm49027* | 2.062 | 0.000 | 0.000 |
| ENSMUSG00000026173 | *Plcd4* | 2.262 | 0.000 | 0.000 |
| ENSMUSG00000059956 | *Serpinb12* | -3.005 | 0.000 | 0.000 |
| ENSMUSG00000040478 | *Prdm13* | -2.881 | 0.000 | 0.000 |
| ENSMUSG00000078620 | *Gm10964* | 7.628 | 0.000 | 0.000 |
| ENSMUSG00000037577 | *Ephx3* | 3.100 | 0.000 | 0.000 |
| ENSMUSG00000024391 | *Apom* | 2.025 | 0.000 | 0.000 |
| ENSMUSG00000053461 | *Hhipl2* | 2.182 | 0.000 | 0.000 |
| ENSMUSG00000020435 | *Osbp2* | 2.511 | 0.000 | 0.000 |
| ENSMUSG00000027505 | *Fam209* | 2.978 | 0.000 | 0.000 |
| ENSMUSG00000038305 | *Spats2l* | -5.910 | 0.000 | 0.000 |
| ENSMUSG00000049086 | *Bmyc* | -2.734 | 0.000 | 0.000 |
| ENSMUSG00000038526 | *Car14* | 2.289 | 0.000 | 0.000 |
| ENSMUSG00000042607 | *Asb4* | 2.048 | 0.000 | 0.000 |
| ENSMUSG00000044122 | *Proca1* | 2.604 | 0.000 | 0.000 |
| ENSMUSG00000048621 | *Gm6377* | 2.120 | 0.000 | 0.000 |
| ENSMUSG00000024175 | *Tekt4* | 7.608 | 0.000 | 0.000 |
| ENSMUSG00000001739 | *Cldn15* | 2.127 | 0.000 | 0.000 |
| ENSMUSG00000042745 | *Id1* | 2.924 | 0.000 | 0.000 |
| ENSMUSG00000028460 | *Sit1* | -3.203 | 0.000 | 0.000 |
| ENSMUSG00000050195 | *Scd4* | -4.286 | 0.000 | 0.000 |
| ENSMUSG00000031637 | *Lrp2bp* | 2.760 | 0.000 | 0.000 |
| ENSMUSG00000020447 | *Npc1l1* | 2.591 | 0.000 | 0.000 |
| ENSMUSG00000032110 | *Acrv1* | 3.087 | 0.000 | 0.000 |
| ENSMUSG00000032611 | *1700102P08Rik* | 2.238 | 0.000 | 0.000 |
| ENSMUSG00000032387 | *Rbpms2* | 2.524 | 0.000 | 0.000 |
| ENSMUSG00000046085 | *4931422A03Rik* | -3.043 | 0.000 | 0.000 |
| ENSMUSG00000030046 | *Bmp10* | -4.576 | 0.000 | 0.000 |
| ENSMUSG00000037705 | *Tecta* | 3.454 | 0.000 | 0.000 |
| ENSMUSG00000030747 | *Dgat2* | -2.050 | 0.000 | 0.000 |
| ENSMUSG00000027318 | *Adam33* | -4.269 | 0.000 | 0.000 |
| ENSMUSG00000015966 | *Il17rb* | 2.277 | 0.000 | 0.000 |
| ENSMUSG00000079415 | *Cntf* | 2.965 | 0.000 | 0.000 |
| ENSMUSG00000054582 | *Pabpc1l* | 2.340 | 0.000 | 0.000 |
| ENSMUSG00000039963 | *Ccdc40* | 2.045 | 0.000 | 0.000 |
| ENSMUSG00000043789 | *Vwce* | 2.623 | 0.000 | 0.000 |
| ENSMUSG00000044703 | *Phf11a* | -5.827 | 0.000 | 0.000 |
| ENSMUSG00000034813 | *Grip1* | 3.270 | 0.000 | 0.000 |
| ENSMUSG00000018381 | *Abi3* | -3.288 | 0.000 | 0.000 |
| ENSMUSG00000031896 | *Ctrl* | -3.040 | 0.000 | 0.000 |
| ENSMUSG00000053198 | *Prx* | 2.331 | 0.000 | 0.000 |
| ENSMUSG00000020340 | *Cyfip2* | 2.070 | 0.000 | 0.000 |
| ENSMUSG00000047003 | *Zfp41* | -2.037 | 0.000 | 0.000 |
| ENSMUSG00000046854 | *Pip5kl1* | 3.830 | 0.000 | 0.000 |
| ENSMUSG00000018543 | *1700001P01Rik* | 2.187 | 0.000 | 0.000 |
| ENSMUSG00000069718 | *Gm11563* | 5.739 | 0.000 | 0.000 |
| ENSMUSG00000019913 | *Sim1* | 5.800 | 0.000 | 0.000 |
| ENSMUSG00000074577 | *Ripor3* | 3.192 | 0.000 | 0.000 |
| ENSMUSG00000022176 | *Rem2* | 2.942 | 0.000 | 0.000 |
| ENSMUSG00000042784 | *Muc1* | 2.860 | 0.000 | 0.000 |
| ENSMUSG00000001943 | *Vsig2* | 3.778 | 0.000 | 0.000 |
| ENSMUSG00000028359 | *Orm3* | 4.193 | 0.000 | 0.000 |
| ENSMUSG00000079481 | *Nhsl2* | -2.800 | 0.000 | 0.000 |
| ENSMUSG00000028786 | *Tmem54* | 5.588 | 0.000 | 0.000 |
| ENSMUSG00000030157 | *Clec2d* | -7.834 | 0.000 | 0.000 |
| ENSMUSG00000041444 | *Arhgap32* | -5.982 | 0.000 | 0.000 |
| ENSMUSG00000042659 | *Arrdc4* | 3.456 | 0.000 | 0.000 |
| ENSMUSG00000014773 | *Dll1* | -2.395 | 0.000 | 0.000 |
| ENSMUSG00000070570 | *Slc17a7* | 3.016 | 0.000 | 0.000 |
| ENSMUSG00000032338 | *Hcn4* | 7.481 | 0.000 | 0.000 |
| ENSMUSG00000019987 | *Arg1* | 2.207 | 0.000 | 0.000 |
| ENSMUSG00000003410 | *Elavl3* | 3.351 | 0.000 | 0.000 |
| ENSMUSG00000018238 | *Gdf9* | 2.791 | 0.000 | 0.000 |
| ENSMUSG00000055172 | *C1ra* | -4.593 | 0.000 | 0.000 |
| ENSMUSG00000070331 | *Qrich2* | 3.075 | 0.000 | 0.000 |
| ENSMUSG00000003849 | *Nqo1* | 2.211 | 0.000 | 0.000 |
| ENSMUSG00000038094 | *Atp13a4* | 3.548 | 0.000 | 0.000 |
| ENSMUSG00000039814 | *Xkr5* | -4.149 | 0.000 | 0.000 |
| ENSMUSG00000017817 | *Jph2* | 3.515 | 0.000 | 0.000 |
| ENSMUSG00000056290 | *Ms4a4b* | -7.297 | 0.000 | 0.000 |
| ENSMUSG00000019301 | *Hsd17b1* | 2.439 | 0.000 | 0.000 |
| ENSMUSG00000042333 | *Tnfrsf14* | -5.594 | 0.000 | 0.000 |
| ENSMUSG00000031397 | *Tktl1* | 3.745 | 0.000 | 0.000 |
| ENSMUSG00000087408 | *Cers1* | 3.637 | 0.000 | 0.000 |
| ENSMUSG00000035355 | *Kcnh4* | -7.318 | 0.000 | 0.000 |
| ENSMUSG00000028497 | *Hacd4* | -2.932 | 0.000 | 0.000 |
| ENSMUSG00000063409 | *Lrrc43* | 4.364 | 0.000 | 0.000 |
| ENSMUSG00000071342 | *Lsmem1* | 3.479 | 0.000 | 0.000 |
| ENSMUSG00000078137 | *Ankrd63* | 2.105 | 0.000 | 0.000 |
| ENSMUSG00000030854 | *Ptpn5* | -2.394 | 0.000 | 0.000 |
| ENSMUSG00000062585 | *Cnr2* | -2.258 | 0.000 | 0.000 |
| ENSMUSG00000071256 | *Zfp213* | -2.892 | 0.000 | 0.000 |
| ENSMUSG00000045377 | *Tmem88* | 3.242 | 0.000 | 0.000 |
| ENSMUSG00000028845 | *Tekt2* | 2.500 | 0.000 | 0.000 |
| ENSMUSG00000039653 | *Baat* | 4.709 | 0.000 | 0.000 |
| ENSMUSG00000052013 | *Btla* | -4.449 | 0.000 | 0.000 |
| ENSMUSG00000022838 | *Eaf2* | 2.278 | 0.000 | 0.000 |
| ENSMUSG00000022518 | *4930562C15Rik* | 2.454 | 0.000 | 0.000 |
| ENSMUSG00000038422 | *Hdhd3* | -2.705 | 0.000 | 0.000 |
| ENSMUSG00000030156 | *Cd69* | -3.662 | 0.000 | 0.000 |
| ENSMUSG00000075566 | *Krtap4-6* | 5.367 | 0.000 | 0.000 |
| ENSMUSG00000037994 | *Slc9b2* | 3.777 | 0.000 | 0.000 |
| ENSMUSG00000032065 | *Tex12* | -3.959 | 0.000 | 0.000 |
| ENSMUSG00000001663 | *Gstt1* | -3.958 | 0.000 | 0.000 |
| ENSMUSG00000020178 | *Adora2a* | 3.240 | 0.000 | 0.000 |
| ENSMUSG00000021278 | *Amn* | 2.451 | 0.000 | 0.000 |
| ENSMUSG00000024131 | *Slc3a1* | 2.754 | 0.000 | 0.000 |
| ENSMUSG00000045777 | *Ifitm10* | 4.685 | 0.000 | 0.000 |
| ENSMUSG00000004371 | *Il11* | 2.977 | 0.000 | 0.000 |
| ENSMUSG00000028794 | *A3galt2* | 3.222 | 0.000 | 0.000 |
| ENSMUSG00000095276 | *Gfy* | 6.986 | 0.000 | 0.000 |
| ENSMUSG00000051497 | *Kcnj16* | -2.097 | 0.000 | 0.000 |
| ENSMUSG00000045382 | *Cxcr4* | 3.737 | 0.000 | 0.000 |
| ENSMUSG00000066677 | *Ifi208* | -2.622 | 0.000 | 0.000 |
| ENSMUSG00000042734 | *Ttc9* | 2.347 | 0.000 | 0.000 |
| ENSMUSG00000029597 | *Sds* | 2.113 | 0.000 | 0.000 |
| ENSMUSG00000048498 | *Cd300e* | -3.832 | 0.000 | 0.000 |
| ENSMUSG00000042678 | *Myo15* | 2.454 | 0.000 | 0.000 |
| ENSMUSG00000052749 | *Trim30b* | -5.191 | 0.000 | 0.000 |
| ENSMUSG00000070576 | *Mn1* | 5.227 | 0.000 | 0.000 |
| ENSMUSG00000037542 | *Aldh8a1* | 4.155 | 0.000 | 0.000 |
| ENSMUSG00000044103 | *Il1f9* | 5.312 | 0.000 | 0.000 |
| ENSMUSG00000030859 | *Fam24a* | 2.950 | 0.000 | 0.000 |
| ENSMUSG00000050473 | *Slc35d3* | 3.115 | 0.000 | 0.000 |
| ENSMUSG00000073795 | *6430531B16Rik* | -2.258 | 0.000 | 0.000 |
| ENSMUSG00000059022 | *Kcp* | 2.287 | 0.000 | 0.000 |
| ENSMUSG00000000686 | *Abhd15* | -2.702 | 0.000 | 0.000 |
| ENSMUSG00000031078 | *Cttn* | 2.599 | 0.000 | 0.000 |
| ENSMUSG00000032595 | *Cdhr4* | -3.811 | 0.000 | 0.000 |
| ENSMUSG00000091091 | *Kcnmb3* | 5.227 | 0.000 | 0.000 |
| ENSMUSG00000030858 | *Fam24b* | 2.435 | 0.000 | 0.000 |
| ENSMUSG00000042189 | *Tekt3* | 6.821 | 0.000 | 0.000 |
| ENSMUSG00000026751 | *Nr5a1* | 6.821 | 0.000 | 0.000 |
| ENSMUSG00000027204 | *Fbn1* | -6.851 | 0.000 | 0.000 |
| ENSMUSG00000107478 | *Gm45234* | 5.227 | 0.000 | 0.000 |
| ENSMUSG00000038859 | *Baiap2l1* | 2.230 | 0.000 | 0.000 |
| ENSMUSG00000030125 | *Lrrc23* | 5.137 | 0.000 | 0.000 |
| ENSMUSG00000026697 | *Myoc* | 2.937 | 0.000 | 0.000 |
| ENSMUSG00000090891 | *D6Ertd527e* | 3.755 | 0.000 | 0.000 |
| ENSMUSG00000091191 | *Gm17334* | -7.256 | 0.000 | 0.000 |
| ENSMUSG00000035852 | *Misp* | -5.045 | 0.000 | 0.000 |
| ENSMUSG00000039329 | *Tex19.1* | 4.528 | 0.000 | 0.000 |
| ENSMUSG00000055102 | *Zfp819* | 3.755 | 0.000 | 0.000 |
| ENSMUSG00000001027 | *Scn4a* | 2.700 | 0.000 | 0.000 |
| ENSMUSG00000022101 | *Fgf17* | 2.739 | 0.000 | 0.000 |
| ENSMUSG00000034059 | *Ypel4* | 4.611 | 0.000 | 0.000 |
| ENSMUSG00000070424 | *Art5* | 3.078 | 0.000 | 0.000 |
| ENSMUSG00000021223 | *Papln* | 3.261 | 0.000 | 0.000 |
| ENSMUSG00000051504 | *Siglech* | -2.456 | 0.000 | 0.000 |
| ENSMUSG00000046814 | *Gchfr* | -2.378 | 0.000 | 0.000 |
| ENSMUSG00000095366 | *Gm21860* | 3.260 | 0.000 | 0.000 |
| ENSMUSG00000079363 | *Gbp4* | -6.716 | 0.000 | 0.000 |
| ENSMUSG00000051435 | *Fhad1* | 2.114 | 0.000 | 0.000 |
| ENSMUSG00000010044 | *Zmynd10* | 2.571 | 0.000 | 0.000 |
| ENSMUSG00000079550 | *Mpp4* | 3.532 | 0.000 | 0.000 |
| ENSMUSG00000034833 | *Tespa1* | -3.568 | 0.000 | 0.000 |
| ENSMUSG00000033377 | *Palmd* | 4.104 | 0.000 | 0.000 |
| ENSMUSG00000020098 | *Pcbd1* | 2.891 | 0.000 | 0.000 |
| ENSMUSG00000094728 | *AC132444.2* | -2.007 | 0.000 | 0.000 |
| ENSMUSG00000091285 | *Gm17430* | 4.527 | 0.000 | 0.000 |
| ENSMUSG00000021200 | *Asb2* | -5.017 | 0.000 | 0.000 |
| ENSMUSG00000021880 | *Rnase6* | -6.717 | 0.000 | 0.000 |
| ENSMUSG00000056367 | *Actr3b* | -2.192 | 0.000 | 0.000 |
| ENSMUSG00000022528 | *Hes1* | 3.433 | 0.000 | 0.000 |
| ENSMUSG00000096606 | *Tpbgl* | 5.166 | 0.000 | 0.000 |
| ENSMUSG00000090236 | *Car15* | 2.458 | 0.000 | 0.000 |
| ENSMUSG00000035121 | *Neil2* | 2.128 | 0.000 | 0.000 |
| ENSMUSG00000078354 | *Ifna2* | -6.605 | 0.000 | 0.000 |
| ENSMUSG00000061540 | *Orm2* | 3.334 | 0.000 | 0.000 |
| ENSMUSG00000049044 | *Rapgef4* | 6.712 | 0.000 | 0.000 |
| ENSMUSG00000038209 | *Itln1* | -3.805 | 0.000 | 0.000 |
| ENSMUSG00000020738 | *Sumo2* | -2.778 | 0.000 | 0.000 |
| ENSMUSG00000059864 | *Olfr1393* | 3.079 | 0.000 | 0.000 |
| ENSMUSG00000078276 | *Gm14190* | 4.937 | 0.000 | 0.000 |
| ENSMUSG00000024371 | *C2* | -2.021 | 0.000 | 0.000 |
| ENSMUSG00000013353 | *4931406B18Rik* | 6.712 | 0.000 | 0.000 |
| ENSMUSG00000039765 | *Cc2d2a* | -3.270 | 0.000 | 0.000 |
| ENSMUSG00000031104 | *Rab33a* | 2.186 | 0.000 | 0.000 |
| ENSMUSG00000070388 | *Fbxo39* | -3.013 | 0.000 | 0.000 |
| ENSMUSG00000091060 | *Myocos* | 3.325 | 0.000 | 0.000 |
| ENSMUSG00000026357 | *Rgs18* | -6.606 | 0.000 | 0.000 |
| ENSMUSG00000002565 | *Scin* | -3.176 | 0.000 | 0.000 |
| ENSMUSG00000049904 | *Tmem17* | -2.649 | 0.000 | 0.000 |
| ENSMUSG00000098549 | *Gm5269* | 4.188 | 0.000 | 0.000 |
| ENSMUSG00000000385 | *Tmprss2* | -4.224 | 0.000 | 0.000 |
| ENSMUSG00000038011 | *Dnah10* | 2.162 | 0.000 | 0.000 |
| ENSMUSG00000026774 | *4931423N10Rik* | 3.534 | 0.000 | 0.000 |
| ENSMUSG00000028115 | *Bnipl* | 2.679 | 0.000 | 0.000 |
| ENSMUSG00000054889 | *Dsp* | -2.915 | 0.000 | 0.000 |
| ENSMUSG00000049670 | *Morn4* | 2.035 | 0.000 | 0.000 |
| ENSMUSG00000046447 | *Camk2n1* | 3.816 | 0.000 | 0.000 |
| ENSMUSG00000038065 | *Mturn* | 4.973 | 0.000 | 0.000 |
| ENSMUSG00000051804 | *Adam6b* | -4.225 | 0.000 | 0.000 |
| ENSMUSG00000040367 | *Lrrd1* | 2.548 | 0.000 | 0.000 |
| ENSMUSG00000090881 | *Gm6904* | -3.188 | 0.000 | 0.000 |
| ENSMUSG00000000093 | *Tbx2* | 3.877 | 0.000 | 0.000 |
| ENSMUSG00000052819 | *Best2* | 2.382 | 0.000 | 0.000 |
| ENSMUSG00000028642 | *Tmem269* | 2.361 | 0.000 | 0.000 |
| ENSMUSG00000085486 | *Gm11634* | 2.552 | 0.000 | 0.000 |
| ENSMUSG00000032056 | *Btg4* | 3.816 | 0.000 | 0.000 |
| ENSMUSG00000041323 | *Ak7* | 3.751 | 0.000 | 0.000 |
| ENSMUSG00000004187 | *Kifc2* | 2.381 | 0.000 | 0.000 |
| ENSMUSG00000056271 | *Lman1l* | 2.352 | 0.000 | 0.000 |
| ENSMUSG00000072720 | *Myo18b* | 4.188 | 0.000 | 0.000 |
| ENSMUSG00000030787 | *Lyve1* | 6.422 | 0.000 | 0.000 |
| ENSMUSG00000047959 | *Kcna3* | -6.441 | 0.000 | 0.000 |
| ENSMUSG00000045065 | *9930022D16Rik* | 4.291 | 0.000 | 0.000 |
| ENSMUSG00000096682 | *Ifna5* | -6.675 | 0.000 | 0.000 |
| ENSMUSG00000029138 | *4930548H24Rik* | 2.897 | 0.000 | 0.000 |
| ENSMUSG00000024678 | *Ms4a4d* | -4.740 | 0.000 | 0.000 |
| ENSMUSG00000022696 | *Sidt1* | 3.783 | 0.000 | 0.000 |
| ENSMUSG00000018906 | *P4ha2* | 2.314 | 0.000 | 0.000 |
| ENSMUSG00000022220 | *Adcy4* | 2.105 | 0.000 | 0.000 |
| ENSMUSG00000069581 | *Tspear* | 2.707 | 0.000 | 0.000 |
| ENSMUSG00000005320 | *Fgfr4* | 2.794 | 0.000 | 0.000 |
| ENSMUSG00000067158 | *Col4a4* | 2.067 | 0.000 | 0.000 |
| ENSMUSG00000090173 | *Fbxw10* | 3.474 | 0.000 | 0.000 |
| ENSMUSG00000050762 | *Prss27* | 4.786 | 0.000 | 0.000 |
| ENSMUSG00000032656 | *MARCHF3* | 2.422 | 0.000 | 0.000 |
| ENSMUSG00000038179 | *Slamf7* | 3.566 | 0.000 | 0.000 |
| ENSMUSG00000048655 | *Ccdc169* | 6.327 | 0.000 | 0.000 |
| ENSMUSG00000022619 | *Mapk8ip2* | 3.238 | 0.000 | 0.000 |
| ENSMUSG00000041544 | *Disp3* | 2.341 | 0.000 | 0.000 |
| ENSMUSG00000034881 | *Tbxa2r* | 2.872 | 0.000 | 0.000 |
| ENSMUSG00000001763 | *Tspan33* | -6.480 | 0.000 | 0.000 |
| ENSMUSG00000074037 | *Mc1r* | 3.442 | 0.000 | 0.000 |
| ENSMUSG00000056671 | *Prelid2* | 2.186 | 0.000 | 0.000 |
| ENSMUSG00000079497 | *Gm13420* | 3.647 | 0.000 | 0.000 |
| ENSMUSG00000068009 | *Bpifb6* | 4.662 | 0.000 | 0.000 |
| ENSMUSG00000032323 | *Cyp11a1* | 3.268 | 0.000 | 0.000 |
| ENSMUSG00000006342 | *Susd2* | 2.174 | 0.000 | 0.000 |
| ENSMUSG00000070979 | *Actl7a* | 2.841 | 0.000 | 0.000 |
| ENSMUSG00000075269 | *Bex6* | -6.352 | 0.000 | 0.000 |
| ENSMUSG00000025243 | *Slc6a20b* | 3.881 | 0.000 | 0.000 |
| ENSMUSG00000024793 | *Tnfrsf25* | -2.497 | 0.000 | 0.000 |
| ENSMUSG00000027670 | *Ocstamp* | -2.997 | 0.000 | 0.000 |
| ENSMUSG00000058728 | *Cd300c* | -3.006 | 0.000 | 0.000 |
| ENSMUSG00000027208 | *Fgf7* | 2.108 | 0.000 | 0.000 |
| ENSMUSG00000029205 | *Chrna9* | 4.745 | 0.000 | 0.000 |
| ENSMUSG00000056656 | *Apol8* | 2.359 | 0.000 | 0.000 |
| ENSMUSG00000023247 | *Guca2a* | 3.734 | 0.000 | 0.000 |
| ENSMUSG00000038276 | *Asic3* | 2.048 | 0.000 | 0.000 |
| ENSMUSG00000090206 | *Tepp* | 4.221 | 0.000 | 0.000 |
| ENSMUSG00000055322 | *Tns1* | 2.454 | 0.000 | 0.000 |
| ENSMUSG00000047242 | *Taf9b* | 2.813 | 0.000 | 0.000 |
| ENSMUSG00000013523 | *Bcas1* | -6.393 | 0.000 | 0.000 |
| ENSMUSG00000042035 | *Igsf3* | 2.403 | 0.000 | 0.000 |
| ENSMUSG00000009350 | *Mpo* | 4.619 | 0.000 | 0.000 |
| ENSMUSG00000033187 | *BC016579* | 4.865 | 0.000 | 0.000 |
| ENSMUSG00000039521 | *Foxp3* | 2.394 | 0.000 | 0.000 |
| ENSMUSG00000034987 | *Hrh2* | -6.207 | 0.000 | 0.000 |
| ENSMUSG00000027223 | *Mapk8ip1* | 2.404 | 0.000 | 0.000 |
| ENSMUSG00000045348 | *Nyap1* | 2.783 | 0.000 | 0.000 |
| ENSMUSG00000058620 | *Adra2b* | 3.646 | 0.000 | 0.000 |
| ENSMUSG00000000411 | *Tssk3* | 2.579 | 0.000 | 0.000 |
| ENSMUSG00000031932 | *Gpr83* | 6.225 | 0.000 | 0.000 |
| ENSMUSG00000074569 | *Gcnt7* | 2.548 | 0.000 | 0.000 |
| ENSMUSG00000053310 | *Nrgn* | 2.867 | 0.000 | 0.000 |
| ENSMUSG00000030324 | *Rho* | 3.921 | 0.000 | 0.000 |
| ENSMUSG00000061086 | *Myl4* | 6.172 | 0.000 | 0.000 |
| ENSMUSG00000002459 | *Rgs20* | 2.703 | 0.000 | 0.000 |
| ENSMUSG00000105096 | *Gbp10* | -4.063 | 0.000 | 0.000 |
| ENSMUSG00000003352 | *Cacnb3* | 2.658 | 0.000 | 0.000 |
| ENSMUSG00000039438 | *Ttc36* | 4.526 | 0.000 | 0.000 |
| ENSMUSG00000038932 | *Tcfl5* | 2.901 | 0.000 | 0.000 |
| ENSMUSG00000026149 | *Tm4sf20* | 2.868 | 0.000 | 0.000 |
| ENSMUSG00000078554 | *Fam229a* | 3.962 | 0.000 | 0.000 |
| ENSMUSG00000022044 | *Stmn4* | 2.817 | 0.000 | 0.000 |
| ENSMUSG00000054976 | *Nyap2* | 6.469 | 0.000 | 0.000 |
| ENSMUSG00000031302 | *Nlgn3* | 2.434 | 0.000 | 0.000 |
| ENSMUSG00000026211 | *Obsl1* | 4.572 | 0.000 | 0.000 |
| ENSMUSG00000061603 | *Akap6* | 2.655 | 0.000 | 0.000 |
| ENSMUSG00000029608 | *Rph3a* | 3.834 | 0.000 | 0.000 |
| ENSMUSG00000028427 | *Aqp7* | 2.403 | 0.000 | 0.000 |
| ENSMUSG00000094942 | *Gm3604* | -2.764 | 0.000 | 0.000 |
| ENSMUSG00000021573 | *Tppp* | 2.960 | 0.000 | 0.000 |
| ENSMUSG00000039911 | *Spsb1* | -2.187 | 0.000 | 0.000 |
| ENSMUSG00000061451 | *Tmem151a* | 2.066 | 0.000 | 0.000 |
| ENSMUSG00000010142 | *Tnfrsf13b* | -2.312 | 0.000 | 0.000 |
| ENSMUSG00000010601 | *Apol7a* | -6.045 | 0.000 | 0.000 |
| ENSMUSG00000053469 | *Tg* | 2.135 | 0.000 | 0.000 |
| ENSMUSG00000061878 | *Sphk1* | 2.802 | 0.000 | 0.000 |
| ENSMUSG00000042567 | *Nek10* | 2.044 | 0.000 | 0.000 |
| ENSMUSG00000071633 | *Gm4952* | 6.171 | 0.000 | 0.000 |
| ENSMUSG00000075256 | *Cerkl* | -2.308 | 0.000 | 0.000 |
| ENSMUSG00000079164 | *Tlr5* | -6.389 | 0.000 | 0.000 |
| ENSMUSG00000094796 | *BC147527* | -6.389 | 0.000 | 0.000 |
| ENSMUSG00000102037 | *Bcl2a1a* | -2.348 | 0.000 | 0.000 |
| ENSMUSG00000095845 | *Gm5741* | 2.327 | 0.000 | 0.000 |
| ENSMUSG00000031766 | *Slc12a3* | 4.223 | 0.000 | 0.000 |
| ENSMUSG00000029410 | *Ppef2* | 3.265 | 0.000 | 0.000 |
| ENSMUSG00000032268 | *Tmprss5* | 2.320 | 0.000 | 0.000 |
| ENSMUSG00000090685 | *Atp6v1fnb* | 2.836 | 0.000 | 0.000 |
| ENSMUSG00000035560 | *Wdr20rt* | 6.057 | 0.000 | 0.000 |
| ENSMUSG00000057666 | *Gapdh* | 2.147 | 0.000 | 0.000 |
| ENSMUSG00000032083 | *Apoa1* | 3.381 | 0.000 | 0.000 |
| ENSMUSG00000014030 | *Pax5* | -6.307 | 0.000 | 0.000 |
| ENSMUSG00000110622 | *Iqcn* | 2.376 | 0.000 | 0.000 |
| ENSMUSG00000030638 | *Sh3gl3* | 3.276 | 0.000 | 0.000 |
| ENSMUSG00000027398 | *Il1b* | -2.520 | 0.000 | 0.000 |
| ENSMUSG00000022836 | *Mylk* | -5.927 | 0.000 | 0.000 |
| ENSMUSG00000030653 | *Gm45837* | 2.941 | 0.000 | 0.000 |
| ENSMUSG00000091381 | *Vmn2r83* | 4.322 | 0.000 | 0.000 |
| ENSMUSG00000027474 | *Ccm2l* | -4.502 | 0.000 | 0.000 |
| ENSMUSG00000032332 | *Col12a1* | 2.199 | 0.000 | 0.000 |
| ENSMUSG00000092586 | *Ly6g6c* | 4.322 | 0.000 | 0.000 |
| ENSMUSG00000033420 | *Antxr1* | -2.663 | 0.000 | 0.000 |
| ENSMUSG00000050612 | *Txndc2* | 4.428 | 0.000 | 0.000 |
| ENSMUSG00000094799 | *AC125149.4* | -6.164 | 0.000 | 0.000 |
| ENSMUSG00000023484 | *Prph* | 4.618 | 0.000 | 0.000 |
| ENSMUSG00000059852 | *Kcng2* | 6.170 | 0.000 | 0.000 |
| ENSMUSG00000027249 | *F2* | 4.375 | 0.000 | 0.000 |
| ENSMUSG00000051228 | *Nyx* | 3.284 | 0.000 | 0.000 |
| ENSMUSG00000050014 | *Apol10b* | -5.930 | 0.000 | 0.000 |
| ENSMUSG00000025754 | *Agbl1* | -6.047 | 0.000 | 0.000 |
| ENSMUSG00000055567 | *Unc80* | -3.027 | 0.000 | 0.000 |
| ENSMUSG00000074004 | *B3gnt6* | 2.659 | 0.000 | 0.000 |
| ENSMUSG00000078922 | *Tgtp1* | -3.661 | 0.000 | 0.000 |
| ENSMUSG00000030701 | *Plekhb1* | 3.329 | 0.000 | 0.000 |
| ENSMUSG00000107928 | *Gm45140* | 3.329 | 0.000 | 0.000 |
| ENSMUSG00000095498 | *Ifna1* | -5.866 | 0.000 | 0.000 |
| ENSMUSG00000090551 | *A730015C16Rik* | 3.014 | 0.000 | 0.000 |
| ENSMUSG00000015484 | *Fam163a* | 5.868 | 0.000 | 0.000 |
| ENSMUSG00000039193 | *Nlrc4* | -2.291 | 0.000 | 0.000 |
| ENSMUSG00000074647 | *Fam83c* | 2.097 | 0.000 | 0.000 |
| ENSMUSG00000030895 | *Hpx* | 3.125 | 0.000 | 0.000 |
| ENSMUSG00000087444 | *Gm5475* | 3.162 | 0.000 | 0.000 |
| ENSMUSG00000027254 | *Map1a* | -3.282 | 0.000 | 0.000 |
| ENSMUSG00000026009 | *Icos* | -3.397 | 0.000 | 0.000 |
| ENSMUSG00000034037 | *Fgd5* | 3.285 | 0.000 | 0.000 |
| ENSMUSG00000041548 | *Hspb8* | 3.592 | 0.000 | 0.000 |
| ENSMUSG00000051136 | *Ghsr* | 4.322 | 0.000 | 0.000 |
| ENSMUSG00000020679 | *Hnf1b* | 3.693 | 0.000 | 0.000 |
| ENSMUSG00000033520 | *Idi2* | 4.209 | 0.000 | 0.000 |
| ENSMUSG00000114299 | *Gm47791* | 4.479 | 0.000 | 0.000 |
| ENSMUSG00000092518 | *Fam71e2* | 6.056 | 0.000 | 0.000 |
| ENSMUSG00000017897 | *Eya2* | 5.999 | 0.000 | 0.000 |
| ENSMUSG00000022753 | *Tmem30c* | 2.657 | 0.000 | 0.000 |
| ENSMUSG00000005089 | *Slc1a2* | 3.238 | 0.000 | 0.000 |
| ENSMUSG00000019935 | *Slc17a8* | 2.068 | 0.000 | 0.000 |
| ENSMUSG00000047117 | *Ankdd1b* | 3.330 | 0.000 | 0.000 |
| ENSMUSG00000029859 | *Epha1* | 4.265 | 0.000 | 0.000 |
| ENSMUSG00000039714 | *Cplx3* | 3.877 | 0.000 | 0.000 |
| ENSMUSG00000051648 | *Kctd19* | 3.189 | 0.000 | 0.000 |
| ENSMUSG00000103800 | *Pcdha8* | -2.691 | 0.000 | 0.000 |
| ENSMUSG00000028259 | *Fhl5* | 5.935 | 0.000 | 0.000 |
| ENSMUSG00000071226 | *Cecr2* | 3.284 | 0.000 | 0.000 |
| ENSMUSG00000032514 | *Ttc21a* | 3.416 | 0.000 | 0.000 |
| ENSMUSG00000016529 | *Il10* | 3.695 | 0.000 | 0.000 |
| ENSMUSG00000036995 | *Asap3* | -2.280 | 0.000 | 0.000 |
| ENSMUSG00000021640 | *Naip1* | -2.421 | 0.000 | 0.000 |
| ENSMUSG00000030124 | *Lag3* | 2.867 | 0.000 | 0.000 |
| ENSMUSG00000027070 | *Lrp2* | 3.538 | 0.000 | 0.000 |
| ENSMUSG00000050439 | *Enthd1* | -3.463 | 0.000 | 0.000 |
| ENSMUSG00000003354 | *Ccdc65* | 2.703 | 0.000 | 0.000 |
| ENSMUSG00000031303 | *Map3k15* | 2.620 | 0.000 | 0.000 |
| ENSMUSG00000015222 | *Map2* | -3.249 | 0.000 | 0.000 |
| ENSMUSG00000028836 | *Slc30a2* | 4.427 | 0.000 | 0.000 |
| ENSMUSG00000022218 | *Tgm1* | 2.374 | 0.000 | 0.000 |
| ENSMUSG00000045381 | *Olfr433* | -2.556 | 0.000 | 0.000 |
| ENSMUSG00000049741 | *Rxfp4* | 2.953 | 0.000 | 0.000 |
| ENSMUSG00000112129 | *Pbld1* | 5.933 | 0.000 | 0.000 |
| ENSMUSG00000060303 | *Olfr1447* | 5.727 | 0.000 | 0.000 |
| ENSMUSG00000044957 | *Pp2d1* | 5.727 | 0.000 | 0.000 |
| ENSMUSG00000001444 | *Tbx21* | 2.909 | 0.000 | 0.000 |
| ENSMUSG00000049649 | *Gpr3* | 2.442 | 0.000 | 0.000 |
| ENSMUSG00000030214 | *Plbd1* | 3.786 | 0.000 | 0.000 |
| ENSMUSG00000114865 | *Gm29776* | 2.160 | 0.000 | 0.000 |
| ENSMUSG00000029419 | *Ajm1* | 3.138 | 0.000 | 0.000 |
| ENSMUSG00000041460 | *Cacna2d4* | -2.800 | 0.000 | 0.000 |
| ENSMUSG00000040592 | *Cd79b* | 4.147 | 0.000 | 0.000 |
| ENSMUSG00000078234 | *Klhdc7a* | -2.385 | 0.000 | 0.000 |
| ENSMUSG00000031382 | *Asb11* | 3.083 | 0.000 | 0.000 |
| ENSMUSG00000052572 | *Dlg2* | 2.659 | 0.000 | 0.000 |
| ENSMUSG00000092470 | *Gm20518* | 2.066 | 0.000 | 0.000 |
| ENSMUSG00000042532 | *Golga7b* | 4.209 | 0.000 | 0.000 |
| ENSMUSG00000020081 | *Tacr2* | -3.566 | 0.000 | 0.000 |
| ENSMUSG00000028601 | *Echdc2* | -2.301 | 0.000 | 0.000 |
| ENSMUSG00000071398 | *2410004P03Rik* | 3.082 | 0.000 | 0.000 |
| ENSMUSG00000051788 | *4930564D02Rik* | 2.506 | 0.000 | 0.000 |
| ENSMUSG00000036912 | *Piwil4* | -5.660 | 0.000 | 0.000 |
| ENSMUSG00000013367 | *Iglon5* | 3.539 | 0.000 | 0.000 |
| ENSMUSG00000068113 | *Gm4907* | -2.609 | 0.000 | 0.000 |
| ENSMUSG00000026700 | *Tnfsf4* | 2.197 | 0.000 | 0.000 |
| ENSMUSG00000092120 | *Vmn2r90* | -5.581 | 0.000 | 0.000 |
| ENSMUSG00000034730 | *Adgrb1* | -2.774 | 0.000 | 0.000 |
| ENSMUSG00000027452 | *Acss1* | -2.589 | 0.000 | 0.000 |
| ENSMUSG00000014529 | *Tmbim7* | 3.089 | 0.000 | 0.000 |
| ENSMUSG00000051716 | *Apon* | -4.199 | 0.000 | 0.000 |
| ENSMUSG00000079323 | *Gm20661* | -3.651 | 0.000 | 0.000 |
| ENSMUSG00000041566 | *Tssk1* | 2.000 | 0.000 | 0.000 |
| ENSMUSG00000047511 | *Olfr1396* | -5.792 | 0.000 | 0.000 |
| ENSMUSG00000005237 | *Dnah2* | 2.659 | 0.000 | 0.000 |
| ENSMUSG00000042155 | *Klhl23* | -2.776 | 0.000 | 0.000 |
| ENSMUSG00000032105 | *Pdzd3* | 2.064 | 0.000 | 0.000 |
| ENSMUSG00000096528 | *G430049J08Rik* | -2.200 | 0.000 | 0.000 |
| ENSMUSG00000082101 | *Slfn14* | 2.705 | 0.000 | 0.000 |
| ENSMUSG00000019944 | *Rhobtb1* | -2.120 | 0.000 | 0.000 |
| ENSMUSG00000014813 | *Stc1* | -3.570 | 0.000 | 0.000 |
| ENSMUSG00000043939 | *A530064D06Rik* | -5.500 | 0.000 | 0.000 |
| ENSMUSG00000028860 | *Sytl1* | 2.499 | 0.000 | 0.000 |
| ENSMUSG00000073608 | *Gal3st2c* | 4.020 | 0.000 | 0.000 |
| ENSMUSG00000021303 | *Gng4* | 2.814 | 0.000 | 0.000 |
| ENSMUSG00000027886 | *1700013F07Rik* | 4.085 | 0.000 | 0.000 |
| ENSMUSG00000002289 | *Angptl4* | -2.203 | 0.000 | 0.000 |
| ENSMUSG00000030981 | *Mmp21* | 3.034 | 0.000 | 0.000 |
| ENSMUSG00000042359 | *Osbpl6* | 2.001 | 0.000 | 0.000 |
| ENSMUSG00000031022 | *BC051019* | 2.501 | 0.000 | 0.000 |
| ENSMUSG00000030606 | *Hapln3* | 3.283 | 0.000 | 0.000 |
| ENSMUSG00000062204 | *Olfr1373* | 5.569 | 0.000 | 0.000 |
| ENSMUSG00000044405 | *Adig* | 2.458 | 0.000 | 0.000 |
| ENSMUSG00000039543 | *Cfap70* | 2.370 | 0.000 | 0.000 |
| ENSMUSG00000028865 | *Cd164l2* | 4.151 | 0.000 | 0.000 |
| ENSMUSG00000025221 | *Kcnip2* | 4.150 | 0.000 | 0.000 |
| ENSMUSG00000063564 | *Col23a1* | 2.030 | 0.000 | 0.000 |
| ENSMUSG00000038201 | *Kcna7* | 2.175 | 0.000 | 0.000 |
| ENSMUSG00000100079 | *Ifnab* | 5.483 | 0.000 | 0.000 |
| ENSMUSG00000027209 | *Fam227b* | 5.484 | 0.000 | 0.000 |
| ENSMUSG00000049551 | *Fzd9* | -2.954 | 0.000 | 0.000 |
| ENSMUSG00000037161 | *Mgarp* | 2.617 | 0.000 | 0.000 |
| ENSMUSG00000062028 | *Irgc1* | 3.951 | 0.000 | 0.000 |
| ENSMUSG00000041062 | *Mslnl* | 3.537 | 0.000 | 0.000 |
| ENSMUSG00000074812 | *Spdye4c* | 5.569 | 0.000 | 0.000 |
| ENSMUSG00000036185 | *Sapcd1* | -2.110 | 0.000 | 0.000 |
| ENSMUSG00000036858 | *Ptcra* | 5.483 | 0.000 | 0.000 |
| ENSMUSG00000110195 | *Pde2a* | 2.187 | 0.000 | 0.000 |
| ENSMUSG00000091971 | *Hspa1a* | 5.484 | 0.000 | 0.000 |
| ENSMUSG00000023914 | *Mep1a* | 5.484 | 0.000 | 0.000 |
| ENSMUSG00000020122 | *Egfr* | 2.977 | 0.000 | 0.000 |
| ENSMUSG00000047181 | *Samd14* | 2.501 | 0.000 | 0.000 |
| ENSMUSG00000026818 | *Cel* | 2.149 | 0.000 | 0.000 |
| ENSMUSG00000026497 | *Mixl1* | 3.877 | 0.000 | 0.000 |
| ENSMUSG00000020176 | *Grb10* | 2.978 | 0.000 | 0.000 |
| ENSMUSG00000023236 | *Scg5* | 2.659 | 0.000 | 0.000 |
| ENSMUSG00000041889 | *Shisa4* | 2.956 | 0.000 | 0.000 |
| ENSMUSG00000018927 | *Ccl6* | -2.728 | 0.000 | 0.000 |
| ENSMUSG00000062077 | *Trim54* | 4.019 | 0.000 | 0.000 |
| ENSMUSG00000033177 | *Tmprss7* | 2.498 | 0.000 | 0.000 |
| ENSMUSG00000018916 | *Csf2* | -3.237 | 0.000 | 0.000 |
| ENSMUSG00000022683 | *Pla2g10* | 2.815 | 0.000 | 0.000 |
| ENSMUSG00000039838 | *Slc45a1* | 5.570 | 0.000 | 0.000 |
| ENSMUSG00000037129 | *Tmprss13* | -5.505 | 0.000 | 0.000 |
| ENSMUSG00000074028 | *Slc22a13* | 5.569 | 0.000 | 0.000 |
| ENSMUSG00000003665 | *Has1* | 3.300 | 0.000 | 0.000 |
| ENSMUSG00000020902 | *Ntn1* | 2.830 | 0.000 | 0.000 |
| ENSMUSG00000029530 | *Ccr9* | 2.000 | 0.000 | 0.000 |
| ENSMUSG00000035032 | *Nek11* | 3.426 | 0.000 | 0.000 |
| ENSMUSG00000051246 | *Msantd1* | 2.032 | 0.000 | 0.000 |
| ENSMUSG00000057530 | *Ece1* | -2.578 | 0.000 | 0.000 |
| ENSMUSG00000091017 | *Fam71a* | 2.095 | 0.000 | 0.000 |
| ENSMUSG00000078240 | *Gm3550* | -3.099 | 0.000 | 0.000 |
| ENSMUSG00000026976 | *Pax8* | 3.949 | 0.000 | 0.000 |
| ENSMUSG00000047343 | *Mettl21c* | 2.918 | 0.000 | 0.000 |
| ENSMUSG00000022144 | *Gdnf* | 4.018 | 0.000 | 0.000 |
| ENSMUSG00000095180 | *Rhox5* | -3.352 | 0.000 | 0.000 |
| ENSMUSG00000021221 | *Dpf3* | -2.278 | 0.000 | 0.000 |
| ENSMUSG00000057596 | *Trim30d* | -3.905 | 0.000 | 0.000 |
| ENSMUSG00000007480 | *Mc5r* | 3.801 | 0.000 | 0.001 |
| ENSMUSG00000026815 | *Gfi1b* | -5.417 | 0.000 | 0.001 |
| ENSMUSG00000040434 | *Large2* | 5.485 | 0.000 | 0.001 |
| ENSMUSG00000029359 | *Tesc* | 2.553 | 0.000 | 0.001 |
| ENSMUSG00000035448 | *Ccr3* | 2.976 | 0.000 | 0.001 |
| ENSMUSG00000040680 | *Kremen2* | -2.904 | 0.000 | 0.001 |
| ENSMUSG00000025069 | *Gsto2* | 2.369 | 0.000 | 0.001 |
| ENSMUSG00000048329 | *Mfsd6l* | -2.127 | 0.000 | 0.001 |
| ENSMUSG00000070509 | *Rgma* | 5.392 | 0.000 | 0.001 |
| ENSMUSG00000018581 | *Dnah11* | -2.087 | 0.000 | 0.001 |
| ENSMUSG00000032023 | *Jhy* | 5.393 | 0.000 | 0.001 |
| ENSMUSG00000022358 | *Fbxo32* | 2.860 | 0.000 | 0.001 |
| ENSMUSG00000068323 | *Slc4a5* | 2.142 | 0.000 | 0.001 |
| ENSMUSG00000038300 | *Pth2* | 5.296 | 0.000 | 0.001 |
| ENSMUSG00000028040 | *Efna4* | 2.701 | 0.000 | 0.001 |
| ENSMUSG00000037224 | *Zfyve28* | -2.524 | 0.000 | 0.001 |
| ENSMUSG00000045004 | *Spata21* | 2.366 | 0.000 | 0.001 |
| ENSMUSG00000030402 | *Ppm1n* | 3.032 | 0.000 | 0.001 |
| ENSMUSG00000021768 | *Dusp13* | 2.290 | 0.000 | 0.001 |
| ENSMUSG00000091735 | *Gpr62* | 2.189 | 0.000 | 0.001 |
| ENSMUSG00000005232 | *G6pc2* | 5.391 | 0.000 | 0.001 |
| ENSMUSG00000050108 | *Bpifc* | 5.393 | 0.000 | 0.001 |
| ENSMUSG00000095672 | *AC133103.5* | -2.411 | 0.000 | 0.001 |
| ENSMUSG00000033227 | *Wnt6* | -2.784 | 0.000 | 0.001 |
| ENSMUSG00000027669 | *Gnb4* | 2.007 | 0.000 | 0.001 |
| ENSMUSG00000063903 | *Klk1* | 5.296 | 0.000 | 0.001 |
| ENSMUSG00000035033 | *Tbr1* | 2.248 | 0.000 | 0.001 |
| ENSMUSG00000028874 | *Fgr* | -2.734 | 0.000 | 0.001 |
| ENSMUSG00000081512 | *Gm15821* | -2.847 | 0.000 | 0.001 |
| ENSMUSG00000016995 | *Matn4* | 3.299 | 0.000 | 0.001 |
| ENSMUSG00000020583 | *Matn3* | -2.663 | 0.000 | 0.001 |
| ENSMUSG00000035191 | *Rfpl4* | 3.719 | 0.000 | 0.001 |
| ENSMUSG00000075289 | *Carns1* | 2.272 | 0.000 | 0.001 |
| ENSMUSG00000026407 | *Cacna1s* | 3.720 | 0.000 | 0.001 |
| ENSMUSG00000020086 | *H2afy2* | 2.203 | 0.000 | 0.001 |
| ENSMUSG00000035951 | *Ascl3* | 3.236 | 0.000 | 0.001 |
| ENSMUSG00000029248 | *Thegl* | 2.609 | 0.000 | 0.001 |
| ENSMUSG00000035429 | *Ptprh* | 2.603 | 0.000 | 0.001 |
| ENSMUSG00000026874 | *Hc* | 5.191 | 0.000 | 0.001 |
| ENSMUSG00000086598 | *Btbd18* | 2.307 | 0.000 | 0.001 |
| ENSMUSG00000032726 | *Bmp8a* | 2.416 | 0.000 | 0.001 |
| ENSMUSG00000052316 | *Lrrc15* | 5.295 | 0.000 | 0.001 |
| ENSMUSG00000094786 | *Gm14403* | -3.215 | 0.001 | 0.001 |
| ENSMUSG00000031844 | *Hsd17b2* | 5.297 | 0.001 | 0.001 |
| ENSMUSG00000072680 | *Tmem254c* | -5.229 | 0.001 | 0.001 |
| ENSMUSG00000022485 | *Hoxc5* | -5.230 | 0.001 | 0.001 |
| ENSMUSG00000037347 | *Chst7* | -2.197 | 0.001 | 0.001 |
| ENSMUSG00000062826 | *Ces2f* | 2.797 | 0.001 | 0.001 |
| ENSMUSG00000015962 | *1700016C15Rik* | 2.859 | 0.001 | 0.001 |
| ENSMUSG00000050440 | *Hamp* | 3.801 | 0.001 | 0.001 |
| ENSMUSG00000020374 | *Rasgef1c* | 3.798 | 0.001 | 0.001 |
| ENSMUSG00000029517 | *Ankrd7* | 2.170 | 0.001 | 0.002 |
| ENSMUSG00000052301 | *Doc2a* | 2.978 | 0.001 | 0.002 |
| ENSMUSG00000019823 | *Mical1* | 2.062 | 0.001 | 0.002 |
| ENSMUSG00000040329 | *Il7* | -2.597 | 0.001 | 0.002 |
| ENSMUSG00000090659 | *Zfp493* | -2.350 | 0.001 | 0.002 |
| ENSMUSG00000090523 | *Gypc* | -5.121 | 0.001 | 0.002 |
| ENSMUSG00000114073 | *Gm30302* | 3.636 | 0.001 | 0.002 |
| ENSMUSG00000029769 | *Ccdc136* | 2.018 | 0.001 | 0.002 |
| ENSMUSG00000071856 | *Mcc* | -2.136 | 0.001 | 0.002 |
| ENSMUSG00000039391 | *Ccdc81* | 3.236 | 0.001 | 0.002 |
| ENSMUSG00000022286 | *Grhl2* | 3.230 | 0.001 | 0.002 |
| ENSMUSG00000030600 | *Lrfn1* | -2.362 | 0.001 | 0.002 |
| ENSMUSG00000022245 | *Skor1* | 5.079 | 0.001 | 0.002 |
| ENSMUSG00000053603 | *4930442H23Rik* | 5.079 | 0.001 | 0.002 |
| ENSMUSG00000050534 | *Htr5b* | 5.190 | 0.001 | 0.002 |
| ENSMUSG00000039742 | *Fam71f1* | 5.191 | 0.001 | 0.002 |
| ENSMUSG00000031448 | *Adprhl1* | 5.191 | 0.001 | 0.002 |
| ENSMUSG00000038173 | *Enpp6* | -5.119 | 0.001 | 0.002 |
| ENSMUSG00000020940 | *1700023F06Rik* | -5.115 | 0.001 | 0.002 |
| ENSMUSG00000025013 | *Tll2* | 5.193 | 0.001 | 0.002 |
| ENSMUSG00000051000 | *Fam160a1* | 2.658 | 0.001 | 0.002 |
| ENSMUSG00000028763 | *Hspg2* | 2.661 | 0.001 | 0.002 |
| ENSMUSG00000037139 | *Myom3* | 3.721 | 0.001 | 0.002 |
| ENSMUSG00000054934 | *Kcnmb4* | 3.720 | 0.001 | 0.002 |
| ENSMUSG00000021913 | *Ogdhl* | 3.720 | 0.001 | 0.002 |
| ENSMUSG00000049892 | *Rasd1* | 2.511 | 0.001 | 0.002 |
| ENSMUSG00000035785 | *Cmtm2b* | 3.634 | 0.001 | 0.002 |
| ENSMUSG00000027386 | *Fbln7* | 5.079 | 0.001 | 0.002 |
| ENSMUSG00000051590 | *Map3k19* | 2.661 | 0.001 | 0.003 |
| ENSMUSG00000027520 | *Zdbf2* | 3.163 | 0.001 | 0.003 |
| ENSMUSG00000051582 | *Otud6a* | 3.164 | 0.001 | 0.003 |
| ENSMUSG00000003070 | *Efna2* | 2.365 | 0.001 | 0.003 |
| ENSMUSG00000028451 | *1700022I11Rik* | 2.249 | 0.001 | 0.003 |
| ENSMUSG00000069792 | *Wfdc17* | -2.286 | 0.001 | 0.003 |
| ENSMUSG00000028539 | *Artn* | 2.472 | 0.001 | 0.003 |
| ENSMUSG00000039542 | *Ncam1* | 2.061 | 0.001 | 0.003 |
| ENSMUSG00000076435 | *Acsf2* | -3.511 | 0.001 | 0.003 |
| ENSMUSG00000096330 | *Gm21976* | 3.634 | 0.001 | 0.003 |
| ENSMUSG00000025196 | *Cpn1* | 3.636 | 0.001 | 0.003 |
| ENSMUSG00000033737 | *Fndc3c1* | -5.006 | 0.001 | 0.004 |
| ENSMUSG00000034690 | *Nlrp4c* | 5.079 | 0.001 | 0.004 |
| ENSMUSG00000012705 | *Retn* | 5.079 | 0.001 | 0.004 |
| ENSMUSG00000071540 | *3425401B19Rik* | -5.011 | 0.001 | 0.004 |
| ENSMUSG00000095266 | *Gm5225* | 5.078 | 0.001 | 0.004 |
| ENSMUSG00000092077 | *Olfr101* | 5.080 | 0.001 | 0.004 |
| ENSMUSG00000108929 | *Cc2d2b* | -5.008 | 0.001 | 0.004 |
| ENSMUSG00000031273 | *Col4a6* | -2.192 | 0.001 | 0.004 |
| ENSMUSG00000026117 | *Zap70* | 2.727 | 0.002 | 0.004 |
| ENSMUSG00000043301 | *Kcnj6* | 2.422 | 0.002 | 0.004 |
| ENSMUSG00000031379 | *Pir* | 2.119 | 0.002 | 0.004 |
| ENSMUSG00000009246 | *Trpm5* | 2.012 | 0.002 | 0.004 |
| ENSMUSG00000047878 | *A4galt* | 3.095 | 0.002 | 0.004 |
| ENSMUSG00000030134 | *Rasgef1a* | 2.249 | 0.002 | 0.004 |
| ENSMUSG00000043385 | *Olfr267* | 2.597 | 0.002 | 0.004 |
| ENSMUSG00000078656 | *Vps25* | -2.669 | 0.002 | 0.004 |
| ENSMUSG00000034706 | *Dnaic2* | 2.587 | 0.002 | 0.004 |
| ENSMUSG00000047696 | *Ccdc144b* | 3.446 | 0.002 | 0.004 |
| ENSMUSG00000040963 | *Asgr2* | 3.446 | 0.002 | 0.004 |
| ENSMUSG00000051998 | *Lax1* | 2.483 | 0.002 | 0.004 |
| ENSMUSG00000069873 | *4930438A08Rik* | 2.422 | 0.002 | 0.005 |
| ENSMUSG00000030098 | *Grip2* | 2.936 | 0.002 | 0.005 |
| ENSMUSG00000049692 | *Tmem239* | 3.015 | 0.002 | 0.005 |
| ENSMUSG00000018845 | *Unc45b* | -2.221 | 0.002 | 0.005 |
| ENSMUSG00000074483 | *Bglap* | 2.221 | 0.002 | 0.005 |
| ENSMUSG00000050395 | *Tnfsf15* | -4.887 | 0.002 | 0.005 |
| ENSMUSG00000079699 | *Gm6592* | -3.415 | 0.002 | 0.005 |
| ENSMUSG00000031837 | *Necab2* | 3.545 | 0.002 | 0.005 |
| ENSMUSG00000073402 | *Gm8909* | -3.415 | 0.002 | 0.005 |
| ENSMUSG00000029409 | *U90926* | 2.362 | 0.002 | 0.005 |
| ENSMUSG00000015854 | *Cd5l* | -2.886 | 0.002 | 0.006 |
| ENSMUSG00000028661 | *Epha8* | 3.018 | 0.002 | 0.006 |
| ENSMUSG00000073407 | *Gm6034* | -2.886 | 0.002 | 0.006 |
| ENSMUSG00000060176 | *Kif27* | 2.189 | 0.002 | 0.006 |
| ENSMUSG00000073530 | *Pappa2* | 4.956 | 0.002 | 0.006 |
| ENSMUSG00000055003 | *Lrtm2* | -4.889 | 0.002 | 0.006 |
| ENSMUSG00000091260 | *Vmn2r19* | 4.959 | 0.002 | 0.006 |
| ENSMUSG00000038143 | *Stox2* | -4.894 | 0.002 | 0.006 |
| ENSMUSG00000033350 | *Chst2* | 4.958 | 0.002 | 0.006 |
| ENSMUSG00000004842 | *Pou1f1* | 4.957 | 0.002 | 0.006 |
| ENSMUSG00000116930 | *AC144408.3* | 4.959 | 0.002 | 0.006 |
| ENSMUSG00000024421 | *Lama3* | -4.889 | 0.002 | 0.006 |
| ENSMUSG00000104713 | *Gbp6* | -3.328 | 0.003 | 0.006 |
| ENSMUSG00000028441 | *1110017D15Rik* | 2.591 | 0.003 | 0.006 |
| ENSMUSG00000029019 | *Nppb* | 2.511 | 0.003 | 0.007 |
| ENSMUSG00000048003 | *Catsper4* | 2.309 | 0.003 | 0.007 |
| ENSMUSG00000048191 | *Muc6* | 2.287 | 0.003 | 0.007 |
| ENSMUSG00000009115 | *Spatc1l* | 2.486 | 0.003 | 0.008 |
| ENSMUSG00000026882 | *4930568D16Rik* | -4.756 | 0.003 | 0.008 |
| ENSMUSG00000029370 | *Rassf6* | 3.446 | 0.003 | 0.008 |
| ENSMUSG00000029379 | *Cxcl3* | 3.447 | 0.003 | 0.008 |
| ENSMUSG00000029437 | *Il31* | 3.447 | 0.003 | 0.008 |
| ENSMUSG00000030244 | *Gys2* | 3.445 | 0.003 | 0.008 |
| ENSMUSG00000035165 | *Kcne3* | -3.328 | 0.003 | 0.008 |
| ENSMUSG00000021758 | *Ddx4* | 3.448 | 0.003 | 0.008 |
| ENSMUSG00000093865 | *Lrit3* | 3.342 | 0.004 | 0.008 |
| ENSMUSG00000025197 | *Cyp2c23* | 3.343 | 0.004 | 0.008 |
| ENSMUSG00000027460 | *Angpt4* | -2.806 | 0.004 | 0.008 |
| ENSMUSG00000046470 | *Sox18* | 2.936 | 0.004 | 0.008 |
| ENSMUSG00000056025 | *Clca3a1* | 2.934 | 0.004 | 0.008 |
| ENSMUSG00000028602 | *Tnfrsf8* | 2.936 | 0.004 | 0.008 |
| ENSMUSG00000071335 | *Mfsd4b3* | 2.937 | 0.004 | 0.008 |
| ENSMUSG00000009075 | *Cabp7* | 2.934 | 0.004 | 0.008 |
| ENSMUSG00000047108 | *Dnajb7* | -2.800 | 0.004 | 0.008 |
| ENSMUSG00000026573 | *Xcl1* | 2.588 | 0.004 | 0.009 |
| ENSMUSG00000026070 | *Il18r1* | -2.041 | 0.004 | 0.009 |
| ENSMUSG00000078197 | *Gm17374* | 3.343 | 0.004 | 0.009 |
| ENSMUSG00000024063 | *Lbh* | 2.587 | 0.004 | 0.010 |
| ENSMUSG00000043468 | *Adam30* | 2.759 | 0.004 | 0.010 |
| ENSMUSG00000101750 | *Olfr1392* | 2.760 | 0.004 | 0.010 |
| ENSMUSG00000113973 | *A030014E15Rik* | 4.826 | 0.004 | 0.010 |
| ENSMUSG00000026866 | *Kynu* | -4.760 | 0.004 | 0.010 |
| ENSMUSG00000057378 | *Ryr3* | 4.825 | 0.004 | 0.010 |
| ENSMUSG00000027297 | *Ltk* | 4.825 | 0.004 | 0.010 |
| ENSMUSG00000027978 | *Prss12* | 4.826 | 0.004 | 0.010 |
| ENSMUSG00000027967 | *Neurog2* | 4.823 | 0.004 | 0.010 |
| ENSMUSG00000040016 | *Ptger3* | 4.824 | 0.004 | 0.010 |
| ENSMUSG00000063415 | *Cyp26b1* | 4.825 | 0.004 | 0.010 |
| ENSMUSG00000030162 | *Olr1* | 4.825 | 0.004 | 0.010 |
| ENSMUSG00000031512 | *Tex29* | 4.824 | 0.004 | 0.010 |
| ENSMUSG00000032313 | *Tmem266* | 4.826 | 0.004 | 0.010 |
| ENSMUSG00000039238 | *Zfp750* | -4.760 | 0.004 | 0.010 |
| ENSMUSG00000041347 | *Bdkrb1* | 4.824 | 0.004 | 0.010 |
| ENSMUSG00000060284 | *Sp7* | -4.759 | 0.004 | 0.010 |
| ENSMUSG00000038146 | *Notch3* | 4.825 | 0.004 | 0.010 |
| ENSMUSG00000052942 | *Glis3* | 4.824 | 0.004 | 0.010 |
| ENSMUSG00000027481 | *Bpifb2* | 2.417 | 0.004 | 0.010 |
| ENSMUSG00000034362 | *Csta1* | 2.421 | 0.004 | 0.010 |
| ENSMUSG00000034584 | *Exph5* | -2.149 | 0.005 | 0.010 |
| ENSMUSG00000074232 | *Gm10647* | 2.004 | 0.005 | 0.011 |
| ENSMUSG00000034220 | *Gpc1* | -2.459 | 0.005 | 0.011 |
| ENSMUSG00000036687 | *Tmem184a* | 2.587 | 0.005 | 0.011 |
| ENSMUSG00000078796 | *Zfp541* | -2.464 | 0.005 | 0.011 |
| ENSMUSG00000075480 | *Gm10840* | 2.758 | 0.005 | 0.011 |
| ENSMUSG00000015355 | *Cd48* | -2.081 | 0.005 | 0.012 |
| ENSMUSG00000048617 | *Rtbdn* | 2.059 | 0.005 | 0.012 |
| ENSMUSG00000044787 | *Spata32* | -2.724 | 0.005 | 0.012 |
| ENSMUSG00000020871 | *Dlx4* | -2.632 | 0.005 | 0.012 |
| ENSMUSG00000026736 | *4930426L09Rik* | -2.726 | 0.005 | 0.012 |
| ENSMUSG00000034219 | *Septin14* | 2.852 | 0.005 | 0.012 |
| ENSMUSG00000095040 | *1700001J03Rik* | 2.850 | 0.005 | 0.012 |
| ENSMUSG00000059900 | *Tmem40* | 2.847 | 0.005 | 0.012 |
| ENSMUSG00000000263 | *Glra1* | 2.849 | 0.005 | 0.012 |
| ENSMUSG00000024738 | *Pga5* | 2.847 | 0.005 | 0.012 |
| ENSMUSG00000053297 | *AI854703* | 2.128 | 0.005 | 0.012 |
| ENSMUSG00000027077 | *Smtnl1* | -3.226 | 0.005 | 0.012 |
| ENSMUSG00000032548 | *Slco2a1* | -3.219 | 0.005 | 0.012 |
| ENSMUSG00000049926 | *Olfr921* | 2.430 | 0.005 | 0.012 |
| ENSMUSG00000033208 | *S100b* | 2.002 | 0.005 | 0.012 |
| ENSMUSG00000100512 | *Ovol3* | 3.230 | 0.005 | 0.012 |
| ENSMUSG00000050578 | *Mmp13* | 2.289 | 0.006 | 0.013 |
| ENSMUSG00000031101 | *Sash3* | -2.082 | 0.006 | 0.013 |
| ENSMUSG00000105078 | *Vamp9* | 4.678 | 0.006 | 0.014 |
| ENSMUSG00000058626 | *Capn11* | 3.229 | 0.006 | 0.014 |
| ENSMUSG00000006411 | *Nectin4* | -2.026 | 0.006 | 0.014 |
| ENSMUSG00000030781 | *Slc5a2* | 2.433 | 0.006 | 0.014 |
| ENSMUSG00000049699 | *Ucn2* | 2.511 | 0.007 | 0.016 |
| ENSMUSG00000037727 | *Avp* | 2.759 | 0.007 | 0.016 |
| ENSMUSG00000057072 | *Spata45* | 4.677 | 0.007 | 0.016 |
| ENSMUSG00000058740 | *Kcnt1* | 4.678 | 0.007 | 0.016 |
| ENSMUSG00000026980 | *Ly75* | 4.679 | 0.007 | 0.016 |
| ENSMUSG00000014361 | *Mertk* | 4.678 | 0.007 | 0.016 |
| ENSMUSG00000033634 | *Nat8f2* | 4.680 | 0.007 | 0.016 |
| ENSMUSG00000042873 | *Lhfpl4* | 4.680 | 0.007 | 0.016 |
| ENSMUSG00000093973 | *Mrgpra2a* | -4.611 | 0.007 | 0.016 |
| ENSMUSG00000047712 | *Ust* | -4.611 | 0.007 | 0.016 |
| ENSMUSG00000002341 | *Ncan* | 4.678 | 0.007 | 0.016 |
| ENSMUSG00000061356 | *Nuggc* | -4.615 | 0.007 | 0.016 |
| ENSMUSG00000047508 | *Mbd3l2* | 4.678 | 0.007 | 0.016 |
| ENSMUSG00000031994 | *Adamts8* | 4.679 | 0.007 | 0.016 |
| ENSMUSG00000033491 | *Prss35* | -4.607 | 0.007 | 0.016 |
| ENSMUSG00000078924 | *Gm12169* | 4.677 | 0.007 | 0.016 |
| ENSMUSG00000041165 | *Spem1* | 4.679 | 0.007 | 0.016 |
| ENSMUSG00000025165 | *Sectm1a* | -4.613 | 0.007 | 0.016 |
| ENSMUSG00000005124 | *Wisp1* | 4.678 | 0.007 | 0.016 |
| ENSMUSG00000022816 | *Fstl1* | 4.679 | 0.007 | 0.016 |
| ENSMUSG00000040899 | *Ccr6* | 4.677 | 0.007 | 0.016 |
| ENSMUSG00000039153 | *Runx2* | 4.677 | 0.007 | 0.016 |
| ENSMUSG00000103472 | *Pcdhga7* | -4.608 | 0.007 | 0.016 |
| ENSMUSG00000067872 | *Ccdc87* | 4.678 | 0.007 | 0.016 |
| ENSMUSG00000033256 | *Shf* | -2.050 | 0.007 | 0.017 |
| ENSMUSG00000033805 | *Ephx4* | 2.344 | 0.007 | 0.017 |
| ENSMUSG00000022603 | *Mroh4* | -2.213 | 0.008 | 0.017 |
| ENSMUSG00000069601 | *Ank3* | -2.298 | 0.008 | 0.017 |
| ENSMUSG00000030433 | *Sbk2* | 2.760 | 0.008 | 0.018 |
| ENSMUSG00000078127 | *Fam170b* | 2.758 | 0.008 | 0.018 |
| ENSMUSG00000070345 | *Hsf5* | -2.621 | 0.008 | 0.018 |
| ENSMUSG00000040505 | *Abcg5* | 2.758 | 0.008 | 0.018 |
| ENSMUSG00000056124 | *B4galt6* | -2.630 | 0.008 | 0.018 |
| ENSMUSG00000025977 | *Boll* | 2.346 | 0.008 | 0.018 |
| ENSMUSG00000036856 | *Wnt4* | 3.231 | 0.008 | 0.019 |
| ENSMUSG00000043085 | *Tmem82* | 3.232 | 0.008 | 0.019 |
| ENSMUSG00000032068 | *Plet1* | -3.115 | 0.008 | 0.019 |
| ENSMUSG00000040740 | *Slc25a34* | 2.136 | 0.010 | 0.021 |
| ENSMUSG00000042453 | *Reln* | 2.663 | 0.010 | 0.021 |
| ENSMUSG00000037346 | *Hrh4* | 3.108 | 0.010 | 0.022 |
| ENSMUSG00000078496 | *Zfp982* | -2.536 | 0.010 | 0.023 |
| ENSMUSG00000052105 | *Mtcl1* | -2.076 | 0.011 | 0.024 |
| ENSMUSG00000062296 | *Trank1* | 2.660 | 0.012 | 0.026 |
| ENSMUSG00000030278 | *Cidec* | 2.212 | 0.012 | 0.026 |
| ENSMUSG00000031503 | *Col4a2* | 2.346 | 0.012 | 0.027 |
| ENSMUSG00000089942 | *Pira2* | -2.430 | 0.013 | 0.027 |
| ENSMUSG00000027612 | *Mmp24* | 4.514 | 0.013 | 0.027 |
| ENSMUSG00000079450 | *Cldn34c1* | 4.516 | 0.013 | 0.027 |
| ENSMUSG00000054679 | *Srsf12* | 4.514 | 0.013 | 0.027 |
| ENSMUSG00000066178 | *6030445D17Rik* | -4.446 | 0.013 | 0.027 |
| ENSMUSG00000043410 | *Hfm1* | 4.515 | 0.013 | 0.027 |
| ENSMUSG00000021903 | *Galnt15* | 4.516 | 0.013 | 0.027 |
| ENSMUSG00000020686 | *Gas2l2* | 4.514 | 0.013 | 0.027 |
| ENSMUSG00000034177 | *Rnf43* | 4.514 | 0.013 | 0.027 |
| ENSMUSG00000075602 | *Ly6a* | -4.451 | 0.013 | 0.027 |
| ENSMUSG00000022483 | *Col2a1* | 4.515 | 0.013 | 0.027 |
| ENSMUSG00000023000 | *Dhh* | 4.516 | 0.013 | 0.027 |
| ENSMUSG00000009097 | *Tbx1* | -4.443 | 0.013 | 0.027 |
| ENSMUSG00000045761 | *Togaram2* | 4.516 | 0.013 | 0.027 |
| ENSMUSG00000024485 | *Slc4a9* | 4.516 | 0.013 | 0.027 |
| ENSMUSG00000002104 | *Rapsn* | 3.106 | 0.014 | 0.029 |
| ENSMUSG00000025488 | *Cox8b* | 3.110 | 0.014 | 0.029 |
| ENSMUSG00000048368 | *Omd* | 3.109 | 0.014 | 0.029 |
| ENSMUSG00000021565 | *Slc6a19* | 3.108 | 0.014 | 0.029 |
| ENSMUSG00000021078 | *Tomm20l* | 3.110 | 0.014 | 0.029 |
| ENSMUSG00000096458 | *Moap1* | 3.110 | 0.014 | 0.029 |
| ENSMUSG00000115388 | *Eppk1* | -2.995 | 0.014 | 0.029 |
| ENSMUSG00000095687 | *Rnaset2a* | 3.109 | 0.014 | 0.029 |
| ENSMUSG00000000183 | *Fgf6* | 2.343 | 0.015 | 0.031 |
| ENSMUSG00000020963 | *Tshr* | 2.344 | 0.015 | 0.031 |
| ENSMUSG00000090231 | *Cfb* | -2.209 | 0.015 | 0.031 |
| ENSMUSG00000056043 | *Rgs9bp* | -2.009 | 0.017 | 0.035 |
| ENSMUSG00000034683 | *Ppp1r1c* | 2.977 | 0.018 | 0.038 |
| ENSMUSG00000090777 | *Ccdc188* | 2.976 | 0.018 | 0.038 |
| ENSMUSG00000040896 | *Kcnd3* | 2.555 | 0.018 | 0.038 |
| ENSMUSG00000023964 | *Calcr* | 2.557 | 0.018 | 0.038 |
| ENSMUSG00000029685 | *Asb15* | 2.556 | 0.018 | 0.038 |
| ENSMUSG00000019990 | *Pde7b* | -2.432 | 0.018 | 0.038 |
| ENSMUSG00000069855 | *Slc47a2* | -2.434 | 0.018 | 0.038 |
| ENSMUSG00000022504 | *Ciita* | 2.561 | 0.018 | 0.038 |
| ENSMUSG00000064016 | *Gm648* | 2.446 | 0.020 | 0.041 |
| ENSMUSG00000040841 | *Six5* | -2.316 | 0.020 | 0.041 |
| ENSMUSG00000007888 | *Crlf1* | 2.157 | 0.020 | 0.042 |
| ENSMUSG00000036855 | *Gjd4* | 2.253 | 0.021 | 0.043 |
| ENSMUSG00000094995 | *Pate3* | 2.252 | 0.021 | 0.044 |
| ENSMUSG00000075405 | *9430097D07Rik* | 2.975 | 0.022 | 0.045 |
| ENSMUSG00000027221 | *Chst1* | -2.857 | 0.022 | 0.045 |
| ENSMUSG00000040229 | *Gpr34* | -2.861 | 0.022 | 0.045 |
| ENSMUSG00000051257 | *Trap1a* | -2.855 | 0.022 | 0.045 |
| ENSMUSG00000046213 | *Cym* | 2.979 | 0.022 | 0.045 |
| ENSMUSG00000067702 | *Tuba3a* | 2.979 | 0.022 | 0.045 |
| ENSMUSG00000092622 | *Khdc3* | 2.974 | 0.022 | 0.045 |
| ENSMUSG00000040950 | *Mgl2* | 2.974 | 0.022 | 0.045 |
| ENSMUSG00000017417 | *Plxdc1* | 2.977 | 0.022 | 0.045 |
| ENSMUSG00000069306 | *Hist1h4m* | 2.978 | 0.022 | 0.045 |
| ENSMUSG00000021388 | *Aspn* | 2.975 | 0.022 | 0.045 |
| ENSMUSG00000038725 | *Pkhd1l1* | 2.978 | 0.022 | 0.045 |
| ENSMUSG00000022382 | *Wnt7b* | -2.857 | 0.022 | 0.045 |
| ENSMUSG00000022763 | *Aifm3* | 2.975 | 0.022 | 0.045 |
| ENSMUSG00000042729 | *Wdr74* | 2.974 | 0.022 | 0.045 |
| ENSMUSG00000026592 | *Tex35* | 2.445 | 0.023 | 0.047 |
| ENSMUSG00000047182 | *Irs3* | 2.444 | 0.023 | 0.047 |
| ENSMUSG00000104158 | *Gm38100* | 4.332 | 0.023 | 0.047 |
| ENSMUSG00000027073 | *Prg2* | 4.330 | 0.023 | 0.047 |
| ENSMUSG00000074997 | *Pin1rt1* | 4.332 | 0.023 | 0.047 |
| ENSMUSG00000027457 | *Snph* | 4.332 | 0.023 | 0.047 |
| ENSMUSG00000027546 | *Atp9a* | 4.330 | 0.023 | 0.047 |
| ENSMUSG00000027530 | *Fabp12* | 4.331 | 0.023 | 0.047 |
| ENSMUSG00000023057 | *Fabp2* | 4.332 | 0.023 | 0.047 |
| ENSMUSG00000012042 | *4930579F01Rik* | 4.331 | 0.023 | 0.047 |
| ENSMUSG00000025328 | *Padi3* | -4.263 | 0.023 | 0.047 |
| ENSMUSG00000038552 | *Fndc4* | 4.331 | 0.023 | 0.047 |
| ENSMUSG00000029096 | *Htra3* | 4.331 | 0.023 | 0.047 |
| ENSMUSG00000029380 | *Cxcl1* | 4.332 | 0.023 | 0.047 |
| ENSMUSG00000091450 | *Vmn2r11* | -4.259 | 0.023 | 0.047 |
| ENSMUSG00000052861 | *Dnah6* | 4.332 | 0.023 | 0.047 |
| ENSMUSG00000030200 | *Bcl2l14* | -4.268 | 0.023 | 0.047 |
| ENSMUSG00000110040 | *Gm49369* | 4.330 | 0.023 | 0.047 |
| ENSMUSG00000001119 | *Col6a1* | -4.264 | 0.023 | 0.047 |
| ENSMUSG00000031563 | *Wwc2* | 4.332 | 0.023 | 0.047 |
| ENSMUSG00000063821 | *Dupd1* | 4.330 | 0.023 | 0.047 |
| ENSMUSG00000054360 | *Bsx* | 4.332 | 0.023 | 0.047 |
| ENSMUSG00000036814 | *Slc6a20a* | 4.332 | 0.023 | 0.047 |
| ENSMUSG00000078668 | *Gm11595* | 4.332 | 0.023 | 0.047 |
| ENSMUSG00000051043 | *Gprc5c* | -4.263 | 0.023 | 0.047 |
| ENSMUSG00000095217 | *Hist1h2bn* | 4.332 | 0.023 | 0.047 |
| ENSMUSG00000069308 | *Hist1h2bp* | -4.271 | 0.023 | 0.047 |
| ENSMUSG00000089678 | *Agxt2* | 4.331 | 0.023 | 0.047 |
| ENSMUSG00000116594 | *AC133488.1* | -4.266 | 0.023 | 0.047 |
| ENSMUSG00000091212 | *Krtap11-1* | -4.262 | 0.023 | 0.047 |
| ENSMUSG00000039672 | *Kcne2* | -4.260 | 0.023 | 0.047 |
| ENSMUSG00000045027 | *Prss22* | 4.331 | 0.023 | 0.047 |
| ENSMUSG00000103749 | *Pcdhgb5* | -4.267 | 0.023 | 0.047 |
| ENSMUSG00000024901 | *Peli3* | 4.332 | 0.023 | 0.047 |
| ENSMUSG00000025219 | *Fgf8* | 4.333 | 0.023 | 0.047 |
| ENSMUSG00000025946 | *Pth2r* | 2.059 | 0.023 | 0.048 |
| ENSMUSG00000026567 | *Adcy10* | 2.054 | 0.023 | 0.048 |
| ENSMUSG00000057914 | *Cacnb2* | 2.056 | 0.023 | 0.048 |
| ENSMUSG00000042761 | *Mrap2* | 2.051 | 0.023 | 0.048 |

# 0.000 means < 0.0001.
