# Supplementary material for: Synergistic inflammatory signaling by cGAS may be involved in the development of atherosclerosis
Source: Aging (Albany NY). 2021 Feb 11;13(4):5650–73. doi: 10.18632/aging.202491 (PMC7950297; doi:10.18632/aging.202491)
Supplement: Supplementary Table 3 [file aging-13-202491-s004.docx]

**Supplementary Table 3. Genes related to atherosclerosis.**

| **Gene_ID** | **Gene_name** | | **log2FC** | | ***P v*alue** | | ***P*adj** | **References** |
| --- | --- | --- | --- | --- | --- | --- | --- | --- |
| ENSMUSG00000015837 | | *Sqstm1* | | 2.671 | 0.000 | 0.000 | | [1-3] |
| ENSMUSG00000070327 | | *Rnf213* | | -3.729 | 0.000 | 0.000 | | [4] |
| ENSMUSG00000028691 | | *Prdx1* | | 2.246 | 0.000 | 0.000 | | [5] |
| ENSMUSG00000058427 | | *Cxcl2* | | 4.226 | 0.000 | 0.000 | | [6, 7] |
| ENSMUSG00000025203 | | *Scd2* | | -4.570 | 0.000 | 0.000 | | [8] |
| ENSMUSG00000005413 | | *Hmox1* | | 2.911 | 0.000 | 0.000 | | [9, 10] |
| ENSMUSG00000024621 | | *Csf1r* | | -2.026 | 0.000 | 0.000 | | [11] |
| ENSMUSG00000015568 | | *Lpl* | | -3.524 | 0.000 | 0.000 | | [12] |
| ENSMUSG00000032487 | | *Ptgs2* | | 2.184 | 0.000 | 0.000 | | [13] |
| ENSMUSG00000003541 | | *Ier3* | | 3.404 | 0.000 | 0.000 | | [3] |
| ENSMUSG00000027737 | | *Slc7a11* | | 2.693 | 0.000 | 0.000 | | [14] |
| ENSMUSG00000028124 | | *Gclm* | | 2.144 | 0.000 | 0.000 | | [15] |
| ENSMUSG00000027009 | | *Itga4* | | -2.867 | 0.000 | 0.000 | | [16] |
| ENSMUSG00000034422 | | *Parp14* | | -5.062 | 0.000 | 0.000 | | [17] |
| ENSMUSG00000032661 | | *Oas3* | | -5.934 | 0.000 | 0.000 | | [18] |
| ENSMUSG00000027763 | | *Mbnl1* | | -2.321 | 0.000 | 0.000 | | [19] |
| ENSMUSG00000004266 | | *Ptpn6* | | -2.376 | 0.000 | 0.000 | | [20, 21] |
| ENSMUSG00000027639 | | *Samhd1* | | -2.469 | 0.000 | 0.000 | | [22] |
| ENSMUSG00000021670 | | *Hmgcr* | | -2.641 | 0.000 | 0.000 | | [23] |
| ENSMUSG00000030142 | | *Clec4e* | | 2.664 | 0.000 | 0.000 | | [24] |
| ENSMUSG00000045932 | | *Ifit2* | | -7.743 | 0.000 | 0.000 | | [25] |
| ENSMUSG00000025498 | | *Irf7* | | -6.012 | 0.000 | 0.000 | | [26] |
| ENSMUSG00000010663 | | *Fads1* | | -2.104 | 0.000 | 0.000 | | [27] |
| ENSMUSG00000026981 | | *Il1rn* | | 2.817 | 0.000 | 0.000 | | [28] |
| ENSMUSG00000021306 | | *Gpr137b* | | 2.320 | 0.000 | 0.000 | | [29] |
| ENSMUSG00000070348 | | *Ccnd1* | | -3.485 | 0.000 | 0.000 | | [30] |
| ENSMUSG00000002307 | | *Daxx* | | -2.562C | 0.000 | 0.000 | | [31] |
| ENSMUSG00000032802 | | *Srxn1* | | 2.293 | 0.000 | 0.000 | | [32] |
| ENSMUSG00000052684 | | *Jun* | | 2.678 | 0.000 | 0.000 | | [33] |
| ENSMUSG00000024665 | | *Fads2* | | -3.542 | 0.000 | 0.000 | | [34] |
| ENSMUSG00000032193 | | *Ldlr* | | -5.955 | 0.000 | 0.000 | | [35] |
| ENSMUSG00000028037 | | *Ifi44* | | -6.939 | 0.000 | 0.000 | | [36] |
| ENSMUSG00000028494 | | *Plin2* | | 2.060 | 0.000 | 0.000 | | [37] |
| ENSMUSG00000015340 | | *Cybb* | | -2.465 | 0.000 | 0.000 | | [38] |
| ENSMUSG00000046879 | | *Irgm1* | | -4.777 | 0.000 | 0.000 | | [39] |
| ENSMUSG00000033105 | | *Lss* | | -2.437 | 0.000 | 0.000 | | [40] |
| ENSMUSG00000032690 | | *Oas2* | | -5.713 | 0.000 | 0.000 | | [18] |
| ENSMUSG00000001467 | | *Cyp51* | | -3.720 | 0.000 | 0.000 | | [41] |
| ENSMUSG00000022351 | | *Sqle* | | -2.406 | 0.000 | 0.000 | | [42] |
| ENSMUSG00000036103 | | *Colec12* | | 2.140 | 0.000 | 0.000 | | [43] |
| ENSMUSG00000040033 | | *Stat2* | | -3.686 | 0.000 | 0.000 | | [18] |
| ENSMUSG00000020641 | | *Rsad2* | | -5.071 | 0.000 | 0.000 | | [44] |
| ENSMUSG00000024190 | | *Dusp1* | | 4.728 | 0.000 | 0.000 | | [45] |
| ENSMUSG00000015947 | | *Fcgr1* | | -2.239 | 0.000 | 0.000 | | [46] |
| ENSMUSG00000035692 | | *Isg15* | | -3.323 | 0.000 | 0.000 | | [47, 48] |
| ENSMUSG00000063268 | | *Parp10* | | -4.420 | 0.000 | 0.000 | | [49, 50] |
| ENSMUSG00000033880 | | *Lgals3bp* | | -5.499 | 0.000 | 0.000 | | [51] |
| ENSMUSG00000069516 | | *Lyz2* | | -2.002 | 0.000 | 0.000 | | [52] |
| ENSMUSG00000022150 | | *Dab2* | | -2.266 | 0.000 | 0.000 | | [53] |
| ENSMUSG00000078566 | | *Bnip3* | | 3.574 | 0.000 | 0.000 | | [54] |
| ENSMUSG00000053470 | | *Kdm3a* | | 2.461 | 0.000 | 0.000 | | [55] |
| ENSMUSG00000029771 | | *Irf5* | | -2.048 | 0.000 | 0.000 | | [56] |
| ENSMUSG00000034926 | | *Dhcr24* | | -3.700 | 0.000 | 0.000 | | [57] |
| ENSMUSG00000002233 | | *Rhoc* | | 2.041 | 0.000 | 0.000 | | [58] |
| ENSMUSG00000034459 | | *Ifit1* | | -7.153 | 0.000 | 0.000 | | [25] |
| ENSMUSG00000022906 | | *Parp9* | | -4.015 | 0.000 | 0.000 | | [17] |
| ENSMUSG00000005583 | | *Mef2c* | | -3.227 | 0.000 | 0.000 | | [59] |
| ENSMUSG00000037936 | | *Scarb1* | | -3.033 | 0.000 | 0.000 | | [60] |
| ENSMUSG00000037071 | | *Scd1* | | -6.491 | 0.000 | 0.000 | | [61] |
| ENSMUSG00000017830 | | *Dhx58* | | -5.650 | 0.000 | 0.000 | | [62] |
| ENSMUSG00000018899 | | *Irf1* | | -3.848 | 0.000 | 0.000 | | [62, 63] |
| ENSMUSG00000058755 | | *Osm* | | 2.957 | 0.000 | 0.000 | | [64] |
| ENSMUSG00000093930 | | *Hmgcs1* | | -2.911 | 0.000 | 0.000 | | [41] |
| ENSMUSG00000034118 | | *Tpst1* | | -3.161 | 0.000 | 0.000 | | [65] |
| ENSMUSG00000022951 | | *Rcan1* | | 2.455 | 0.000 | 0.000 | | [66] |
| ENSMUSG00000018217 | | *Pmp22* | | 2.216 | 0.000 | 0.000 | | [67] |
| ENSMUSG00000005124 | | *Wisp1* | | 4.678 | 0.000 | 0.000 | | [68] |
| ENSMUSG00000024399 | | *Ltb* | | -3.237 | 0.000 | 0.000 | | [69] |
| ENSMUSG00000023832 | | *Acat2* | | -3.773 | 0.000 | 0.000 | | [70] |
| ENSMUSG00000026675 | | *Hsd17b7* | | -2.593 | 0.000 | 0.000 | | [41] |
| ENSMUSG00000058258 | | *Idi1* | | -3.965 | 0.000 | 0.000 | | [71] |
| ENSMUSG00000001123 | | *Lgals9* | | -5.905 | 0.000 | 0.000 | | [51] |
| ENSMUSG00000041220 | | *Elovl6* | | -3.663 | 0.000 | 0.000 | | [72] |
| ENSMUSG00000010358 | | *Ifi35* | | -3.776 | 0.000 | 0.000 | | [73] |
| ENSMUSG00000031349 | | *Nsdhl* | | -3.429 | 0.000 | 0.000 | | [74] |
| ENSMUSG00000039853 | | *Trim14* | | -3.127 | 0.000 | 0.000 | | [75] |
| ENSMUSG00000026104 | | *Stat1* | | -4.464 | 0.000 | 0.000 | | [76, 77] |
| ENSMUSG00000043421 | | *Hilpda* | | 3.491 | 0.000 | 0.000 | | [78] |
| ENSMUSG00000052336 | | *Cx3cr1* | | -4.280 | 0.000 | 0.000 | | [79] |
| ENSMUSG00000030966 | | *Trim21* | | -4.673 | 0.000 | 0.000 | | [80] |
| ENSMUSG00000003545 | | *Fosb* | | 4.454 | 0.000 | 0.000 | | [81] |
| ENSMUSG00000058454 | | *Dhcr7* | | -2.724 | 0.000 | 0.000 | | [82] |
| ENSMUSG00000044583 | | *Tlr7* | | -2.055 | 0.000 | 0.000 | | [83-85] |
| ENSMUSG00000026896 | | *Ifih1* | | -2.693 | 0.000 | 0.000 | | [86] |
| ENSMUSG00000002325 | | *Irf9* | | -2.327 | 0.000 | 0.000 | | [87] |
| ENSMUSG00000028599 | | *Tnfrsf1b* | | 2.026 | 0.000 | 0.000 | | [88] |
| ENSMUSG00000026773 | | *Pfkfb3* | | 2.062 | 0.000 | 0.000 | | [89] |
| ENSMUSG00000023905 | | *Tnfrsf12a* | | 2.789 | 0.000 | 0.000 | | [90] |
| ENSMUSG00000034765 | | *Dusp5* | | 2.200 | 0.000 | 0.000 | | [91] |
| ENSMUSG00000042082 | | *Arsb* | | -2.358 | 0.000 | 0.000 | | [92] |
| ENSMUSG00000079017 | | *Ifi27l2a* | | -5.677 | 0.000 | 0.000 | | [18] |
| ENSMUSG00000048779 | | *P2ry6* | | -5.681 | 0.000 | 0.000 | | [93] |
| ENSMUSG00000026826 | | *Nr4a2* | | 3.022 | 0.000 | 0.000 | | [94] |
| ENSMUSG00000030102 | | *Itpr1* | | -2.028 | 0.000 | 0.000 | | [95] |
| ENSMUSG00000027605 | | *Acss2* | | -3.218 | 0.000 | 0.000 | | [71] |
| ENSMUSG00000041939 | | *Mvk* | | -2.707 | 0.000 | 0.000 | | [96] |
| ENSMUSG00000025044 | | *Msr1* | | -2.812 | 0.000 | 0.000 | | [70, 97] |
| ENSMUSG00000027322 | | *Siglec1* | | -7.931 | 0.000 | 0.000 | | [98] |
| ENSMUSG00000028238 | | *Atp6v0d2* | | 2.957 | 0.000 | 0.000 | | [99] |
| ENSMUSG00000027951 | | *Adar* | | -2.102 | 0.000 | 0.000 | | [100] |
| ENSMUSG00000004791 | | *Pgf* | | 5.765 | 0.000 | 0.000 | | [101] |
| ENSMUSG00000019947 | | *Arid5b* | | 2.010 | 0.000 | 0.000 | | [102] |
| ENSMUSG00000039217 | | *Il18* | | -4.527 | 0.000 | 0.000 | | [103] |
| ENSMUSG00000038508 | | *Gdf15* | | 3.277 | 0.000 | 0.000 | | [104] |
| ENSMUSG00000002602 | | *Axl* | | -6.006 | 0.000 | 0.000 | | [105] |
| ENSMUSG00000031639 | | *Tlr3* | | -7.830 | 0.000 | 0.000 | | [106] |
| ENSMUSG00000021367 | | *Edn1* | | 6.089 | 0.000 | 0.000 | | [107] |
| ENSMUSG00000020838 | | *Slc6a4* | | -3.319 | 0.000 | 0.000 | | [108] |
| ENSMUSG00000028341 | | *Nr4a3* | | 6.235 | 0.000 | 0.000 | | [109] |
| ENSMUSG00000032066 | | *Bco2* | | -3.424 | 0.000 | 0.000 | | [110] |
| ENSMUSG00000006445 | | *Epha2* | | 2.095 | 0.000 | 0.000 | | [111] |
| ENSMUSG00000025036 | | *Sfxn2* | | -2.745 | 0.000 | 0.000 | | [112] |
| ENSMUSG00000023034 | | *Nr4a1* | | 2.696 | 0.000 | 0.000 | | [113] |
| ENSMUSG00000025076 | | *Casp7* | | -3.026 | 0.000 | 0.000 | | [114] |
| ENSMUSG00000027360 | | *Hdc* | | -3.293 | 0.000 | 0.000 | | [115] |
| ENSMUSG00000022346 | | *Myc* | | 2.508 | 0.000 | 0.000 | | [116] |
| ENSMUSG00000059743 | | *Fdps* | | -3.804 | 0.000 | 0.000 | | [117] |
| ENSMUSG00000048806 | | *Ifnb1* | | -5.875 | 0.000 | 0.000 | | [118] |
| ENSMUSG00000028064 | | *Sema4a* | | -2.485 | 0.000 | 0.000 | | [119] |
| ENSMUSG00000044701 | | *Il27* | | -5.141 | 0.000 | 0.000 | | [120] |
| ENSMUSG00000020593 | | *Lpin1* | | -2.803 | 0.000 | 0.000 | | [121] |
| ENSMUSG00000039304 | | *Tnfsf10* | | -6.820 | 0.000 | 0.000 | | [122, 123] |
| ENSMUSG00000036353 | | *P2ry12* | | -4.602 | 0.000 | 0.000 | | [2] |
| ENSMUSG00000053846 | | *Lipg* | | -9.920 | 0.000 | 0.000 | | [124] |
| ENSMUSG00000051682 | | *Treml4* | | 2.733 | 0.000 | 0.000 | | [125] |
| ENSMUSG00000029361 | | *Nos1* | | 3.387 | 0.000 | 0.000 | | [126] |
| ENSMUSG00000071713 | | *Csf2rb* | | -2.292 | 0.000 | 0.000 | | [127] |
| ENSMUSG00000055994 | | *Nod2* | | -2.057 | 0.000 | 0.000 | | [128] |
| ENSMUSG00000026365 | | *Cfh* | | -2.898 | 0.000 | 0.000 | | [129] |
| ENSMUSG00000070385 | | *Ampd1* | | 3.055 | 0.000 | 0.000 | | [130] |
| ENSMUSG00000006611 | | *Hfe* | | -3.216 | 0.000 | 0.000 | | [131] |
| ENSMUSG00000027646 | | *Src* | | 3.244 | 0.000 | 0.000 | | [132] |
| ENSMUSG00000034394 | | *Lif* | | 3.744 | 0.000 | 0.000 | | [133] |
| ENSMUSG00000022548 | | *Apod* | | -9.398 | 0.000 | 0.000 | | [134] |
| ENSMUSG00000025880 | | *Smad7* | | 2.731 | 0.000 | 0.000 | | [135] |
| ENSMUSG00000010051 | | *Hyal1* | | 2.130 | 0.000 | 0.000 | | [136] |
| ENSMUSG00000038037 | | *Socs1* | | -2.085 | 0.000 | 0.000 | | [137] |
| ENSMUSG00000032238 | | *Rora* | | 3.546 | 0.000 | 0.000 | | [138] |
| ENSMUSG00000036362 | | *P2ry13* | | -5.457 | 0.000 | 0.000 | | [139] |
| ENSMUSG00000001930 | | *Vwf* | | 4.452 | 0.000 | 0.000 | | [140] |
| ENSMUSG00000037820 | | *Tgm2* | | 6.882 | 0.000 | 0.000 | | [141] |
| ENSMUSG00000037411 | | *Serpine1* | | 4.053 | 0.000 | 0.000 | | [142] |
| ENSMUSG00000031955 | | *Bcar1* | | 3.024 | 0.000 | 0.000 | | [143] |
| ENSMUSG00000028111 | | *Ctsk* | | 2.716 | 0.000 | 0.000 | | [144] |
| ENSMUSG00000068740 | | *Celsr2* | | 2.660 | 0.000 | 0.000 | | [145] |
| ENSMUSG00000031266 | | *Gla* | | 2.466 | 0.000 | 0.000 | | [146] |
| ENSMUSG00000026321 | | *Tnfrsf11a* | | -2.930 | 0.000 | 0.000 | | [147] |
| ENSMUSG00000022912 | | *Pros1* | | -2.391 | 0.000 | 0.000 | | [105] |
| ENSMUSG00000001946 | | *Esam* | | 2.710 | 0.000 | 0.000 | | [105] |
| ENSMUSG00000000682 | | *Cd52* | | -2.685 | 0.000 | 0.000 | | [148] |
| ENSMUSG00000020620 | | *Abca8b* | | 3.692 | 0.000 | 0.000 | | [149] |
| ENSMUSG00000033446 | | *Lpar6* | | -2.072 | 0.000 | 0.000 | | [150] |
| ENSMUSG00000015243 | | *Abca1* | | -2.919 | 0.000 | 0.000 | | [70, 97] |
| ENSMUSG00000003379 | | *Cd79a* | | 3.590 | 0.000 | 0.000 | | [151] |
| ENSMUSG00000001020 | | *S100a4* | | -2.233 | 0.000 | 0.000 | | [152] |
| ENSMUSG00000020892 | | *Aloxe3* | | 4.224 | 0.000 | 0.000 | | [153] |
| ENSMUSG00000025804 | | *Ccr1* | | 2.638 | 0.000 | 0.000 | | [154] |
| ENSMUSG00000026921 | | *Egfl7* | | -2.728 | 0.000 | 0.000 | | [155] |
| ENSMUSG00000005824 | | *Tnfsf14* | | 2.536 | 0.000 | 0.000 | | [156] |
| ENSMUSG00000029925 | | *Tbxas1* | | -3.739 | 0.000 | 0.000 | | [157] |
| ENSMUSG00000035373 | | *Ccl7* | | 3.174 | 0.000 | 0.000 | | [158] |
| ENSMUSG00000049608 | | *Gpr55* | | -2.882 | 0.000 | 0.000 | | [159] |
| ENSMUSG00000024391 | | *Apom* | | 2.025 | 0.000 | 0.000 | | [160] |
| ENSMUSG00000042745 | | *Id1* | | 2.924 | 0.000 | 0.000 | | [161] |
| ENSMUSG00000031637 | | *Lrp2bp* | | 2.760 | 0.000 | 0.000 | | [162] |
| ENSMUSG00000020447 | | *Npc1l1* | | 2.591 | 0.000 | 0.000 | | [163] |
| ENSMUSG00000030046 | | *Bmp10* | | -4.576 | 0.000 | 0.000 | | [164] |
| ENSMUSG00000027318 | | *Adam33* | | -4.269 | 0.000 | 0.000 | | [165] |
| ENSMUSG00000015966 | | *Il17rb* | | 2.277 | 0.000 | 0.000 | | [166] |
| ENSMUSG00000031896 | | *Ctrl* | | -3.040 | 0.000 | 0.000 | | [167] |
| ENSMUSG00000042784 | | *Muc1* | | 2.860 | 0.000 | 0.000 | | [168] |
| ENSMUSG00000019987 | | *Arg1* | | 2.207 | 0.000 | 0.000 | | [169] |
| ENSMUSG00000003849 | | *Nqo1* | | 2.211 | 0.000 | 0.000 | | [170] |
| ENSMUSG00000062585 | | *Cnr2* | | -2.258 | 0.000 | 0.000 | | [171] |
| ENSMUSG00000052013 | | *Btla* | | -4.449 | 0.000 | 0.000 | | [172] |
| ENSMUSG00000030156 | | *Cd69* | | -3.662 | 0.000 | 0.000 | | [173] |
| ENSMUSG00000001663 | | *Gstt1* | | -3.958 | 0.000 | 0.000 | | [174] |
| ENSMUSG00000045382 | | *Cxcr4* | | 3.737 | 0.000 | 0.000 | | [175] |
| ENSMUSG00000037542 | | *Aldh8a1* | | 4.155 | 0.000 | 0.000 | | [176] |
| ENSMUSG00000031078 | | *Cttn* | | 2.599 | 0.000 | 0.000 | | [177] |
| ENSMUSG00000027204 | | *Fbn1* | | -6.851 | 0.000 | 0.000 | | [178] |
| ENSMUSG00000033377 | | *Palmd* | | 4.104 | 0.000 | 0.000 | | [179] |
| ENSMUSG00000047959 | | *Kcna3* | | -6.441 | 0.000 | 0.000 | | [180] |
| ENSMUSG00000005320 | | *Fgfr4* | | 2.794 | 0.000 | 0.000 | | [181] |
| ENSMUSG00000038179 | | *Slamf7* | | 3.566 | 0.000 | 0.000 | | [182] |
| ENSMUSG00000034881 | | *Tbxa2r* | | 2.872 | 0.000 | 0.000 | | [183] |
| ENSMUSG00000074037 | | *Mc1r* | | 3.442 | 0.000 | 0.000 | | [184] |
| ENSMUSG00000056671 | | *Prelid2* | | 2.186 | 0.000 | 0.000 | | [185] |
| ENSMUSG00000024793 | | *Tnfrsf25* | | -2.497 | 0.000 | 0.000 | | [186] |
| ENSMUSG00000009350 | | *Mpo* | | 4.619 | 0.000 | 0.000 | | [187] |
| ENSMUSG00000039521 | | *Foxp3* | | 2.394 | 0.000 | 0.000 | | [188] |
| ENSMUSG00000030324 | | *Rho* | | 3.921 | 0.000 | 0.000 | | [189] |
| ENSMUSG00000030223 | | *Ptpro* | | -2.456 | 0.000 | 0.000 | | [190] |
| ENSMUSG00000028427 | | *Aqp7* | | 2.403 | 0.000 | 0.000 | | [191] |
| ENSMUSG00000053469 | | *Tg* | | 2.135 | 0.000 | 0.000 | | [192] |
| ENSMUSG00000061878 | | *Sphk1* | | 2.802 | 0.000 | 0.000 | | [193] |
| ENSMUSG00000079164 | | *Tlr5* | | -6.389 | 0.000 | 0.000 | | [194] |
| ENSMUSG00000102037 | | *Bcl2a1a* | | -2.348 | 0.000 | 0.000 | | [195] |
| ENSMUSG00000031766 | | *Slc12a3* | | 4.223 | 0.000 | 0.000 | | [196] |
| ENSMUSG00000032083 | | *Apoa1* | | 3.381 | 0.000 | 0.000 | | [70, 97] |
| ENSMUSG00000027398 | | *Il1b* | | -2.520 | 0.000 | 0.000 | | [97, 197] |
| ENSMUSG00000025754 | | *Agbl1* | | -6.047 | 0.000 | 0.000 | | [198] |
| ENSMUSG00000039193 | | *Nlrc4* | | -2.291 | 0.000 | 0.000 | | [199] |
| ENSMUSG00000030895 | | *Hpx* | | 3.125 | 0.000 | 0.000 | | [200] |
| ENSMUSG00000026009 | | *Icos* | | -3.397 | 0.000 | 0.000 | | [201] |
| ENSMUSG00000041548 | | *Hspb8* | | 3.592 | 0.000 | 0.000 | | [202] |
| ENSMUSG00000051136 | | *Ghsr* | | 4.322 | 0.000 | 0.000 | | [203] |
| ENSMUSG00000029859 | | *Epha1* | | 4.265 | 0.000 | 0.000 | | [204] |
| ENSMUSG00000016529 | | *Il10* | | 3.695 | 0.000 | 0.000 | | [205] |
| ENSMUSG00000030124 | | *Lag3* | | 2.867 | 0.000 | 0.000 | | [206] |
| ENSMUSG00000027070 | | *Lrp2* | | 3.538 | 0.000 | 0.000 | | [207] |
| ENSMUSG00000041460 | | *Cacna2d4* | | -2.800 | 0.000 | 0.000 | | [208] |
| ENSMUSG00000040592 | | *Cd79b* | | 4.147 | 0.000 | 0.000 | | [209] |
| ENSMUSG00000004698 | | *Hdac9* | | -2.759 | 0.000 | 0.000 | | [210] |
| ENSMUSG00000026700 | | *Tnfsf4* | | 2.197 | 0.000 | 0.000 | | [211] |
| ENSMUSG00000014813 | | *Stc1* | | -3.570 | 0.000 | 0.000 | | [212] |
| ENSMUSG00000002289 | | *Angptl4* | | -2.203 | 0.000 | 0.000 | | [213] |
| ENSMUSG00000091971 | | *Hspa1a* | | 5.484 | 0.000 | 0.000 | | [214] |
| ENSMUSG00000023914 | | *Mep1a* | | 5.484 | 0.000 | 0.000 | | [215] |
| ENSMUSG00000020122 | | *Egfr* | | 2.977 | 0.000 | 0.000 | | [216] |
| ENSMUSG00000018916 | | *Csf2* | | -3.237 | 0.000 | 0.000 | | [217] |
| ENSMUSG00000022683 | | *Pla2g10* | | 2.815 | 0.000 | 0.000 | | [218] |
| ENSMUSG00000003665 | | *Has1* | | 3.300 | 0.000 | 0.000 | | [219] |
| ENSMUSG00000020902 | | *Ntn1* | | 2.830 | 0.000 | 0.000 | | [220] |
| ENSMUSG00000029530 | | *Ccr9* | | 2.000 | 0.000 | 0.000 | | [221] |
| ENSMUSG00000057530 | | *Ece1* | | -2.578 | 0.000 | 0.000 | | [222] |
| ENSMUSG00000002996 | | *Hbp1* | | 2.218 | 0.000 | 0.000 | | [223] |
| ENSMUSG00000020808 | | *Pimreg* | | -2.363 | 0.000 | 0.000 | | [224] |
| ENSMUSG00000042333 | | *Tnfrsf14* | | -5.594 | 0.000 | 0.000 | | [225] |
| ENSMUSG00000026069 | | *Il1rl1* | | -2.299 | 0.000 | 0.000 | | [226] |
| ENSMUSG00000035448 | | *Ccr3* | | 2.976 | 0.000 | 0.001 | | [227] |
| ENSMUSG00000063903 | | *Klk1* | | 5.296 | 0.000 | 0.001 | | [228] |
| ENSMUSG00000028874 | | *Fgr* | | -2.734 | 0.000 | 0.001 | | [229] |
| ENSMUSG00000050440 | | *Hamp* | | 3.801 | 0.001 | 0.001 | | [230] |
| ENSMUSG00000040329 | | *Il7* | | -2.597 | 0.001 | 0.002 | | [231] |
| ENSMUSG00000028763 | | *Hspg2* | | 2.661 | 0.001 | 0.002 | | [232] |
| ENSMUSG00000012705 | | *Retn* | | 5.079 | 0.001 | 0.004 | | [233] |
| ENSMUSG00000026117 | | *Zap70* | | 2.727 | 0.002 | 0.004 | | [234] |
| ENSMUSG00000050395 | | *Tnfsf15* | | -4.887 | 0.002 | 0.005 | | [235] |
| ENSMUSG00000015854 | | *Cd5l* | | -2.886 | 0.002 | 0.006 | | [236] |
| ENSMUSG00000028661 | | *Epha8* | | 3.018 | 0.002 | 0.006 | | [204] |
| ENSMUSG00000104713 | | *Gbp6* | | -3.328 | 0.003 | 0.006 | | [237] |
| ENSMUSG00000029379 | | *Cxcl3* | | 3.447 | 0.003 | 0.008 | | [238, 239] |
| ENSMUSG00000027460 | | *Angpt4* | | -2.806 | 0.004 | 0.008 | | [240] |
| ENSMUSG00000028602 | | *Tnfrsf8* | | 2.936 | 0.004 | 0.008 | | [241] |
| ENSMUSG00000026070 | | *Il18r1* | | -2.041 | 0.004 | 0.009 | | [242] |
| ENSMUSG00000026866 | | *Kynu* | | -4.760 | 0.004 | 0.010 | | [243] |
| ENSMUSG00000057378 | | *Ryr3* | | 4.825 | 0.004 | 0.010 | | [244] |
| ENSMUSG00000063415 | | *Cyp26b1* | | 4.825 | 0.004 | 0.010 | | [245] |
| ENSMUSG00000030162 | | *Olr1* | | 4.825 | 0.004 | 0.010 | | [70] |
| ENSMUSG00000038146 | | *Notch3* | | 4.825 | 0.004 | 0.010 | | [246] |
| ENSMUSG00000015355 | | *Cd48* | | -2.081 | 0.005 | 0.012 | | [246] |
| ENSMUSG00000033208 | | *S100b* | | 2.002 | 0.005 | 0.012 | | [247] |
| ENSMUSG00000050578 | | *Mmp13* | | 2.289 | 0.006 | 0.013 | | [248] |
| ENSMUSG00000014361 | | *Mertk* | | 4.678 | 0.007 | 0.016 | | [249] |
| ENSMUSG00000031994 | | *Adamts8* | | 4.679 | 0.007 | 0.016 | | [250] |
| ENSMUSG00000022816 | | *Fstl1* | | 4.679 | 0.007 | 0.016 | | [251] |
| ENSMUSG00000040899 | | *Ccr6* | | 4.677 | 0.007 | 0.016 | | [252, 253] |
| ENSMUSG00000039153 | | *Runx2* | | 4.677 | 0.007 | 0.016 | | [254] |
| ENSMUSG00000033805 | | *Ephx4* | | 2.344 | 0.007 | 0.017 | | [255] |
| ENSMUSG00000040505 | | *Abcg5* | | 2.758 | 0.008 | 0.018 | | [256] |
| ENSMUSG00000036856 | | *Wnt4* | | 3.231 | 0.008 | 0.019 | | [257] |
| ENSMUSG00000030278 | | *Cidec* | | 2.212 | 0.012 | 0.026 | | [258] |
| ENSMUSG00000031503 | | *Col4a2* | | 2.346 | 0.012 | 0.027 | | [259] |
| ENSMUSG00000075602 | | *Ly6a* | | -4.451 | 0.013 | 0.027 | | [260] |
| ENSMUSG00000022483 | | *Col2a1* | | 4.515 | 0.013 | 0.027 | | [261] |
| ENSMUSG00000020963 | | *Tshr* | | 2.344 | 0.015 | 0.031 | | [262] |
| ENSMUSG00000022504 | | *Ciita* | | 2.561 | 0.018 | 0.038 | | [263] |
| ENSMUSG00000007888 | | *Crlf1* | | 2.157 | 0.020 | 0.042 | | [77] |
| ENSMUSG00000022382 | | *Wnt7b* | | -2.857 | 0.022 | 0.045 | | [264] |
| ENSMUSG00000021388 | | *Aspn* | | 2.975 | 0.022 | 0.045 | | [265] |
| ENSMUSG00000023057 | | *Fabp2* | | 4.332 | 0.023 | 0.047 | | [266] |
| ENSMUSG00000029380 | | *Cxcl1* | | 4.332 | 0.023 | 0.047 | | [267] |
| ENSMUSG00000089678 | | *Agxt2* | | 4.331 | 0.023 | 0.047 | | [268] |
| ENSMUSG00000039672 | | *Kcne2* | | -4.260 | 0.023 | 0.047 | | [269] |

# 0.000 means <0.0001.

**REFERENCES**

1. Xiong Y, Yepuri G, Forbiteh M, Yu Y, Montani JP, Yang Z, Ming XF. ARG2 impairs endothelial autophagy through regulation of MTOR and PRKAA/AMPK signaling in advanced atherosclerosis. Autophagy. 2014; 10:2223-38.

<https://doi.org/10.4161/15548627.2014.981789>

[PMID:25484082](https://www.ncbi.nlm.nih.gov/pubmed/25484082)

2. Pi S, Mao L, Chen J, Shi H, Liu Y, Guo X, Li Y, Zhou L, He H, Yu C, Liu J, Dang Y, Xia Y, et al. The P2RY12 receptor promotes VSMC-derived foam cell formation by inhibiting autophagy in advanced atherosclerosis. Autophagy. 2020. [Epub ahead of print].

<https://doi.org/10.1080/15548627.2020.1741202>

[PMID:32160082](https://www.ncbi.nlm.nih.gov/pubmed/32160082)

3. Krane M, Dummler S, Dressen M, Hauner H, Hoffmann M, Haller D, Heller K, Wildhirt S, Voss B, Grammer J, Lahm H, Lange R, Bauernschmitt R. Identification of an up-regulated anti-apoptotic network in the internal thoracic artery. Int J Cardiol. 2011; 149:221-26.

<https://doi.org/10.1016/j.ijcard.2010.02.003>

[PMID:20207035](https://www.ncbi.nlm.nih.gov/pubmed/20207035)

4. Okazaki S, Morimoto T, Kamatani Y, Kamimura T, Kobayashi H, Harada K, Tomita T, Higashiyama A, Takahashi JC, Nakagawara J, Koga M, Toyoda K, Washida K, et al. Moyamoya Disease Susceptibility Variant RNF213 p.R4810K Increases the Risk of Ischemic Stroke Attributable to Large-Artery Atherosclerosis. Circulation. 2019; 139:295-98.

<https://doi.org/10.1161/CIRCULATIONAHA.118.038439>

[PMID:30615506](https://www.ncbi.nlm.nih.gov/pubmed/30615506)

5. Jeong SJ, Kim S, Park JG, Jung IH, Lee MN, Jeon S, Kweon HY, Yu DY, Lee SH, Jang Y, Kang SW, Han KH, Miller YI, et al. Prdx1 (peroxiredoxin 1) deficiency reduces cholesterol efflux via impaired macrophage lipophagic flux. Autophagy. 2018; 14:120-33.

<https://doi.org/10.1080/15548627.2017.1327942>

[PMID:28605287](https://www.ncbi.nlm.nih.gov/pubmed/28605287)

6. Liu Y, Carmona-Rivera C, Moore E, Seto NL, Knight JS, Pryor M, Yang ZH, Hemmers S, Remaley AT, Mowen KA, Kaplan MJ. Myeloid-Specific Deletion of Peptidylarginine Deiminase 4 Mitigates Atherosclerosis. Front Immunol. 2018; 9:1680.

<https://doi.org/10.3389/fimmu.2018.01680>

[PMID:30140264](https://www.ncbi.nlm.nih.gov/pubmed/30140264)

7. Liu Z, Han Y, Li L, Lu H, Meng G, Li X, Shirhan M, Peh MT, Xie L, Zhou S, Wang X, Chen Q, Dai W, et al. The hydrogen sulfide donor, GYY4137, exhibits anti-atherosclerotic activity in high fat fed apolipoprotein E(-/-) mice. Br J Pharmacol. 2013; 169:1795-809.

<https://doi.org/10.1111/bph.12246>

[PMID:23713790](https://www.ncbi.nlm.nih.gov/pubmed/23713790)

8. Wang W, Zhang ZZ, Wu Y, Wang RQ, Chen JW, Chen J, Zhang Y, Chen YJ, Geng M, Xu ZD, Dai M, Li JH, Pan LL. Corrigendum: (-)-Epigallocatechin-3-Gallate Ameliorates Atherosclerosis and Modulates Hepatic Lipid Metabolic Gene Expression in Apolipoprotein E Knockout Mice: Involvement of TTC39B. Front Pharmacol. 2018; 9:459.

<https://doi.org/10.3389/fphar.2018.00459>

[PMID:29745376](https://www.ncbi.nlm.nih.gov/pubmed/29745376)

9. Jung TW, Park HS, Jeong JH, Lee T. Salsalate ameliorates the atherosclerotic response through HO-1- and SIRT1-mediated suppression of ER stress and inflammation. Inflamm Res. 2019; 68:655-663.

<https://doi.org/10.1007/s00011-019-01248-6>

PMID:[31143972](https://pubmed.ncbi.nlm.nih.gov/31143972)

10. Kishimoto Y, Sasaki K, Saita E, Niki H, Ohmori R, Kondo K, Momiyama Y. Plasma Heme Oxygenase-1 Levels and Carotid Atherosclerosis. Stroke. 2018; 49:2230-32.

<https://doi.org/10.1161/STROKEAHA.118.022256>

[PMID:30354985](https://www.ncbi.nlm.nih.gov/pubmed/30354985)

11. Wei Y, Zhu M, Corbalan-Campos J, Heyll K, Weber C, Schober A. Regulation of Csf1r and Bcl6 in macrophages mediates the stage-specific effects of microRNA-155 on atherosclerosis. Arterioscler Thromb Vasc Biol. 2015; 35:796-803.

<https://doi.org/10.1161/ATVBAHA.114.304723>

[PMID:25810298](https://www.ncbi.nlm.nih.gov/pubmed/25810298)

12. Chang HR, Josefs T, Scerbo D, Gumaste N, Hu Y, Huggins LA, Barrett TJ, Chiang SS, Grossman J, Bagdasarov S, Fisher EA, Goldberg IJ. Role of LpL (Lipoprotein Lipase) in Macrophage Polarization In Vitro and In Vivo. Arterioscler Thromb Vasc Biol. 2019; 39:1967-85.

<https://doi.org/10.1161/ATVBAHA.119.312389>

[PMID:31434492](https://www.ncbi.nlm.nih.gov/pubmed/31434492)

13. Ferronato S, Scuro A, Gomez-Lira M, Mazzucco S, Olivato S, Turco A, Elisa O, Malerba G, Romanelli MG. Correlations between gene expression highlight a different activation of ACE/TLR4/PTGS2 signaling in symptomatic and asymptomatic plaques in atherosclerotic patients. Mol Biol Rep. 2018; 45:657-62.

<https://doi.org/10.1007/s11033-018-4207-7>

[PMID:29923152](https://www.ncbi.nlm.nih.gov/pubmed/29923152)

14. Hammad SM, Twal WO, Barth JL, Smith KJ, Saad AF, Virella G, Argraves WS, Lopes-Virella MF. Oxidized LDL immune complexes and oxidized LDL differentially affect the expression of genes involved with inflammation and survival in human U937 monocytic cells. Atherosclerosis. 2009; 202:394-404.

<https://doi.org/10.1016/j.atherosclerosis.2008.05.032>

[PMID:18597759](https://www.ncbi.nlm.nih.gov/pubmed/18597759)

15. Callegari A, Liu Y, White CC, Chait A, Gough P, Raines EW, Cox D, Kavanagh TJ, Rosenfeld ME. Gain and loss of function for glutathione synthesis: impact on advanced atherosclerosis in apolipoprotein E-deficient mice. Arterioscler Thromb Vasc Biol. 2011; 31:2473-82.

<https://doi.org/10.1161/ATVBAHA.111.229765>

[PMID:21868708](https://www.ncbi.nlm.nih.gov/pubmed/21868708)

16. Ma F, Li T, Zhang H, Wu G. MiR-30s Family Inhibit the Proliferation and Apoptosis in Human Coronary Artery Endothelial Cells Through Targeting the 3'UTR Region of ITGA4 and PLCG1. J Cardiovasc Pharmacol. 2016; 68:327-33.

<https://doi.org/10.1097/FJC.0000000000000419>

[PMID:27464494](https://www.ncbi.nlm.nih.gov/pubmed/27464494)

17. Iwata H, Goettsch C, Sharma A, Ricchiuto P, Goh WW, Halu A, Yamada I, Yoshida H, Hara T, Wei M, Inoue N, Fukuda D, Mojcher A, et al. PARP9 and PARP14 cross-regulate macrophage activation via STAT1 ADP-ribosylation. Nat Commun. 2016; 7:12849.

<https://doi.org/10.1038/ncomms12849>

[PMID:27796300](https://www.ncbi.nlm.nih.gov/pubmed/27796300)

18. Lagor WR, Fields DW, Bauer RC, Crawford A, Abt MC, Artis D, Wherry EJ, Rader DJ. Genetic manipulation of the ApoF/Stat2 locus supports an important role for type I interferon signaling in atherosclerosis. Atherosclerosis. 2014; 233:234-41.

<https://doi.org/10.1016/j.atherosclerosis.2013.12.043>

[PMID:24529150](https://www.ncbi.nlm.nih.gov/pubmed/24529150)

19. Giuliani C. The Flavonoid Quercetin Induces AP-1 Activation in FRTL-5 Thyroid Cells. Antioxidants. 2019; 8:E112.

<https://doi.org/10.3390/antiox8050112>

[PMID:31035637](https://www.ncbi.nlm.nih.gov/pubmed/31035637)

20. Nai W, Threapleton D, Lu J, Zhang K, Wu H, Fu Y, Wang Y, Ou Z, Shan L, Ding Y, Yu Y, Dai M. Identification of novel genes and pathways in carotid atheroma using integrated bioinformatic methods. Sci Rep. 2016; 6:18764.

<https://doi.org/10.1038/srep18764>

[PMID:26742467](https://www.ncbi.nlm.nih.gov/pubmed/26742467)

21. Zawada AM, Rogacev KS, Rotter B, Winter P, Marell RR, Fliser D, Heine GH. SuperSAGE evidence for CD14++CD16+ monocytes as a third monocyte subset. Blood. 2011; 118:e50-61.

<https://doi.org/10.1182/blood-2011-01-326827>

[PMID:21803849](https://www.ncbi.nlm.nih.gov/pubmed/21803849)

22. Li W, Xin B, Yan J, Wu Y, Hu B, Liu L, Wang Y, Ahn J, Skowronski J, Zhang Z, Wang Y, Wang H. SAMHD1 Gene Mutations Are Associated with Cerebral Large-Artery Atherosclerosis. BioMed Res Int. 2015; 2015:739586.

<https://doi.org/10.1155/2015/739586>

[PMID:26504826](https://www.ncbi.nlm.nih.gov/pubmed/26504826)

23. Wang M, Li L, Liu R, Song Y, Zhang X, Niu W, Kumar AK, Guo Z, Hu Z. Obesity-induced overexpression of miRNA-24 regulates cholesterol uptake and lipid metabolism by targeting SR-B1. Gene. 2018; 668:196-203.

<https://doi.org/10.1016/j.gene.2018.05.072>

[PMID:29787826](https://www.ncbi.nlm.nih.gov/pubmed/29787826)

24. Clement M, Basatemur G, Masters L, Baker L, Bruneval P, Iwawaki T, Kneilling M, Yamasaki S, Goodall J, Mallat Z. Necrotic Cell Sensor Clec4e Promotes a Proatherogenic Macrophage Phenotype Through Activation of the Unfolded Protein Response. Circulation. 2016; 134:1039-51.

<https://doi.org/10.1161/CIRCULATIONAHA.116.022668>

[PMID:27587433](https://www.ncbi.nlm.nih.gov/pubmed/27587433)

25. Huang C, Lewis C, Borg NA, Canals M, Diep H, Drummond GR, Goode RJ, Schittenhelm RB, Vinh A, Zhu M, Kemp-Harper B, Kleifeld O, Stone MJ. Proteomic Identification of Interferon-Induced Proteins with Tetratricopeptide Repeats as Markers of M1 Macrophage Polarization. J Proteome Res. 2018; 17:1485-99.

<https://doi.org/10.1021/acs.jproteome.7b00828>

[PMID:29508616](https://www.ncbi.nlm.nih.gov/pubmed/29508616)

26. Sorrentino R, Morello S, Chen S, Bonavita E, Pinto A. The activation of liver X receptors inhibits toll-like receptor-9-induced foam cell formation. J Cell Physiol. 2010; 223:158-67.

<https://doi.org/10.1002/jcp.22022>

[PMID:20049870](https://www.ncbi.nlm.nih.gov/pubmed/20049870)

27. Gromovsky AD, Schugar RC, Brown AL, Helsley RN, Burrows AC, Ferguson D, Zhang R, Sansbury BE, Lee RG, Morton RE, Allende DS, Parks JS, Spite M, Brown JM. ?-5 Fatty Acid Desaturase *FADS1* Impacts Metabolic Disease by Balancing Proinflammatory and Proresolving Lipid Mediators. Arterioscler Thromb Vasc Biol. 2018; 38:218-31.

<https://doi.org/10.1161/ATVBAHA.117.309660>

[PMID:29074585](https://www.ncbi.nlm.nih.gov/pubmed/29074585)

28. Worrall BB, Azhar S, Nyquist PA, Ackerman RH, Hamm TL, DeGraba TJ. Interleukin-1 receptor antagonist gene polymorphisms in carotid atherosclerosis. Stroke. 2003; 34:790-93.

<https://doi.org/10.1161/01.STR.0000057815.79289.EC>

[PMID:12624309](https://www.ncbi.nlm.nih.gov/pubmed/12624309)

29. Islam Z, Inui T, Ishibashi O. Gpr137b is an orphan G-protein-coupled receptor associated with M2 macrophage polarization. Biochem Biophys Res Commun. 2019; 509:657-63.

<https://doi.org/10.1016/j.bbrc.2018.12.140>

[PMID:30595385](https://www.ncbi.nlm.nih.gov/pubmed/30595385)

30. Yang L, Yang F, Zhao H, Wang M, Zhang Y. Circular RNA circCHFR Facilitates the Proliferation and Migration of Vascular Smooth Muscle via miR-370/FOXO1/Cyclin D1 Pathway. Mol Ther Nucleic Acids. 2019; 16:434-41.

<https://doi.org/10.1016/j.omtn.2019.02.028>

[PMID:31048182](https://www.ncbi.nlm.nih.gov/pubmed/31048182)

31. Li TP, Sun SW, Xiong GZ, Qiu F, Yang DM, Sun SY, Xie XJ, Liao DF, Chen JX, Tuo QH. Direct Interaction of Daxx and Androgen Receptor Is Required for Their Regulatory Activity in Cholesterol Biosynthesis. Pharmacology. 2020 [Epub ahead of print].

<https://doi.org/10.1159/000506488>

[PMID:32694250](https://www.ncbi.nlm.nih.gov/pubmed/32694250)

32. Harada N, Ito K, Hosoya T, Mimura J, Maruyama A, Noguchi N, Yagami K, Morito N, Takahashi S, Maher JM, Yamamoto M, Itoh K. Nrf2 in bone marrow-derived cells positively contributes to the advanced stage of atherosclerotic plaque formation. Free Radic Biol Med. 2012; 53:2256-62.

<https://doi.org/10.1016/j.freeradbiomed.2012.10.001>

[PMID:23051009](https://www.ncbi.nlm.nih.gov/pubmed/23051009)

33. Liu Y, Zhong Y, Chen H, Wang D, Wang M, Ou JS, Xia M. Retinol-Binding Protein-Dependent Cholesterol Uptake Regulates Macrophage Foam Cell Formation and Promotes Atherosclerosis. Circulation. 2017; 135:1339-54.

<https://doi.org/10.1161/CIRCULATIONAHA.116.024503>

[PMID:28122883](https://www.ncbi.nlm.nih.gov/pubmed/28122883)

34. Shewale SV, Boudyguina E, Zhu X, Shen L, Hutchins PM, Barkley RM, Murphy RC, Parks JS. Botanical oils enriched in n-6 and n-3 FADS2 products are equally effective in preventing atherosclerosis and fatty liver. J Lipid Res. 2015; 56:1191-205.

<https://doi.org/10.1194/jlr.M059170>

[PMID:25921305](https://www.ncbi.nlm.nih.gov/pubmed/25921305)

35. Gopoju R, Panangipalli S, Kotamraju S. Metformin treatment prevents SREBP2-mediated cholesterol uptake and improves lipid homeostasis during oxidative stress-induced atherosclerosis. Free Radic Biol Med. 2018; 118:85-97.

<https://doi.org/10.1016/j.freeradbiomed.2018.02.031>

[PMID:29499335](https://www.ncbi.nlm.nih.gov/pubmed/29499335)

36. Ahrens I, Domeij H, Eisenhardt SU, Topcic D, Albrecht M, Leitner E, Viitaniemi K, Jowett JB, Lappas M, Bode C, Haviv I, Peter K. Opposing effects of monomeric and pentameric C-reactive protein on endothelial progenitor cells. Basic Res Cardiol. 2011; 106:879-95.

<https://doi.org/10.1007/s00395-011-0191-y>

[PMID:21562922](https://www.ncbi.nlm.nih.gov/pubmed/21562922)

37. Saliba-Gustafsson P, Pedrelli M, Gertow K, Werngren O, Janas V, Pourteymour S, Baldassarre D, Tremoli E, Veglia F, Rauramaa R, Smit AJ, Giral P, Kurl S, et al, and IMPROVE Study Group. Subclinical atherosclerosis and its progression are modulated by PLIN2 through a feed-forward loop between LXR and autophagy. J Intern Med. 2019; 286:660-75.

<https://doi.org/10.1111/joim.12951>

[PMID:31251843](https://www.ncbi.nlm.nih.gov/pubmed/31251843)

38. Douglas G, Bendall JK, Crabtree MJ, Tatham AL, Carter EE, Hale AB, Channon KM. Endothelial-specific Nox2 overexpression increases vascular superoxide and macrophage recruitment in ApoE?/? mice. Cardiovasc Res. 2012; 94:20-29.

<https://doi.org/10.1093/cvr/cvs026>

[PMID:22287576](https://www.ncbi.nlm.nih.gov/pubmed/22287576)

39. Xia F, Li R, Wang C, Yang S, Tian L, Dong H, Pei C, He S, Jiang P, Cheng H, Fang S, Li H, Xu H. IRGM1 regulates oxidized LDL uptake by macrophage via actin-dependent receptor internalization during atherosclerosis. Sci Rep. 2013; 3:1867.

<https://doi.org/10.1038/srep01867>

[PMID:23689639](https://www.ncbi.nlm.nih.gov/pubmed/23689639)

40. Karunagaran S, Kavitha R, Vadivelu M, Lee KW, Meganathan C. Insight Mechanism of the Selective Lanosterol Synthase Inhibitor: Molecular Modeling, Docking and Density Functional Theory Approaches. Curr Comput Aided Drug Des. 2017; 13:275-93.

<https://doi.org/10.2174/1573409913666170426153509>

[PMID:28462697](https://www.ncbi.nlm.nih.gov/pubmed/28462697)

41. Xu Z, Le K, Moghadasian MH. Long-term phytosterol treatment alters gene expression in the liver of apo E-deficient mice. J Nutr Biochem. 2008; 19:545-54.

<https://doi.org/10.1016/j.jnutbio.2007.06.012>

[PMID:18155511](https://www.ncbi.nlm.nih.gov/pubmed/18155511)

42. Hong YF, Kim H, Kim HS, Park WJ, Kim JY, Chung DK. Lactobacillus acidophilus K301 Inhibits Atherogenesis via Induction of 24 (S), 25-Epoxycholesterol-Mediated ABCA1 and ABCG1 Production and Cholesterol Efflux in Macrophages. PLoS One. 2016; 11:e0154302.

<https://doi.org/10.1371/journal.pone.0154302>

[PMID:27120199](https://www.ncbi.nlm.nih.gov/pubmed/27120199)

43. Koyama S, Ohtani K, Fukuzawa J, Yao N, Fukuda M, Jang SJ, Hasebe N, Kikuchi K, Itabe H, Yoshida I, Suzuki Y, Wakamiya N. The induction of human CL-P1 expression in hypoxia/reoxygenation culture condition and rat CL-P1 after ischemic/reperfusion treatment. Biochim Biophys Acta. 2011; 1810:836-42.

<https://doi.org/10.1016/j.bbagen.2011.06.013>

[PMID:21723916](https://www.ncbi.nlm.nih.gov/pubmed/21723916)

44. Walker ME, Matthan NR, Solano-Aguilar G, Jang S, Lakshman S, Molokin A, Faits T, Urban JF Jr, Johnson WE, Lamon-Fava S, Lichtenstein AH. A Western-type dietary pattern and atorvastatin induce epicardial adipose tissue interferon signaling in the Ossabaw pig. J Nutr Biochem. 2019; 67:212-18.

<https://doi.org/10.1016/j.jnutbio.2019.02.003>

[PMID:30981985](https://www.ncbi.nlm.nih.gov/pubmed/30981985)

45. Cheng S, Zhou F, Xu Y, Liu X, Zhang Y, Gu M, Su Z, Zhao D, Zhang L, Jia Y. Geniposide regulates the miR-101/MKP-1/p38 pathway and alleviates atherosclerosis inflammatory injury in ApoE^-/-^ mice. Immunobiology. 2019; 224:296-306.

<https://doi.org/10.1016/j.imbio.2018.12.005>

[PMID:30630636](https://www.ncbi.nlm.nih.gov/pubmed/30630636)

46. Hernandez-Vargas P, Ortiz-Munoz G, Lopez-Franco O, Suzuki Y, Gallego-Delgado J, Sanjuan G, Lazaro A, Lopez-Parra V, Ortega L, Egido J, Gomez-Guerrero C. Fcgamma receptor deficiency confers protection against atherosclerosis in apolipoprotein E knockout mice. Circ Res. 2006; 99:1188-96.

<https://doi.org/10.1161/01.RES.0000250556.07796.6c>

[PMID:17053192](https://www.ncbi.nlm.nih.gov/pubmed/17053192)

47. Huang C, Yu XH, Zheng XL, Ou X, Tang CK. Interferon-stimulated gene 15 promotes cholesterol efflux by activating autophagy via the miR-17-5p/Beclin-1 pathway in THP-1 macrophage-derived foam cells. Eur J Pharmacol. 2018; 827:13-21.

<https://doi.org/10.1016/j.ejphar.2018.02.042>

[PMID:29518394](https://www.ncbi.nlm.nih.gov/pubmed/29518394)

48. Gast M, Rauch BH, Nakagawa S, Haghikia A, Jasina A, Haas J, Nath N, Jensen L, Stroux A, Bohm A, Friebel J, Rauch U, Skurk C, et al. Immune system-mediated atherosclerosis caused by deficiency of long non-coding RNA MALAT1 in ApoE-/-mice. Cardiovasc Res. 2019; 115:302-14.

<https://doi.org/10.1093/cvr/cvy202>

[PMID:30101304](https://www.ncbi.nlm.nih.gov/pubmed/30101304)

49. Szanto M, Bai P. The role of ADP-ribose metabolism in metabolic regulation, adipose tissue differentiation, and metabolism. Genes Dev. 2020; 34:321-40.

<https://doi.org/10.1101/gad.334284.119>

[PMID:32029456](https://www.ncbi.nlm.nih.gov/pubmed/32029456)

50. Shen X, Wang W, Wang L, Houde C, Wu W, Tudor M, Thompson JR, Sisk CM, Hubbard B, Li J. Identification of genes affecting apolipoprotein B secretion following siRNA-mediated gene knockdown in primary human hepatocytes. Atherosclerosis. 2012; 222:154-57.

<https://doi.org/10.1016/j.atherosclerosis.2012.02.012>

[PMID:22398276](https://www.ncbi.nlm.nih.gov/pubmed/22398276)

51. He XW, Li WL, Li C, Liu P, Shen YG, Zhu M, Jin XP. Serum levels of galectin-1, galectin-3, and galectin-9 are associated with large artery atherosclerotic stroke. Sci Rep. 2017; 7:40994.

<https://doi.org/10.1038/srep40994>

[PMID:28112232](https://www.ncbi.nlm.nih.gov/pubmed/28112232)

52. Zhang C, Qin JJ, Gong FH, Tong JJ, Cheng WL, Wang H, Zhang Y, Zhu X, She ZG, Xia H, Zhu LH. Mindin deficiency in macrophages protects against foam cell formation and atherosclerosis by targeting LXR-?. Clin Sci (Lond). 2018; 132:1199-213.

<https://doi.org/10.1042/CS20180033>

[PMID:29695588](https://www.ncbi.nlm.nih.gov/pubmed/29695588)

53. Adamson SE, Polanowska-Grabowska R, Marqueen K, Griffiths R, Angdisen J, Breevoort SR, Schulman IG, Leitinger N. Deficiency of Dab2 (Disabled Homolog 2) in Myeloid Cells Exacerbates Inflammation in Liver and Atherosclerotic Plaques in LDLR (Low-Density Lipoprotein Receptor)-Null Mice-Brief Report. Arterioscler Thromb Vasc Biol. 2018; 38:1020-29.

<https://doi.org/10.1161/ATVBAHA.117.310467>

[PMID:29599136](https://www.ncbi.nlm.nih.gov/pubmed/29599136)

54. Wang Y, Song X, Li Z, Liu N, Yan Y, Li T, Sun W, Guan Y, Li M, Yang Y, Yang X, Liu B. MicroRNA-103 Protects Coronary Artery Endothelial Cells against H_2_O_2_-Induced Oxidative Stress via BNIP3-Mediated End-Stage Autophagy and Antipyroptosis Pathways. Oxid Med Cell Longev. 2020; 2020:8351342.

<https://doi.org/10.1155/2020/8351342>

[PMID:32190178](https://www.ncbi.nlm.nih.gov/pubmed/32190178)

55. Chen J, Zhang J, Yang J, Xu L, Hu Q, Xu C, Yang S, Jiang H. Histone demethylase KDM3a, a novel regulator of vascular smooth muscle cells, controls vascular neointimal hyperplasia in diabetic rats. Atherosclerosis. 2017; 257:152-63.

<https://doi.org/10.1016/j.atherosclerosis.2016.12.007>

[PMID:28135625](https://www.ncbi.nlm.nih.gov/pubmed/28135625)

56. Seneviratne AN, Edsfeldt A, Cole JE, Kassiteridi C, Swart M, Park I, Green P, Khoyratty T, Saliba D, Goddard ME, Sansom SN, Goncalves I, Krams R, et al. Interferon Regulatory Factor 5 Controls Necrotic Core Formation in Atherosclerotic Lesions by Impairing Efferocytosis. Circulation. 2017; 136:1140-54.

<https://doi.org/10.1161/CIRCULATIONAHA.117.027844>

[PMID:28698173](https://www.ncbi.nlm.nih.gov/pubmed/28698173)

57. McGrath KC, Li XH, Puranik R, Liong EC, Tan JT, Dy VM, DiBartolo BA, Barter PJ, Rye KA, Heather AK. Role of 3beta-hydroxysteroid-delta 24 reductase in mediating antiinflammatory effects of high-density lipoproteins in endothelial cells. Arterioscler Thromb Vasc Biol. 2009; 29:877-82.

<https://doi.org/10.1161/ATVBAHA.109.184663>

[PMID:19325144](https://www.ncbi.nlm.nih.gov/pubmed/19325144)

58. Baetta R, Lento S, Ghilardi S, Barbati E, Corsini A, Tremoli E, Banfi C. Atorvastatin reduces long pentraxin 3 expression in vascular cells by inhibiting protein geranylgeranylation. Vascul Pharmacol. 2015; 67-69:38-47.

<https://doi.org/10.1016/j.vph.2014.11.008>

[PMID:25849951](https://www.ncbi.nlm.nih.gov/pubmed/25849951)

59. Xu Z, Han Y, Liu J, Jiang F, Hu H, Wang Y, Liu Q, Gong Y, Li X. MiR-135b-5p and MiR-499a-3p Promote Cell Proliferation and Migration in Atherosclerosis by Directly Targeting MEF2C. Sci Rep. 2015; 5:12276.

<https://doi.org/10.1038/srep12276>

[PMID:26184978](https://www.ncbi.nlm.nih.gov/pubmed/26184978)

60. Manichaikul A, Naj AC, Herrington D, Post W, Rich SS, Rodriguez A. Association of SCARB1 variants with subclinical atherosclerosis and incident cardiovascular disease: the multi-ethnic study of atherosclerosis. Arterioscler Thromb Vasc Biol. 2012; 32:1991-99.

<https://doi.org/10.1161/ATVBAHA.112.249714>

[PMID:22628436](https://www.ncbi.nlm.nih.gov/pubmed/22628436)

61. Pi H, Wang Z, Liu M, Deng P, Yu Z, Zhou Z, Gao F. SCD1 activation impedes foam cell formation by inducing lipophagy in oxLDL-treated human vascular smooth muscle cells. J Cell Mol Med. 2019; 23:5259-69.

<https://doi.org/10.1111/jcmm.14401>

[PMID:31119852](https://www.ncbi.nlm.nih.gov/pubmed/31119852)

62. Wang F, Xia W, Liu F, Li J, Wang G, Gu J. Interferon regulator factor 1/retinoic inducible gene I (IRF1/RIG-I) axis mediates 25-hydroxycholesterol-induced interleukin-8 production in atherosclerosis. Cardiovasc Res. 2012; 93:190-99.

<https://doi.org/10.1093/cvr/cvr260>

[PMID:21979142](https://www.ncbi.nlm.nih.gov/pubmed/21979142)

63. Du M, Wang X, Mao X, Yang L, Huang K, Zhang F, Wang Y, Luo X, Wang C, Peng J, Liang M, Huang D, Huang K. Absence of Interferon Regulatory Factor 1 Protects Against Atherosclerosis in Apolipoprotein E-Deficient Mice. Theranostics. 2019; 9:4688-703.

<https://doi.org/10.7150/thno.36862>

[PMID:31367250](https://www.ncbi.nlm.nih.gov/pubmed/31367250)

64. Albasanz-Puig A, Murray J, Preusch M, Coan D, Namekata M, Patel Y, Dong ZM, Rosenfeld ME, Wijelath ES. Oncostatin M is expressed in atherosclerotic lesions: a role for Oncostatin M in the pathogenesis of atherosclerosis. Atherosclerosis. 2011; 216:292-98.

<https://doi.org/10.1016/j.atherosclerosis.2011.02.003>

[PMID:21376322](https://www.ncbi.nlm.nih.gov/pubmed/21376322)

65. Westmuckett AD, Moore KL. Lack of tyrosylprotein sulfotransferase activity in hematopoietic cells drastically attenuates atherosclerosis in Ldlr-/- mice. Arterioscler Thromb Vasc Biol. 2009; 29:1730-36.

<https://doi.org/10.1161/ATVBAHA.109.192963>

[PMID:19679829](https://www.ncbi.nlm.nih.gov/pubmed/19679829)

66. Mendez-Barbero N, Esteban V, Villahoz S, Escolano A, Urso K, Alfranca A, Rodriguez C, Sanchez SA, Osawa T, Andres V, Martinez-Gonzalez J, Minami T, Redondo JM, Campanero MR. A major role for RCAN1 in atherosclerosis progression. EMBO Mol Med. 2013; 5:1901-17.

<https://doi.org/10.1002/emmm.201302842>

[PMID:24127415](https://www.ncbi.nlm.nih.gov/pubmed/24127415)

67. Zhou Y, Miles JR, Tavori H, Lin M, Khoshbouei H, Borchelt DR, Bazick H, Landreth GE, Lee S, Fazio S, Notterpek L. PMP22 Regulates Cholesterol Trafficking and ABCA1-Mediated Cholesterol Efflux. J Neurosci. 2019; 39:5404-18.

<https://doi.org/10.1523/JNEUROSCI.2942-18.2019>

[PMID:31061090](https://www.ncbi.nlm.nih.gov/pubmed/31061090)

68. Mill C, Monk BA, Williams H, Simmonds SJ, Jeremy JY, Johnson JL, George SJ. Wnt5a-induced Wnt1-inducible secreted protein-1 suppresses vascular smooth muscle cell apoptosis induced by oxidative stress. Arterioscler Thromb Vasc Biol. 2014; 34:2449-56.

<https://doi.org/10.1161/ATVBAHA.114.303922>

[PMID:25212236](https://www.ncbi.nlm.nih.gov/pubmed/25212236)

69. Fredman G, Hellmann J, Proto JD, Kuriakose G, Colas RA, Dorweiler B, Connolly ES, Solomon R, Jones DM, Heyer EJ, Spite M, Tabas I. An imbalance between specialized pro-resolving lipid mediators and pro-inflammatory leukotrienes promotes instability of atherosclerotic plaques. Nat Commun. 2016; 7:12859.

<https://doi.org/10.1038/ncomms12859>

[PMID:27659679](https://www.ncbi.nlm.nih.gov/pubmed/27659679)

70. Wang D, Yang Y, Lei Y, Tzvetkov NT, Liu X, Yeung AW, Xu S, Atanasov AG. Targeting Foam Cell Formation in Atherosclerosis: Therapeutic Potential of Natural Products. Pharmacol Rev. 2019; 71:596-670.

<https://doi.org/10.1124/pr.118.017178>

[PMID:31554644](https://www.ncbi.nlm.nih.gov/pubmed/31554644)

71. Zhang L, McCabe T, Condra JH, Ni YG, Peterson LB, Wang W, Strack AM, Wang F, Pandit S, Hammond H, Wood D, Lewis D, Rosa R, et al. An anti-PCSK9 antibody reduces LDL-cholesterol on top of a statin and suppresses hepatocyte SREBP-regulated genes. Int J Biol Sci. 2012; 8:310-27.

<https://doi.org/10.7150/ijbs.3524>

[PMID:22355267](https://www.ncbi.nlm.nih.gov/pubmed/22355267)

72. Saito R, Matsuzaka T, Karasawa T, Sekiya M, Okada N, Igarashi M, Matsumori R, Ishii K, Nakagawa Y, Iwasaki H, Kobayashi K, Yatoh S, Takahashi A, et al. Macrophage Elovl6 deficiency ameliorates foam cell formation and reduces atherosclerosis in low-density lipoprotein receptor-deficient mice. Arterioscler Thromb Vasc Biol. 2011; 31:1973-79.

<https://doi.org/10.1161/ATVBAHA.110.221663>

[PMID:21817094](https://www.ncbi.nlm.nih.gov/pubmed/21817094)

73. Jian D, Wang W, Zhou X, Jia Z, Wang J, Yang M, Zhao W, Jiang Z, Hu X, Zhu J. Interferon-induced protein 35 inhibits endothelial cell proliferation, migration and re-endothelialization of injured arteries by inhibiting the nuclear factor-kappa B pathway. Acta Physiol (Oxf). 2018; 223:e13037.

<https://doi.org/10.1111/apha.13037>

[PMID:29350881](https://www.ncbi.nlm.nih.gov/pubmed/29350881)

74. Irvine KM, Andrews MR, Fernandez-Rojo MA, Schroder K, Burns CJ, Su S, Wilks AF, Parton RG, Hume DA, Sweet MJ. Colony-stimulating factor-1 (CSF-1) delivers a proatherogenic signal to human macrophages. J Leukoc Biol. 2009; 85:278-88.

<https://doi.org/10.1189/jlb.0808497>

[PMID:19004987](https://www.ncbi.nlm.nih.gov/pubmed/19004987)

75. Huang X, Li Y, Li X, Fan D, Xin HB, Fu M. TRIM14 promotes endothelial activation via activating NF-?B signaling pathway. J Mol Cell Biol. 2020; 12:176-89.

<https://doi.org/10.1093/jmcb/mjz040>

[PMID:31070748](https://www.ncbi.nlm.nih.gov/pubmed/31070748)

76. Kotla S, Singh NK, Rao GN. ROS via BTK-p300-STAT1-PPAR? signaling activation mediates cholesterol crystals-induced CD36 expression and foam cell formation. Redox Biol. 2017; 11:350-64.

<https://doi.org/10.1016/j.redox.2016.12.005>

[PMID:28040583](https://www.ncbi.nlm.nih.gov/pubmed/28040583)

77. Pasquin S, Laplante V, Kouadri S, Milasan A, Mayer G, Tormo AJ, Savin V, Sharma M, Martel C, Gauchat JF. Cardiotrophin-like Cytokine Increases Macrophage-Foam Cell Transition. J Immunol. 2018; 201:2462-71.

<https://doi.org/10.4049/jimmunol.1800733>

[PMID:30209193](https://www.ncbi.nlm.nih.gov/pubmed/30209193)

78. Maier A, Wu H, Cordasic N, Oefner P, Dietel B, Thiele C, Weidemann A, Eckardt KU, Warnecke C. Hypoxia-inducible protein 2 Hig2/Hilpda mediates neutral lipid accumulation in macrophages and contributes to atherosclerosis in apolipoprotein E-deficient mice. FASEB J. 2017; 31:4971-84.

<https://doi.org/10.1096/fj.201700235R>

[PMID:28760743](https://www.ncbi.nlm.nih.gov/pubmed/28760743)

79. Apostolakis S, Spandidos D. Chemokines and atherosclerosis: focus on the CX3CL1/CX3CR1 pathway. Acta Pharmacol Sin. 2013; 34:1251-56.

<https://doi.org/10.1038/aps.2013.92>

[PMID:23974513](https://www.ncbi.nlm.nih.gov/pubmed/23974513)

80. Brauner S, Jiang X, Thorlacius GE, Lundberg AM, Ostberg T, Yan ZQ, Kuchroo VK, Hansson GK, Wahren-Herlenius M. Augmented Th17 differentiation in Trim21 deficiency promotes a stable phenotype of atherosclerotic plaques with high collagen content. Cardiovasc Res. 2018; 114:158-67.

<https://doi.org/10.1093/cvr/cvx181>

[PMID:29016728](https://www.ncbi.nlm.nih.gov/pubmed/29016728)

81. Nazarenko MS, Markov AV, Sleptsov AA, Koroleva IA, Sharysh DV, Zarubin AA, Valiahmetov NR, Goncharova IA, Muslimova EF, Kuznecov MS, Kozlov BN, Afanasiev SA, Puzyrev VP. [Comparative analysis of gene expression in vascular cells of patients with advanced atherosclerosis]. Biomed Khim. 2018; 64:416-22.

<https://doi.org/10.18097/PBMC20186405416>

[PMID:30378557](https://www.ncbi.nlm.nih.gov/pubmed/30378557)

82. Strawbridge RJ, Deleskog A, McLeod O, Folkersen L, Kavousi M, Gertow K, Baldassarre D, Veglia F, Leander K, Gigante B, Kauhanen J, Rauramaa R, Smit AJ, et al. A serum 25-hydroxyvitamin D concentration-associated genetic variant in DHCR7 interacts with type 2 diabetes status to influence subclinical atherosclerosis (measured by carotid intima-media thickness). Diabetologia. 2014; 57:1159-72.

<https://doi.org/10.1007/s00125-014-3215-y>

[PMID:24663808](https://www.ncbi.nlm.nih.gov/pubmed/24663808)

83. O Krogmann A, Lusebrink E, Lahrmann C, Flender A, Nickenig G, Zimmer S. Toll-Like Receptor 7 Stimulation Promotes the Development of Atherosclerosis in Apolipoprotein E-Deficient Mice. Int Heart J. 2020; 61:364-72.

<https://doi.org/10.1536/ihj.19-365>

[PMID:32132319](https://www.ncbi.nlm.nih.gov/pubmed/32132319)

84. Karadimou G, Folkersen L, Berg M, Perisic L, Discacciati A, Roy J, Hansson GK, Persson J, Paulsson-Berne G. Low TLR7 gene expression in atherosclerotic plaques is associated with major adverse cardio- and cerebrovascular events. Cardiovasc Res. 2017; 113:30-39.

<https://doi.org/10.1093/cvr/cvw231>

[PMID:27864310](https://www.ncbi.nlm.nih.gov/pubmed/27864310)

85. Liu CL, Santos MM, Fernandes C, Liao M, Iamarene K, Zhang JY, Sukhova GK, Shi GP. Toll-like receptor 7 deficiency protects apolipoprotein E-deficient mice from diet-induced atherosclerosis. Sci Rep. 2017; 7:847.

<https://doi.org/10.1038/s41598-017-00977-0>

[PMID:28405010](https://www.ncbi.nlm.nih.gov/pubmed/28405010)

86. Asdonk T, Steinmetz M, Krogmann A, Strocker C, Lahrmann C, Motz I, Paul-Krahe K, Flender A, Schmitz T, Barchet W, Hartmann G, Nickenig G, Zimmer S. MDA-5 activation by cytoplasmic double-stranded RNA impairs endothelial function and aggravates atherosclerosis. J Cell Mol Med. 2016; 20:1696-705.

<https://doi.org/10.1111/jcmm.12864>

[PMID:27130701](https://www.ncbi.nlm.nih.gov/pubmed/27130701)

87. Piaszyk-Borychowska A, Szeles L, Csermely A, Chiang HC, Wesoly J, Lee CK, Nagy L, Bluyssen HA. Signal Integration of IFN-I and IFN-II With TLR4 Involves Sequential Recruitment of STAT1-Complexes and NF?B to Enhance Pro-inflammatory Transcription. Front Immunol. 2019; 10:1253.

<https://doi.org/10.3389/fimmu.2019.01253>

[PMID:31231385](https://www.ncbi.nlm.nih.gov/pubmed/31231385)

88. Rosenson RS, Tangney CC, Levine DM, Parker TS, Gordon BR. Elevated soluble tumor necrosis factor receptor levels in non-obese adults with the atherogenic dyslipoproteinemia. Atherosclerosis. 2004; 177:77-81.

<https://doi.org/10.1016/j.atherosclerosis.2004.05.027>

[PMID:15488868](https://www.ncbi.nlm.nih.gov/pubmed/15488868)

89. Tawakol A, Singh P, Mojena M, Pimentel-Santillana M, Emami H, MacNabb M, Rudd JH, Narula J, Enriquez JA, Traves PG, Fernandez-Velasco M, Bartrons R, Martin-Sanz P, et al. HIF-1? and PFKFB3 Mediate a Tight Relationship Between Proinflammatory Activation and Anerobic Metabolism in Atherosclerotic Macrophages. Arterioscler Thromb Vasc Biol. 2015; 35:1463-71.

<https://doi.org/10.1161/ATVBAHA.115.305551>

[PMID:25882065](https://www.ncbi.nlm.nih.gov/pubmed/25882065)

90. Lyu M, Cui Y, Zhao T, Ning Z, Ren J, Jin X, Fan G, Zhu Y. *Tnfrsf12a*-Mediated Atherosclerosis Signaling and Inflammatory Response as a Common Protection Mechanism of Shuxuening Injection Against Both Myocardial and Cerebral Ischemia-Reperfusion Injuries. Front Pharmacol. 2018; 9:312.

<https://doi.org/10.3389/fphar.2018.00312>

[PMID:29681850](https://www.ncbi.nlm.nih.gov/pubmed/29681850)

91. Song Z, Wei D, Chen Y, Chen L, Bian Y, Shen Y, Chen J, Pan Y. Association of astragaloside IV-inhibited autophagy and mineralization in vascular smooth muscle cells with lncRNA H19 and DUSP5-mediated ERK signaling. Toxicol Appl Pharmacol. 2019; 364:45-54.

<https://doi.org/10.1016/j.taap.2018.12.002>

[PMID:30529164](https://www.ncbi.nlm.nih.gov/pubmed/30529164)

92. Biros E, Moran CS, Maguire J, Holliday E, Levi C, Golledge J. Upregulation of arylsulfatase B in carotid atherosclerosis is associated with symptoms of cerebral embolization. Sci Rep. 2017; 7:4338.

<https://doi.org/10.1038/s41598-017-04497-9>

[PMID:28659610](https://www.ncbi.nlm.nih.gov/pubmed/28659610)

93. Reynolds LM, Lohman K, Pittman GS, Barr RG, Chi GC, Kaufman J, Wan M, Bell DA, Blaha MJ, Rodriguez CJ, Liu Y. Tobacco exposure-related alterations in DNA methylation and gene expression in human monocytes: the Multi-Ethnic Study of Atherosclerosis (MESA). Epigenetics. 2017; 12:1092-100.

<https://doi.org/10.1080/15592294.2017.1403692>

[PMID:29166816](https://www.ncbi.nlm.nih.gov/pubmed/29166816)

94. Bonta PI, van Tiel CM, Vos M, Pols TW, van Thienen JV, Ferreira V, Arkenbout EK, Seppen J, Spek CA, van der Poll T, Pannekoek H, de Vries CJ. Nuclear receptors Nur77, Nurr1, and NOR-1 expressed in atherosclerotic lesion macrophages reduce lipid loading and inflammatory responses. Arterioscler Thromb Vasc Biol. 2006; 26:2288-94.

<https://doi.org/10.1161/01.ATV.0000238346.84458.5d>

[PMID:16873729](https://www.ncbi.nlm.nih.gov/pubmed/16873729)

95. Swaminathan B, Goikuria H, Vega R, Rodriguez-Antiguedad A, Lopez Medina A, Freijo MM, Vandenbroeck K, Alloza I. Autophagic marker MAP1LC3B expression levels are associated with carotid atherosclerosis symptomatology. PLoS One. 2014; 9:e115176.

<https://doi.org/10.1371/journal.pone.0115176>

[PMID:25503069](https://www.ncbi.nlm.nih.gov/pubmed/25503069)

96. Taylor JM, Borthwick F, Bartholomew C, Graham A. Overexpression of steroidogenic acute regulatory protein increases macrophage cholesterol efflux to apolipoprotein AI. Cardiovasc Res. 2010; 86:526-34.

<https://doi.org/10.1093/cvr/cvq015>

[PMID:20083572](https://www.ncbi.nlm.nih.gov/pubmed/20083572)

97. Yu XH, Zhang DW, Zheng XL, Tang CK. Cholesterol transport system: an integrated cholesterol transport model involved in atherosclerosis. Prog Lipid Res. 2019; 73:65-91.

<https://doi.org/10.1016/j.plipres.2018.12.002>

[PMID:30528667](https://www.ncbi.nlm.nih.gov/pubmed/30528667)

98. Xiong YS, Wu AL, Mu D, Yu J, Zeng P, Sun Y, Xiong J. Inhibition of siglec-1 by lentivirus mediated small interfering RNA attenuates atherogenesis in apoE-deficient mice. Clin Immunol. 2017; 174:32-40.

<https://doi.org/10.1016/j.clim.2016.11.005>

[PMID:27871915](https://www.ncbi.nlm.nih.gov/pubmed/27871915)

99. Bai HL, Lu ZF, Zhao JJ, Ma X, Li XH, Xu H, Wu SG, Kang CM, Lu JB, Xu YJ, Xiao L, Wu Q, Ye S, et al. Microarray profiling analysis and validation of novel long noncoding RNAs and mRNAs as potential biomarkers and their functions in atherosclerosis. Physiol Genomics. 2019; 51:644-56.

<https://doi.org/10.1152/physiolgenomics.00077.2019>

[PMID:31682178](https://www.ncbi.nlm.nih.gov/pubmed/31682178)

100. Stellos K, Gatsiou A, Stamatelopoulos K, Perisic Matic L, John D, Lunella FF, Jae N, Rossbach O, Amrhein C, Sigala F, Boon RA, Furtig B, Manavski Y, et al. Adenosine-to-inosine RNA editing controls cathepsin S expression in atherosclerosis by enabling HuR-mediated post-transcriptional regulation. Nat Med. 2016; 22:1140-50.

<https://doi.org/10.1038/nm.4172>

[PMID:27595325](https://www.ncbi.nlm.nih.gov/pubmed/27595325)

101. Roncal C, Buysschaert I, Gerdes N, Georgiadou M, Ovchinnikova O, Fischer C, Stassen JM, Moons L, Collen D, De Bock K, Hansson GK, Carmeliet P. Short-term delivery of anti-PlGF antibody delays progression of atherosclerotic plaques to vulnerable lesions. Cardiovasc Res. 2010; 86:29-36.

<https://doi.org/10.1093/cvr/cvp380>

[PMID:19952000](https://www.ncbi.nlm.nih.gov/pubmed/19952000)

102. Wang G, Watanabe M, Imai Y, Hara K, Manabe I, Maemura K, Horikoshi M, Kohro T, Amiya E, Sugiyama T, Fujita T, Kadowaki T, Yamazaki T, Nagai R. Genetic variations of Mrf-2/ARID5B confer risk of coronary atherosclerosis in the Japanese population. Int Heart J. 2008; 49:313-27.

<https://doi.org/10.1536/ihj.49.313>

[PMID:18612189](https://www.ncbi.nlm.nih.gov/pubmed/18612189)

103. Westerterp M, Fotakis P, Ouimet M, Bochem AE, Zhang H, Molusky MM, Wang W, Abramowicz S, la Bastide-van Gemert S, Wang N, Welch CL, Reilly MP, Stroes ES, et al. Cholesterol Efflux Pathways Suppress Inflammasome Activation, NETosis, and Atherogenesis. Circulation. 2018; 138:898-912.

<https://doi.org/10.1161/CIRCULATIONAHA.117.032636>

[PMID:29588315](https://www.ncbi.nlm.nih.gov/pubmed/29588315)

104. Wu JF, Wang Y, Zhang M, Tang YY, Wang B, He PP, Lv YC, Ouyang XP, Yao F, Tan YL, Tang SL, Tang DP, Cayabyab FS, et al. Growth differentiation factor-15 induces expression of ATP-binding cassette transporter A1 through PI3-K/PKC?/SP1 pathway in THP-1 macrophages. Biochem Biophys Res Commun. 2014; 444:325-31.

<https://doi.org/10.1016/j.bbrc.2014.01.048>

[PMID:24462860](https://www.ncbi.nlm.nih.gov/pubmed/24462860)

105. Hurtado B, Munoz X, Recarte-Pelz P, Garcia N, Luque A, Krupinski J, Sala N, Garcia de Frutos P. Expression of the vitamin K-dependent proteins GAS6 and protein S and the TAM receptor tyrosine kinases in human atherosclerotic carotid plaques. Thromb Haemost. 2011; 105:873-82.

<https://doi.org/10.1160/TH10-10-0630>

[PMID:21384080](https://www.ncbi.nlm.nih.gov/pubmed/21384080)

106. Lundberg AM, Ketelhuth DF, Johansson ME, Gerdes N, Liu S, Yamamoto M, Akira S, Hansson GK. Toll-like receptor 3 and 4 signalling through the TRIF and TRAM adaptors in haematopoietic cells promotes atherosclerosis. Cardiovasc Res. 2013; 99:364-73.

<https://doi.org/10.1093/cvr/cvt033>

[PMID:23417039](https://www.ncbi.nlm.nih.gov/pubmed/23417039)

107. Dubovyk YI, Oleshko TB, Harbuzova VY, Ataman AV. Positive Association between *EDN1* rs5370 (Lys198Asn) Polymorphism and Large Artery Stroke in a Ukrainian Population. Dis Markers. 2018; 2018:1695782.

<https://doi.org/10.1155/2018/1695782>

[PMID:29849817](https://www.ncbi.nlm.nih.gov/pubmed/29849817)

108. Wolff B, Grabe HJ, Volzke H, Ludemann J, Schwahn C, Freyberger HJ, John U, Lange M, Cascorbi I, Felix SB. A functional serotonin transporter (SLC6A4) polymorphism modifies the association of smoking and diabetes with asymptomatic carotid atherosclerosis. Thromb Haemost. 2005; 93:180-82.

<https://doi.org/10.1055/s-0037-1616164>

[PMID:15630511](https://www.ncbi.nlm.nih.gov/pubmed/15630511)

109. Zhao Y, Howatt DA, Gizard F, Nomiyama T, Findeisen HM, Heywood EB, Jones KL, Conneely OM, Daugherty A, Bruemmer D. Deficiency of the NR4A orphan nuclear receptor NOR1 decreases monocyte adhesion and atherosclerosis. Circ Res. 2010; 107:501-11.

<https://doi.org/10.1161/CIRCRESAHA.110.222083>

[PMID:20558821](https://www.ncbi.nlm.nih.gov/pubmed/20558821)

110. Lim JY, Liu C, Hu KQ, Smith DE, Wu D, Lamon-Fava S, Ausman LM, Wang XD. Dietary ?-Cryptoxanthin Inhibits High-Refined Carbohydrate Diet-Induced Fatty Liver via Differential Protective Mechanisms Depending on Carotenoid Cleavage Enzymes in Male Mice. J Nutr. 2019; 149:1553-64.

<https://doi.org/10.1093/jn/nxz106>

[PMID:31212314](https://www.ncbi.nlm.nih.gov/pubmed/31212314)

111. Finney AC, Funk SD, Green JM, Yurdagul A Jr, Rana MA, Pistorius R, Henry M, Yurochko A, Pattillo CB, Traylor JG, Chen J, Woolard MD, Kevil CG, Orr AW. EphA2 Expression Regulates Inflammation and Fibroproliferative Remodeling in Atherosclerosis. Circulation. 2017; 136:566-82.

<https://doi.org/10.1161/CIRCULATIONAHA.116.026644>

[PMID:28487392](https://www.ncbi.nlm.nih.gov/pubmed/28487392)

112. Pott J, Burkhardt R, Beutner F, Horn K, Teren A, Kirsten H, Holdt LM, Schuler G, Teupser D, Loeffler M, Thiery J, Scholz M. Genome-wide meta-analysis identifies novel loci of plaque burden in carotid artery. Atherosclerosis. 2017; 259:32-40.

<https://doi.org/10.1016/j.atherosclerosis.2017.02.018>

[PMID:28282560](https://www.ncbi.nlm.nih.gov/pubmed/28282560)

113. Hanna RN, Shaked I, Hubbeling HG, Punt JA, Wu R, Herrley E, Zaugg C, Pei H, Geissmann F, Ley K, Hedrick CC. NR4A1 (Nur77) deletion polarizes macrophages toward an inflammatory phenotype and increases atherosclerosis. Circ Res. 2012; 110:416-27.

<https://doi.org/10.1161/CIRCRESAHA.111.253377>

[PMID:22194622](https://www.ncbi.nlm.nih.gov/pubmed/22194622)

114. Matsubara M, Hasegawa K. Benidipine, a dihydropyridine-calcium channel blocker, prevents lysophosphatidylcholine-induced injury and reactive oxygen species production in human aortic endothelial cells. Atherosclerosis. 2005; 178:57-66.

<https://doi.org/10.1016/j.atherosclerosis.2004.08.020>

[PMID:15585201](https://www.ncbi.nlm.nih.gov/pubmed/15585201)

115. Wang KY, Tanimoto A, Guo X, Yamada S, Shimajiri S, Murata Y, Ding Y, Tsutsui M, Kato S, Watanabe T, Ohtsu H, Hirano K, Kohno K, Sasaguri Y. Histamine deficiency decreases atherosclerosis and inflammatory response in apolipoprotein E knockout mice independently of serum cholesterol level. Arterioscler Thromb Vasc Biol. 2011; 31:800-07.

<https://doi.org/10.1161/ATVBAHA.110.215228>

[PMID:21273563](https://www.ncbi.nlm.nih.gov/pubmed/21273563)

116. Xu L, Hao H, Hao Y, Wei G, Li G, Ma P, Xu L, Ding N, Ma S, Chen AF, Jiang Y. Aberrant MFN2 transcription facilitates homocysteine-induced VSMCs proliferation via the increased binding of c-Myc to DNMT1 in atherosclerosis. J Cell Mol Med. 2019; 23:4611-26.

<https://doi.org/10.1111/jcmm.14341>

[PMID:31104361](https://www.ncbi.nlm.nih.gov/pubmed/31104361)

117. Cao Y, Zhou X, Liu H, Zhang Y, Yu X, Liu C. The NF-?B pathway: regulation of the instability of atherosclerotic plaques activated by Fg, Fb, and FDPs. Mol Cell Biochem. 2013; 383:29-37.

<https://doi.org/10.1007/s11010-013-1751-2>

[PMID:23839109](https://www.ncbi.nlm.nih.gov/pubmed/23839109)

118. Goossens P, Gijbels MJ, Zernecke A, Eijgelaar W, Vergouwe MN, van der Made I, Vanderlocht J, Beckers L, Buurman WA, Daemen MJ, Kalinke U, Weber C, Lutgens E, de Winther MP. Myeloid type I interferon signaling promotes atherosclerosis by stimulating macrophage recruitment to lesions. Cell Metab. 2010; 12:142-53.

<https://doi.org/10.1016/j.cmet.2010.06.008>

[PMID:20674859](https://www.ncbi.nlm.nih.gov/pubmed/20674859)

119. Chapoval SP, Vadasz Z, Chapoval AI, Toubi E. Semaphorins 4A and 4D in chronic inflammatory diseases. Inflamm Res. 2017; 66:111-117.

<https://doi.org/10.1007/s00011-016-0983-5> PMID:[27554682](https://pubmed.ncbi.nlm.nih.gov/27554682)

120. Ryu H, Lim H, Choi G, Park YJ, Cho M, Na H, Ahn CW, Kim YC, Kim WU, Lee SH, Chung Y. Atherogenic dyslipidemia promotes autoimmune follicular helper T cell responses via IL-27. Nat Immunol. 2018; 19:583-93.

<https://doi.org/10.1038/s41590-018-0102-6>

[PMID:29713015](https://www.ncbi.nlm.nih.gov/pubmed/29713015)

121. Correction to. Correction to: Macrophage-Associated Lipin-1 Enzymatic Activity Contributes to Modified Low-Density Lipoprotein-Induced Proinflammatory Signaling and Atherosclerosis. Arterioscler Thromb Vasc Biol. 2018; 38:e89.

<https://doi.org/10.1161/ATV.0000000000000070>

[PMID:29695536](https://www.ncbi.nlm.nih.gov/pubmed/29695536)

122. Di Bartolo BA, Chan J, Bennett MR, Cartland S, Bao S, Tuch BE, Kavurma MM. TNF-related apoptosis-inducing ligand (TRAIL) protects against diabetes and atherosclerosis in Apoe ?/? mice. Diabetologia. 2011; 54:3157-67.

<https://doi.org/10.1007/s00125-011-2308-0>

[PMID:21965021](https://www.ncbi.nlm.nih.gov/pubmed/21965021)

123. Pan X, Pang M, Ma A, Wang K, Zhang Z, Zhong Q, Yang S. Association of TRAIL and Its Receptors with Large-Artery Atherosclerotic Stroke. PLoS One. 2015; 10:e0136414.

<https://doi.org/10.1371/journal.pone.0136414>

[PMID:26334877](https://www.ncbi.nlm.nih.gov/pubmed/26334877)

124. Qiu G, Hill JS. Endothelial lipase promotes apolipoprotein AI-mediated cholesterol efflux in THP-1 macrophages. Arterioscler Thromb Vasc Biol. 2009; 29:84-91.

<https://doi.org/10.1161/ATVBAHA.108.176487>

[PMID:18988890](https://www.ncbi.nlm.nih.gov/pubmed/18988890)

125. Gonzalez-Cotto M, Guo L, Karwan M, Sen SK, Barb J, Collado CJ, Elloumi F, Palmieri EM, Boelte K, Kolodgie FD, Finn AV, Biesecker LG, McVicar DW. *TREML4* Promotes Inflammatory Programs in Human and Murine Macrophages and Alters Atherosclerosis Lesion Composition in the Apolipoprotein E Deficient Mouse. Front Immunol. 2020; 11:397.

<https://doi.org/10.3389/fimmu.2020.00397>

[PMID:32292401](https://www.ncbi.nlm.nih.gov/pubmed/32292401)

126. Roy A, Banerjee S, Saqib U, Baig MS. NOS1-derived nitric oxide facilitates macrophage uptake of low-density lipoprotein. J Cell Biochem. 2019; 120:11593-603.

<https://doi.org/10.1002/jcb.28439>

[PMID:30805961](https://www.ncbi.nlm.nih.gov/pubmed/30805961)

127. McCarthy C, Lee E, Bridges JP, Sallese A, Suzuki T, Woods JC, Bartholmai BJ, Wang T, Chalk C, Carey BC, Arumugam P, Shima K, Tarling EJ, Trapnell BC. Statin as a novel pharmacotherapy of pulmonary alveolar proteinosis. Nat Commun. 2018; 9:3127.

<https://doi.org/10.1038/s41467-018-05491-z>

[PMID:30087322](https://www.ncbi.nlm.nih.gov/pubmed/30087322)

128. Yuan H, Zelkha S, Burkatovskaya M, Gupte R, Leeman SE, Amar S. Pivotal role of NOD2 in inflammatory processes affecting atherosclerosis and periodontal bone loss. Proc Natl Acad Sci USA. 2013; 110:E5059-68.

<https://doi.org/10.1073/pnas.1320862110>

[PMID:24324141](https://www.ncbi.nlm.nih.gov/pubmed/24324141)

129. Klein R, Knudtson MD, Klein BE, Wong TY, Cotch MF, Liu K, Cheng CY, Burke GL, Saad MF, Jacobs DR Jr, Sharrett AR. Inflammation, complement factor h, and age-related macular degeneration: the Multi-ethnic Study of Atherosclerosis. Ophthalmology. 2008; 115:1742-49.

<https://doi.org/10.1016/j.ophtha.2008.03.021>

[PMID:18538409](https://www.ncbi.nlm.nih.gov/pubmed/18538409)

130. Tousoulis D, Kioufis S, Siasos G, Oikonomou E, Zaromitidou M, Maniatis K, Kokkou E, Mazaris S, Zakynthinos G, Konsola T, Stefanadis C. The impact of AMPD1 gene polymorphism on vascular function and inflammation in patients with coronary artery disease. Int J Cardiol. 2014; 172:e516-18.

<https://doi.org/10.1016/j.ijcard.2014.01.078>

[PMID:24508110](https://www.ncbi.nlm.nih.gov/pubmed/24508110)

131. Demetz E, Tymoszuk P, Hilbe R, Volani C, Haschka D, Heim C, Auer K, Lener D, Zeiger LB, Pfeifhofer-Obermair C, Boehm A, Obermair GJ, Ablinger C, et al. The haemochromatosis gene Hfe and Kupffer cells control LDL cholesterol homeostasis and impact on atherosclerosis development. Eur Heart J. 2020; 41:3949-59.

<https://doi.org/10.1093/eurheartj/ehaa140>

[PMID:32227235](https://www.ncbi.nlm.nih.gov/pubmed/32227235)

132. Byun S, Jung H, Chen J, Kim YC, Kim DH, Kong B, Guo G, Kemper B, Kemper JK. Phosphorylation of hepatic farnesoid X receptor by FGF19 signaling-activated Src maintains cholesterol levels and protects from atherosclerosis. J Biol Chem. 2019; 294:8732-44.

<https://doi.org/10.1074/jbc.RA119.008360>

[PMID:30996006](https://www.ncbi.nlm.nih.gov/pubmed/30996006)

133. Rolfe BE, Stamatiou S, World CJ, Brown L, Thomas AC, Bingley JA, Worth NF, Campbell JH. Leukaemia inhibitory factor retards the progression of atherosclerosis. Cardiovasc Res. 2003; 58:222-30.

<https://doi.org/10.1016/S0008-6363(02)00832-5>

[PMID:12667965](https://www.ncbi.nlm.nih.gov/pubmed/12667965)

134. Perdomo G, Henry Dong H. Apolipoprotein D in lipid metabolism and its functional implication in atherosclerosis and aging. Aging (Albany NY). 2009; 1:17-27.

<https://doi.org/10.18632/aging.100004>

[PMID:19946382](https://www.ncbi.nlm.nih.gov/pubmed/19946382)

135. Zhang F, Sodroski C, Cha H, Li Q, Liang TJ. Infection of Hepatocytes With HCV Increases Cell Surface Levels of Heparan Sulfate Proteoglycans, Uptake of Cholesterol and Lipoprotein, and Virus Entry by Up-regulating SMAD6 and SMAD7. Gastroenterology. 2017; 152:257-270.e7.

<https://doi.org/10.1053/j.gastro.2016.09.033>

[PMID:27693511](https://www.ncbi.nlm.nih.gov/pubmed/27693511)

136. Berisha SZ, Hsu J, Robinet P, Smith JD. Transcriptome analysis of genes regulated by cholesterol loading in two strains of mouse macrophages associates lysosome pathway and ER stress response with atherosclerosis susceptibility. PLoS One. 2013; 8:e65003.

<https://doi.org/10.1371/journal.pone.0065003>

[PMID:23705026](https://www.ncbi.nlm.nih.gov/pubmed/23705026)

137. Yang Y, Yang L, Liang X, Zhu G. MicroRNA-155 Promotes Atherosclerosis Inflammation via Targeting SOCS1. Cell Physiol Biochem. 2015;36:1371-1381.

<https://doi.org/10.1159/000430303> PMID:[26159489](https://pubmed.ncbi.nlm.nih.gov/26159489)

138. Delerive P, Monte D, Dubois G, Trottein F, Fruchart-Najib J, Mariani J, Fruchart JC, Staels B. The orphan nuclear receptor ROR alpha is a negative regulator of the inflammatory response. EMBO Rep. 2001; 2:42-48.

<https://doi.org/10.1093/embo-reports/kve007>

[PMID:11252722](https://www.ncbi.nlm.nih.gov/pubmed/11252722)

139. Lichtenstein L, Serhan N, Espinosa-Delgado S, Fabre A, Annema W, Tietge UJ, Robaye B, Boeynaems JM, Laffargue M, Perret B, Martinez LO. Increased atherosclerosis in P2Y13/apolipoprotein E double-knockout mice: contribution of P2Y13 to reverse cholesterol transport. Cardiovasc Res. 2015; 106:314-23.

<https://doi.org/10.1093/cvr/cvv109>

[PMID:25770145](https://www.ncbi.nlm.nih.gov/pubmed/25770145)

140. Doddapattar P, Dhanesha N, Chorawala MR, Tinsman C, Jain M, Nayak MK, Staber JM, Chauhan AK. Endothelial Cell-Derived Von Willebrand Factor, But Not Platelet-Derived, Promotes Atherosclerosis in Apolipoprotein E-Deficient Mice. Arterioscler Thromb Vasc Biol. 2018; 38:520-28.

<https://doi.org/10.1161/ATVBAHA.117.309918>

[PMID:29348121](https://www.ncbi.nlm.nih.gov/pubmed/29348121)

141. Rebe C, Raveneau M, Chevriaux A, Lakomy D, Sberna AL, Costa A, Bessede G, Athias A, Steinmetz E, Lobaccaro JM, Alves G, Menicacci A, Vachenc S, et al. Induction of transglutaminase 2 by a liver X receptor/retinoic acid receptor alpha pathway increases the clearance of apoptotic cells by human macrophages. Circ Res. 2009; 105:393-401.

<https://doi.org/10.1161/CIRCRESAHA.109.201855>

[PMID:19628791](https://www.ncbi.nlm.nih.gov/pubmed/19628791)

142. Liu Y, Cheng J, Guo X, Mo J, Gao B, Zhou H, Wu Y, Li Z. The roles of PAI-1 gene polymorphisms in atherosclerotic diseases: A systematic review and meta-analysis involving 149,908 subjects. Gene. 2018; 673:167-73.

<https://doi.org/10.1016/j.gene.2018.06.040>

[PMID:29908999](https://www.ncbi.nlm.nih.gov/pubmed/29908999)

143. Gertow K, Sennblad B, Strawbridge RJ, Ohrvik J, Zabaneh D, Shah S, Veglia F, Fava C, Kavousi M, McLachlan S, Kivimaki M, Bolton JL, Folkersen L, et al. Identification of the BCAR1-CFDP1-TMEM170A locus as a determinant of carotid intima-media thickness and coronary artery disease risk. Circ Cardiovasc Genet. 2012; 5:656-65.

<https://doi.org/10.1161/CIRCGENETICS.112.963660>

[PMID:23152477](https://www.ncbi.nlm.nih.gov/pubmed/23152477)

144. Lutgens E, Lutgens SP, Faber BC, Heeneman S, Gijbels MM, de Winther MP, Frederik P, van der Made I, Daugherty A, Sijbers AM, Fisher A, Long CJ, Saftig P, et al. Disruption of the cathepsin K gene reduces atherosclerosis progression and induces plaque fibrosis but accelerates macrophage foam cell formation. Circulation. 2006; 113:98-107.

<https://doi.org/10.1161/CIRCULATIONAHA.105.561449>

[PMID:16365196](https://www.ncbi.nlm.nih.gov/pubmed/16365196)

145. Arvind P, Nair J, Jambunathan S, Kakkar VV, Shanker J. CELSR2-PSRC1-SORT1 gene expression and association with coronary artery disease and plasma lipid levels in an Asian Indian cohort. J Cardiol. 2014; 64:339-46.

<https://doi.org/10.1016/j.jjcc.2014.02.012>

[PMID:24674750](https://www.ncbi.nlm.nih.gov/pubmed/24674750)

146. Bodary PF, Shen Y, Vargas FB, Bi X, Ostenso KA, Gu S, Shayman JA, Eitzman DT. Alpha-galactosidase A deficiency accelerates atherosclerosis in mice with apolipoprotein E deficiency. Circulation. 2005; 111:629-32.

<https://doi.org/10.1161/01.CIR.0000154550.15963.80>

[PMID:15668341](https://www.ncbi.nlm.nih.gov/pubmed/15668341)

147. Sandberg WJ, Yndestad A, Oie E, Smith C, Ueland T, Ovchinnikova O, Robertson AK, Muller F, Semb AG, Scholz H, Andreassen AK, Gullestad L, Damas JK, et al. Enhanced T-cell expression of RANK ligand in acute coronary syndrome: possible role in plaque destabilization. Arterioscler Thromb Vasc Biol. 2006; 26:857-63.

<https://doi.org/10.1161/01.ATV.0000204334.48195.6a>

[PMID:16424351](https://www.ncbi.nlm.nih.gov/pubmed/16424351)

148. Agardh HE, Folkersen L, Ekstrand J, Marcus D, Swedenborg J, Hedin U, Gabrielsen A, Paulsson-Berne G. Expression of fatty acid-binding protein 4/aP2 is correlated with plaque instability in carotid atherosclerosis. J Intern Med. 2011; 269:200-10.

<https://doi.org/10.1111/j.1365-2796.2010.02304.x>

[PMID:21073559](https://www.ncbi.nlm.nih.gov/pubmed/21073559)

149. Trigueros-Motos L, van Capelleveen JC, Torta F, Castano D, Zhang LH, Chai EC, Kang M, Dimova LG, Schimmel AW, Tietjen I, Radomski C, Tan LJ, Thiam CH, et al. ABCA8 Regulates Cholesterol Efflux and High-Density Lipoprotein Cholesterol Levels. Arterioscler Thromb Vasc Biol. 2017; 37:2147-55.

<https://doi.org/10.1161/ATVBAHA.117.309574>

[PMID:28882873](https://www.ncbi.nlm.nih.gov/pubmed/28882873)

150. Aldi S, Matic LP, Hamm G, van Keulen D, Tempel D, Holmstrom K, Szwajda A, Nielsen BS, Emilsson V, Ait-Belkacem R, Lengquist M, Paulsson-Berne G, Eriksson P, et al. Integrated Human Evaluation of the Lysophosphatidic Acid Pathway as a Novel Therapeutic Target in Atherosclerosis. Mol Ther Methods Clin Dev. 2018; 10:17-28.

<https://doi.org/10.1016/j.omtm.2018.05.003>

[PMID:30003117](https://www.ncbi.nlm.nih.gov/pubmed/30003117)

151. Srikakulapu P, Hu D, Yin C, Mohanta SK, Bontha SV, Peng L, Beer M, Weber C, McNamara CA, Grassia G, Maffia P, Manz RA, Habenicht AJ. Artery Tertiary Lymphoid Organs Control Multilayered Territorialized Atherosclerosis B-Cell Responses in Aged ApoE-/- Mice. Arterioscler Thromb Vasc Biol. 2016; 36:1174-85.

<https://doi.org/10.1161/ATVBAHA.115.306983>

[PMID:27102965](https://www.ncbi.nlm.nih.gov/pubmed/27102965)

152. Reimers GJ, Jackson CL, Rickards J, Chan PY, Cohn JS, Rye KA, Barter PJ, Rodgers KJ. Inhibition of rupture of established atherosclerotic plaques by treatment with apolipoprotein A-I. Cardiovasc Res. 2011; 91:37-44.

<https://doi.org/10.1093/cvr/cvr057>

[PMID:21354994](https://www.ncbi.nlm.nih.gov/pubmed/21354994)

153. Gertow K, Nobili E, Folkersen L, Newman JW, Pedersen TL, Ekstrand J, Swedenborg J, Kuhn H, Wheelock CE, Hansson GK, Hedin U, Haeggstrom JZ, Gabrielsen A. 12- and 15-lipoxygenases in human carotid atherosclerotic lesions: associations with cerebrovascular symptoms. Atherosclerosis. 2011; 215:411-16.

<https://doi.org/10.1016/j.atherosclerosis.2011.01.015>

[PMID:21316676](https://www.ncbi.nlm.nih.gov/pubmed/21316676)

154. Suffee N, Hlawaty H, Meddahi-Pelle A, Maillard L, Louedec L, Haddad O, Martin L, Laguillier C, Richard B, Oudar O, Letourneur D, Charnaux N, Sutton A. RANTES/CCL5-induced pro-angiogenic effects depend on CCR1, CCR5 and glycosaminoglycans. Angiogenesis. 2012; 15:727-44.

<https://doi.org/10.1007/s10456-012-9285-x>

[PMID:22752444](https://www.ncbi.nlm.nih.gov/pubmed/22752444)

155. Chistiakov DA, Orekhov AN, Bobryshev YV. The role of miR-126 in embryonic angiogenesis, adult vascular homeostasis, and vascular repair and its alterations in atherosclerotic disease. J Mol Cell Cardiol. 2016; 97:47-55.

<https://doi.org/10.1016/j.yjmcc.2016.05.007>

[PMID:27180261](https://www.ncbi.nlm.nih.gov/pubmed/27180261)

156. Sandberg WJ, Halvorsen B, Yndestad A, Smith C, Otterdal K, Brosstad FR, Froland SS, Olofsson PS, Damas JK, Gullestad L, Hansson GK, Oie E, Aukrust P. Inflammatory interaction between LIGHT and proteinase-activated receptor-2 in endothelial cells: potential role in atherogenesis. Circ Res. 2009; 104:60-68.

<https://doi.org/10.1161/CIRCRESAHA.108.188078>

[PMID:19023130](https://www.ncbi.nlm.nih.gov/pubmed/19023130)

157. Ramazi S, Heydari-Zarnagh H, Goudarzian M, Khalaj-Kondori M, Bonyadi M. Thromboxane A synthase 1 gene expression and promotor haplotypes are associated with risk of large artery-atherosclerosis stroke in Iranian population. J Cell Biochem. 2019; 120:15222-32.

<https://doi.org/10.1002/jcb.28787>

[PMID:31026093](https://www.ncbi.nlm.nih.gov/pubmed/31026093)

158. He H, Li R, Choi S, Zhou L, Pavel A, Estrada YD, Krueger JG, Guttman-Yassky E. Increased cardiovascular and atherosclerosis markers in blood of older patients with atopic dermatitis. Ann Allergy Asthma Immunol. 2020; 124:70-78.

<https://doi.org/10.1016/j.anai.2019.10.013>

[PMID:31622668](https://www.ncbi.nlm.nih.gov/pubmed/31622668)

159. Lanuti M, Talamonti E, Maccarrone M, Chiurchiu V. Activation of GPR55 Receptors Exacerbates oxLDL-Induced Lipid Accumulation and Inflammatory Responses, while Reducing Cholesterol Efflux from Human Macrophages. PLoS One. 2015; 10:e0126839.

<https://doi.org/10.1371/journal.pone.0126839>

[PMID:25970609](https://www.ncbi.nlm.nih.gov/pubmed/25970609)

160. Wolfrum C, Poy MN, Stoffel M. Apolipoprotein M is required for prebeta-HDL formation and cholesterol efflux to HDL and protects against atherosclerosis. Nat Med. 2005; 11:418-22.

<https://doi.org/10.1038/nm1211>

[PMID:15793583](https://www.ncbi.nlm.nih.gov/pubmed/15793583)

161. Wang G, Qiu J, Hu J, Tang C, Yin T. Id1: a novel therapeutic target for patients with atherosclerotic plaque rupture. Med Hypotheses. 2011; 76:627-28.

<https://doi.org/10.1016/j.mehy.2011.01.014>

[PMID:21288647](https://www.ncbi.nlm.nih.gov/pubmed/21288647)

162. Zhang Y, Zheng L, Xu BM, Tang WH, Ye ZD, Huang C, Ma X, Zhao JJ, Guo FX, Kang CM, Lu JB, Xiu JC, Li P, et al. LncRNA-RP11-714G18.1 suppresses vascular cell migration via directly targeting LRP2BP. Immunol Cell Biol. 2018; 96:175-89.

<https://doi.org/10.1111/imcb.1028>

[PMID:29363163](https://www.ncbi.nlm.nih.gov/pubmed/29363163)

163. Kim YC, Byun S, Seok S, Guo G, Xu HE, Kemper B, Kemper JK. Small Heterodimer Partner and Fibroblast Growth Factor 19 Inhibit Expression of NPC1L1 in Mouse Intestine and Cholesterol Absorption. Gastroenterology. 2019; 156:1052-65.

<https://doi.org/10.1053/j.gastro.2018.11.061>

[PMID:30521806](https://www.ncbi.nlm.nih.gov/pubmed/30521806)

164. Mitrofan CG, Appleby SL, Nash GB, Mallat Z, Chilvers ER, Upton PD, Morrell NW. Bone morphogenetic protein 9 (BMP9) and BMP10 enhance tumor necrosis factor-?-induced monocyte recruitment to the vascular endothelium mainly via activin receptor-like kinase 2. J Biol Chem. 2017; 292:13714-26.

<https://doi.org/10.1074/jbc.M117.778506>

[PMID:28646109](https://www.ncbi.nlm.nih.gov/pubmed/28646109)

165. Holloway JW, Laxton RC, Rose-Zerilli MJ, Holloway JA, Andrews AL, Riaz Z, Wilson SJ, Simpson IA, Ye S. ADAM33 expression in atherosclerotic lesions and relationship of ADAM33 gene variation with atherosclerosis. Atherosclerosis. 2010; 211:224-30.

<https://doi.org/10.1016/j.atherosclerosis.2010.02.023>

[PMID:20227692](https://www.ncbi.nlm.nih.gov/pubmed/20227692)

166. de Boer OJ, van der Meer JJ, Teeling P, van der Loos CM, Idu MM, van Maldegem F, Aten J, van der Wal AC. Differential expression of interleukin-17 family cytokines in intact and complicated human atherosclerotic plaques. J Pathol. 2010; 220:499-508.

<https://doi.org/10.1002/path.2667>

[PMID:20020510](https://www.ncbi.nlm.nih.gov/pubmed/20020510)

167. Esteghamat F, Broughton JS, Smith E, Cardone R, Tyagi T, Guerra M, Szabo A, Ugwu N, Mani MV, Azari B, Kayingo G, Chung S, Fathzadeh M, et al. CELA2A mutations predispose to early-onset atherosclerosis and metabolic syndrome and affect plasma insulin and platelet activation. Nat Genet. 2019; 51:1233-43.

<https://doi.org/10.1038/s41588-019-0470-3>

[PMID:31358993](https://www.ncbi.nlm.nih.gov/pubmed/31358993)

168. Wang HH, Afdhal NH, Gendler SJ, Wang DQ. Lack of the intestinal Muc1 mucin impairs cholesterol uptake and absorption but not fatty acid uptake in Muc1-/- mice. Am J Physiol Gastrointest Liver Physiol. 2004; 287:G547-54.

<https://doi.org/10.1152/ajpgi.00097.2004>

[PMID:15075252](https://www.ncbi.nlm.nih.gov/pubmed/15075252)

169. Pourcet B, Feig JE, Vengrenyuk Y, Hobbs AJ, Kepka-Lenhart D, Garabedian MJ, Morris SM Jr, Fisher EA, Pineda-Torra I. LXR? regulates macrophage arginase 1 through PU.1 and interferon regulatory factor 8. Circ Res. 2011; 109:492-501.

<https://doi.org/10.1161/CIRCRESAHA.111.241810>

[PMID:21757649](https://www.ncbi.nlm.nih.gov/pubmed/21757649)

170. Turkanoglu Ozcelik A, Can Demirdogen B, Demirkaya S, Adali O. Association of cytochrome P4502E1 and NAD(P)H:quinone oxidoreductase 1 genetic polymorphisms with susceptibility to large artery atherosclerotic ischemic stroke: a case-control study in the Turkish population. Neurol Sci. 2017; 38:1077-1085.

<https://doi.org/10.1007/s10072-017-2930-9> PMID:[28357584](https://pubmed.ncbi.nlm.nih.gov/28357584)

171. Meletta R, Slavik R, Mu L, Rancic Z, Borel N, Schibli R, Ametamey SM, Kramer SD, Muller Herde A. Cannabinoid receptor type 2 (CB2) as one of the candidate genes in human carotid plaque imaging: evaluation of the novel radiotracer [^11^C]RS-016 targeting CB2 in atherosclerosis. Nucl Med Biol. 2017; 47:31-43.

<https://doi.org/10.1016/j.nucmedbio.2017.01.001>

[PMID:28104528](https://www.ncbi.nlm.nih.gov/pubmed/28104528)

172. Douna H, Amersfoort J, Schaftenaar FH, Kroner MJ, Kiss MG, Slutter B, Depuydt MA, Bernabe Kleijn MN, Wezel A, Smeets HJ, Yagita H, Binder CJ, Bot I, et al. B- and T-lymphocyte attenuator stimulation protects against atherosclerosis by regulating follicular B cells. Cardiovasc Res. 2020; 116:295-305.

<https://doi.org/10.1093/cvr/cvz129>

[PMID:31150053](https://www.ncbi.nlm.nih.gov/pubmed/31150053)

173. Gomez M, Sanz-Gonzalez SM, Abu Nabah YN, Lamana A, Sanchez-Madrid F, Andres V. Atherosclerosis development in apolipoprotein E-null mice deficient for CD69. Cardiovasc Res. 2009; 81:197-205.

<https://doi.org/10.1093/cvr/cvn227>

[PMID:18703531](https://www.ncbi.nlm.nih.gov/pubmed/18703531)

174. Decharatchakul N, Settasatian C, Settasatian N, Komanasin N, Kukongviriyapan U, Intharaphet P, Senthong V. Association of genetic polymorphisms in *SOD2*, *SOD3*, *GPX3*, and *GSTT1* with hypertriglyceridemia and low HDL-C level in subjects with high risk of coronary artery disease. PeerJ. 2019; 7:e7407.

<https://doi.org/10.7717/peerj.7407>

[PMID:31396447](https://www.ncbi.nlm.nih.gov/pubmed/31396447)

175. Upadhye A, Srikakulapu P, Gonen A, Hendrikx S, Perry HM, Nguyen A, McSkimming C, Marshall MA, Garmey JC, Taylor AM, Bender TP, Tsimikas S, Holodick NE, et al. Diversification and CXCR4-Dependent Establishment of the Bone Marrow B-1a Cell Pool Governs Atheroprotective IgM Production Linked to Human Coronary Atherosclerosis. Circ Res. 2019; 125:e55-70.

<https://doi.org/10.1161/CIRCRESAHA.119.315786>

[PMID:31549940](https://www.ncbi.nlm.nih.gov/pubmed/31549940)

176. Wolfrum S, Rodriguez JM, Tan M, Chen KY, Teupser D, Breslow JL. The mouse atherosclerosis locus at chromosome 10 (Ath11) acts early in lesion formation with subcongenic strains delineating 2 narrowed regions. Arterioscler Thromb Vasc Biol. 2010; 30:1583-90.

<https://doi.org/10.1161/ATVBAHA.110.205757>

[PMID:20466976](https://www.ncbi.nlm.nih.gov/pubmed/20466976)

177. Shentu TP, He M, Sun X, Zhang J, Zhang F, Gongol B, Marin TL, Zhang J, Wen L, Wang Y, Geary GG, Zhu Y, Johnson DA, Shyy JY. AMP-Activated Protein Kinase and Sirtuin 1 Coregulation of Cortactin Contributes to Endothelial Function. Arterioscler Thromb Vasc Biol. 2016; 36:2358-68.

<https://doi.org/10.1161/ATVBAHA.116.307871>

[PMID:27758765](https://www.ncbi.nlm.nih.gov/pubmed/27758765)

178. Luyckx E, Everaert BR, Van der Veken B, Van Leuven W, Timmermans JP, Vrints CJ, De Meyer GR, Martinet W, Dewilde S. Cytoprotective effects of transgenic neuroglobin overexpression in an acute and chronic mouse model of ischemic heart disease. Heart Vessels. 2018; 33:80-88.

<https://doi.org/10.1007/s00380-017-1065-5>

[PMID:29098407](https://www.ncbi.nlm.nih.gov/pubmed/29098407)

179. Helgadottir A, Thorleifsson G, Gretarsdottir S, Stefansson OA, Tragante V, Thorolfsdottir RB, Jonsdottir I, Bjornsson T, Steinthorsdottir V, Verweij N, Nielsen JB, Zhou W, Folkersen L, et al. Genome-wide analysis yields new loci associating with aortic valve stenosis. Nat Commun. 2018; 9:987.

<https://doi.org/10.1038/s41467-018-03252-6>

[PMID:29511194](https://www.ncbi.nlm.nih.gov/pubmed/29511194)

180. Chen YC, Rivera J, Fitzgerald M, Hausding C, Ying YL, Wang X, Todorova K, Hayrabedyan S, Barnea ER, Peter K. PreImplantation factor prevents atherosclerosis via its immunomodulatory effects without affecting serum lipids. Thromb Haemost. 2016; 115:1010-24.

<https://doi.org/10.1160/TH15-08-0640>

[PMID:26842698](https://www.ncbi.nlm.nih.gov/pubmed/26842698)

181. Hughes SE. Localisation and differential expression of the fibroblast growth factor receptor (FGFR) multigene family in normal and atherosclerotic human arteries. Cardiovasc Res. 1996; 32:557-69.

<https://doi.org/10.1016/S0008-6363(96)00102-2>

[PMID:8881516](https://www.ncbi.nlm.nih.gov/pubmed/8881516)

182. Xia Z, Gu M, Jia X, Wang X, Wu C, Guo J, Zhang L, Du Y, Wang J. Integrated DNA methylation and gene expression analysis identifies SLAMF7 as a key regulator of atherosclerosis. Aging (Albany NY). 2018; 10:1324-37.

<https://doi.org/10.18632/aging.101470>

[PMID:29905534](https://www.ncbi.nlm.nih.gov/pubmed/29905534)

183. De Iuliis V, Ursi S, Pennelli A, Caruso M, Capodifoglio S, Marino A, Flati V, Vitullo G, Toniato E, Robuffo I, Martinotti S. A Method to Study the C924T Polymorphism of the Thromboxane A2 Receptor Gene. J Vis Exp. 2019.

<https://doi.org/10.3791/57289>

[PMID:30985753](https://www.ncbi.nlm.nih.gov/pubmed/30985753)

184. Rinne P, Rami M, Nuutinen S, Santovito D, van der Vorst EP, Guillamat-Prats R, Lyytikainen LP, Raitoharju E, Oksala N, Ring L, Cai M, Hruby VJ, Lehtimaki T, et al. Melanocortin 1 Receptor Signaling Regulates Cholesterol Transport in Macrophages. Circulation. 2017; 136:83-97.

<https://doi.org/10.1161/CIRCULATIONAHA.116.025889>

[PMID:28450348](https://www.ncbi.nlm.nih.gov/pubmed/28450348)

185. Nolan DK, Sutton B, Haynes C, Johnson J, Sebek J, Dowdy E, Crosslin D, Crossman D, Sketch MH Jr, Granger CB, Seo D, Goldschmidt-Clermont P, Kraus WE, et al. Fine mapping of a linkage peak with integration of lipid traits identifies novel coronary artery disease genes on chromosome 5. BMC Genet. 2012; 13:12.

<https://doi.org/10.1186/1471-2156-13-12>

[PMID:22369142](https://www.ncbi.nlm.nih.gov/pubmed/22369142)

186. Kang YJ, Kim WJ, Bae HU, Kim DI, Park YB, Park JE, Kwon BS, Lee WH. Involvement of TL1A and DR3 in induction of pro-inflammatory cytokines and matrix metalloproteinase-9 in atherogenesis. Cytokine. 2005; 29:229-35.

<https://doi.org/10.1016/j.cyto.2004.12.001>

[PMID:15760679](https://www.ncbi.nlm.nih.gov/pubmed/15760679)

187. Shao B, Tang C, Sinha A, Mayer PS, Davenport GD, Brot N, Oda MN, Zhao XQ, Heinecke JW. Humans with atherosclerosis have impaired ABCA1 cholesterol efflux and enhanced high-density lipoprotein oxidation by myeloperoxidase. Circ Res. 2014; 114:1733-42.

<https://doi.org/10.1161/CIRCRESAHA.114.303454>

[PMID:24647144](https://www.ncbi.nlm.nih.gov/pubmed/24647144)

188. Wan Q, Liu Z, Yang M, Wu J. Acceleratory effects of ambient fine particulate matter on the development and progression of atherosclerosis in apolipoprotein E knockout mice by down-regulating CD4^+^CD25^+^Foxp3^+^ regulatory T cells. Toxicol Lett. 2019; 316:27-34.

<https://doi.org/10.1016/j.toxlet.2019.09.005>

[PMID:31513887](https://www.ncbi.nlm.nih.gov/pubmed/31513887)

189. Kheirollah A, Nagayasu Y, Ueda H, Yokoyama S, Michikawa M, Ito J. Involvement of cdc42/Rho kinase in apoA-I-mediated cholesterol efflux through interaction between cytosolic lipid-protein particles and microtubules in rat astrocytes. J Neurosci Res. 2014; 92:455-63.

<https://doi.org/10.1002/jnr.23324>

[PMID:24446142](https://www.ncbi.nlm.nih.gov/pubmed/24446142)

190. Liang C, Wang X, Hu J, Lian X, Zhu T, Zhang H, Gu N, Li J. PTPRO Promotes Oxidized Low-Density Lipoprotein Induced Oxidative Stress and Cell Apoptosis through Toll-Like Receptor 4/Nuclear Factor kappaB Pathway. Cell Physiol Biochem. 2017; 42:495-505.

<https://doi.org/10.1159/000477596> PMID:[28578349](https://pubmed.ncbi.nlm.nih.gov/28578349)

191. He ZQ, Liang C, Wang H, Wu ZG. Dysfunction of AQP7 in the periadventitial fat: A novel trigger of atherosclerosis. Med Hypotheses. 2008; 70:92-95.

<https://doi.org/10.1016/j.mehy.2007.04.022>

[PMID:17562358](https://www.ncbi.nlm.nih.gov/pubmed/17562358)

192. Farghaly HS, Metwalley KA, Raafat DM, Algowhary M, Said GM. Epicardial Fat Thickness in Children with Subclinical Hypothyroidism and Its Relationship to Subclinical Atherosclerosis: A Pilot Study. Horm Res Paediatr. 2019; 92:99-105.

<https://doi.org/10.1159/000503287>

[PMID:31618734](https://www.ncbi.nlm.nih.gov/pubmed/31618734)

193. Ishimaru K, Yoshioka K, Kano K, Kurano M, Saigusa D, Aoki J, Yatomi Y, Takuwa N, Okamoto Y, Proia RL, Takuwa Y. Sphingosine kinase-2 prevents macrophage cholesterol accumulation and atherosclerosis by stimulating autophagic lipid degradation. Sci Rep. 2019; 9:18329.

<https://doi.org/10.1038/s41598-019-54877-6>

[PMID:31797978](https://www.ncbi.nlm.nih.gov/pubmed/31797978)

194. Ellenbroek GH, van Puijvelde GH, Anas AA, Bot M, Asbach M, Schoneveld A, van Santbrink PJ, Foks AC, Timmers L, Doevendans PA, Pasterkamp G, Hoefer IE, van der Poll T, et al. Leukocyte TLR5 deficiency inhibits atherosclerosis by reduced macrophage recruitment and defective T-cell responsiveness. Sci Rep. 2017; 7:42688.

<https://doi.org/10.1038/srep42688>

[PMID:28202909](https://www.ncbi.nlm.nih.gov/pubmed/28202909)

195. Suarez-Martinez E, Husain K, Ferder L. Adiponectin expression and the cardioprotective role of the vitamin D receptor activator paricalcitol and the angiotensin converting enzyme inhibitor enalapril in ApoE-deficient mice. Ther Adv Cardiovasc Dis. 2014; 8:224-36.

<https://doi.org/10.1177/1753944714542593>

[PMID:25037058](https://www.ncbi.nlm.nih.gov/pubmed/25037058)

196. Wang J, Sun C, Gerdes N, Liu C, Liao M, Liu J, Shi MA, He A, Zhou Y, Sukhova GK, Chen H, Cheng XW, Kuzuya M, et al. Interleukin 18 function in atherosclerosis is mediated by the interleukin 18 receptor and the Na-Cl co-transporter. Nat Med. 2015; 21:820-26.

<https://doi.org/10.1038/nm.3890>

[PMID:26099046](https://www.ncbi.nlm.nih.gov/pubmed/26099046)

197. Tumurkhuu G, Dagvadorj J, Porritt RA, Crother TR, Shimada K, Tarling EJ, Erbay E, Arditi M, Chen S. Chlamydia pneumoniae Hijacks a Host Autoregulatory IL-1? Loop to Drive Foam Cell Formation and Accelerate Atherosclerosis. Cell Metab. 2018; 28:432-448.e4.

<https://doi.org/10.1016/j.cmet.2018.05.027>

[PMID:29937375](https://www.ncbi.nlm.nih.gov/pubmed/29937375)

198. Dong C, Beecham A, Wang L, Blanton SH, Rundek T, Sacco RL. Follow-up association study of linkage regions reveals multiple candidate genes for carotid plaque in Dominicans. Atherosclerosis. 2012; 223:177-83.

<https://doi.org/10.1016/j.atherosclerosis.2012.03.025>

[PMID:22503546](https://www.ncbi.nlm.nih.gov/pubmed/22503546)

199. Borborema ME, Crovella S, Oliveira D, de Azevedo Silva J. Inflammasome activation by NLRP1 and NLRC4 in patients with coronary stenosis. Immunobiology. 2020; 225:151940.

<https://doi.org/10.1016/j.imbio.2020.151940>

[PMID:32276737](https://www.ncbi.nlm.nih.gov/pubmed/32276737)

200. Mehta NU, Grijalva V, Hama S, Wagner A, Navab M, Fogelman AM, Reddy ST, Apolipoprotein E. Apolipoprotein E-/- Mice Lacking Hemopexin Develop Increased Atherosclerosis via Mechanisms That Include Oxidative Stress and Altered Macrophage Function. Arterioscler Thromb Vasc Biol. 2016; 36:1152-63.

<https://doi.org/10.1161/ATVBAHA.115.306991>

[PMID:27079878](https://www.ncbi.nlm.nih.gov/pubmed/27079878)

201. Wigren M, Bengtsson D, Duner P, Olofsson K, Bjorkbacka H, Bengtsson E, Fredrikson GN, Nilsson J. Atheroprotective effects of Alum are associated with capture of oxidized LDL antigens and activation of regulatory T cells. Circ Res. 2009; 104:e62-70.

<https://doi.org/10.1161/CIRCRESAHA.109.196667>

[PMID:19478203](https://www.ncbi.nlm.nih.gov/pubmed/19478203)

202. Chen Q, Xiang J, Gong R, Fang HY, Xu CC, Zhang HZ, Wu YQ. Atorvastatin downregulates HSP22 expression in an atherosclerotic model in vitro and in vivo. Int J Mol Med. 2019; 43:821-29.

<https://doi.org/10.3892/ijmm.2018.4015>

[PMID:30535427](https://www.ncbi.nlm.nih.gov/pubmed/30535427)

203. Zhang M, Qu X, Yuan F, Yang Y, Xu L, Dai J, Wang W, Fei J, Hou X, Fang W. Ghrelin receptor deficiency aggravates atherosclerotic plaque instability. Front Biosci. 2015; 20:604-13.

<https://doi.org/10.2741/4325>

[PMID:25553467](https://www.ncbi.nlm.nih.gov/pubmed/25553467)

204. Sakamoto A, Sugamoto Y, Tokunaga Y, Yoshimuta T, Hayashi K, Konno T, Kawashiri MA, Takeda Y, Yamagishi M. Expression profiling of the ephrin (EFN) and Eph receptor (EPH) family of genes in atherosclerosis-related human cells. J Int Med Res. 2011; 39:522-27.

<https://doi.org/10.1177/147323001103900220>

[PMID:21672356](https://www.ncbi.nlm.nih.gov/pubmed/21672356)

205. Han X, Kitamoto S, Lian Q, Boisvert WA. Interleukin-10 facilitates both cholesterol uptake and efflux in macrophages. J Biol Chem. 2009; 284:32950-58.

<https://doi.org/10.1074/jbc.M109.040899>

[PMID:19776020](https://www.ncbi.nlm.nih.gov/pubmed/19776020)

206. Golden D, Kolmakova A, Sura S, Vella AT, Manichaikul A, Wang XQ, Bielinski SJ, Taylor KD, Chen YI, Rich SS, Rodriguez A. Lymphocyte activation gene 3 and coronary artery disease. JCI Insight. 2016; 1:e88628.

<https://doi.org/10.1172/jci.insight.88628>

[PMID:27777974](https://www.ncbi.nlm.nih.gov/pubmed/27777974)

207. Borrell-Pages M, Carolina Romero J, Badimon L. LRP5 and plasma cholesterol levels modulate the canonical Wnt pathway in peripheral blood leukocytes. Immunol Cell Biol. 2015; 93:653-61.

<https://doi.org/10.1038/icb.2015.41>

[PMID:25748163](https://www.ncbi.nlm.nih.gov/pubmed/25748163)

208. Wang L, Zheng Z, Feng X, Zang X, Ding W, Wu F, Zhao Q. circRNA/lncRNA-miRNA-mRNA Network in Oxidized, Low-Density, Lipoprotein-Induced Foam Cells. DNA Cell Biol. 2019; 38:1499-511.

<https://doi.org/10.1089/dna.2019.4865>

[PMID:31804889](https://www.ncbi.nlm.nih.gov/pubmed/31804889)

209. Lacey M, Baribault C, Ehrlich KC, Ehrlich M. Data showing atherosclerosis-associated differentially methylated regions are often at enhancers. Data Brief. 2019; 23:103812.

<https://doi.org/10.1016/j.dib.2019.103812>

[PMID:31372457](https://www.ncbi.nlm.nih.gov/pubmed/31372457)

210. Malhotra R, Mauer AC, Lino Cardenas CL, Guo X, Yao J, Zhang X, Wunderer F, Smith AV, Wong Q, Pechlivanis S, Hwang SJ, Wang J, Lu L, et al. HDAC9 is implicated in atherosclerotic aortic calcification and affects vascular smooth muscle cell phenotype. Nat Genet. 2019; 51:1580-87.

<https://doi.org/10.1038/s41588-019-0514-8>

[PMID:31659325](https://www.ncbi.nlm.nih.gov/pubmed/31659325)

211. Xu S, Yin M, Koroleva M, Mastrangelo MA, Zhang W, Bai P, Little PJ, Jin ZG. SIRT6 protects against endothelial dysfunction and atherosclerosis in mice. Aging (Albany NY). 2016; 8:1064-82.

<https://doi.org/10.18632/aging.100975>

[PMID:27249230](https://www.ncbi.nlm.nih.gov/pubmed/27249230)

212. Yamamoto K, Tajima Y, Hasegawa A, Takahashi Y, Kojima M, Watanabe R, Sato K, Shichiri M, Watanabe T. Contrasting effects of stanniocalcin-related polypeptides on macrophage foam cell formation and vascular smooth muscle cell migration. Peptides. 2016; 82:120-27.

<https://doi.org/10.1016/j.peptides.2016.06.009>

[PMID:27346255](https://www.ncbi.nlm.nih.gov/pubmed/27346255)

213. Lichtenstein L, Berbee JF, van Dijk SJ, van Dijk KW, Bensadoun A, Kema IP, Voshol PJ, Muller M, Rensen PC, Kersten S. Angptl4 upregulates cholesterol synthesis in liver via inhibition of LPL- and HL-dependent hepatic cholesterol uptake. Arterioscler Thromb Vasc Biol. 2007; 27:2420-27.

<https://doi.org/10.1161/ATVBAHA.107.151894>

[PMID:17761937](https://www.ncbi.nlm.nih.gov/pubmed/17761937)

214. Dulin E, Garcia-Barreno P, Guisasola MC. Genetic variations of HSPA1A, the heat shock protein levels, and risk of atherosclerosis. Cell Stress Chaperones. 2012; 17:507-16.

<https://doi.org/10.1007/s12192-012-0328-4>

[PMID:22328194](https://www.ncbi.nlm.nih.gov/pubmed/22328194)

215. Grainger AT, Jones MB, Li J, Chen MH, Manichaikul A, Shi W. Genetic analysis of atherosclerosis identifies a major susceptibility locus in the major histocompatibility complex of mice. Atherosclerosis. 2016; 254:124-32.

<https://doi.org/10.1016/j.atherosclerosis.2016.10.011>

[PMID:27736672](https://www.ncbi.nlm.nih.gov/pubmed/27736672)

216. Zeboudj L, Maitre M, Guyonnet L, Laurans L, Joffre J, Lemarie J, Bourcier S, Nour-Eldine W, Guerin C, Friard J, Wakkach A, Fabre E, Tedgui A, et al. Selective EGF-Receptor Inhibition in CD4^+^ T Cells Induces Anergy and Limits Atherosclerosis. J Am Coll Cardiol. 2018; 71:160-72.

<https://doi.org/10.1016/j.jacc.2017.10.084>

[PMID:29325640](https://www.ncbi.nlm.nih.gov/pubmed/29325640)

217. Subramanian M, Thorp E, Tabas I. Identification of a non-growth factor role for GM-CSF in advanced atherosclerosis: promotion of macrophage apoptosis and plaque necrosis through IL-23 signaling. Circ Res. 2015; 116:e13-24.

<https://doi.org/10.1161/CIRCRESAHA.116.304794>

[PMID:25348165](https://www.ncbi.nlm.nih.gov/pubmed/25348165)

218. Ait-Oufella H, Herbin O, Lahoute C, Coatrieux C, Loyer X, Joffre J, Laurans L, Ramkhelawon B, Blanc-Brude O, Karabina S, Girard CA, Payre C, Yamamoto K, et al. Group X secreted phospholipase A2 limits the development of atherosclerosis in LDL receptor-null mice. Arterioscler Thromb Vasc Biol. 2013; 33:466-73.

<https://doi.org/10.1161/ATVBAHA.112.300309>

[PMID:23349189](https://www.ncbi.nlm.nih.gov/pubmed/23349189)

219. Grandoch M, Hoffmann J, Rock K, Wenzel F, Oberhuber A, Schelzig H, Fischer JW. Novel effects of adenosine receptors on pericellular hyaluronan matrix: implications for human smooth muscle cell phenotype and interactions with monocytes during atherosclerosis. Basic Res Cardiol. 2013; 108:340.

<https://doi.org/10.1007/s00395-013-0340-6>

[PMID:23440385](https://www.ncbi.nlm.nih.gov/pubmed/23440385)

220. Ramkhelawon B, Yang Y, van Gils JM, Hewing B, Rayner KJ, Parathath S, Guo L, Oldebeken S, Feig JL, Fisher EA, Moore KJ. Hypoxia induces netrin-1 and Unc5b in atherosclerotic plaques: mechanism for macrophage retention and survival. Arterioscler Thromb Vasc Biol. 2013; 33:1180-88.

<https://doi.org/10.1161/ATVBAHA.112.301008>

[PMID:23599441](https://www.ncbi.nlm.nih.gov/pubmed/23599441)

221. Abd Alla J, Langer A, Elzahwy SS, Arman-Kalcek G, Streichert T, Quitterer U. Angiotensin-converting enzyme inhibition down-regulates the pro-atherogenic chemokine receptor 9 (CCR9)-chemokine ligand 25 (CCL25) axis. J Biol Chem. 2010; 285:23496-505.

<https://doi.org/10.1074/jbc.M110.117481>

[PMID:20504763](https://www.ncbi.nlm.nih.gov/pubmed/20504763)

222. Ihling C, Szombathy T, Bohrmann B, Brockhaus M, Schaefer HE, Loeffler BM. Coexpression of endothelin-converting enzyme-1 and endothelin-1 in different stages of human atherosclerosis. Circulation. 2001; 104:864-69.

<https://doi.org/10.1161/hc3301.094742>

[PMID:11514370](https://www.ncbi.nlm.nih.gov/pubmed/11514370)

223. Tian FJ, An LN, Wang GK, Zhu JQ, Li Q, Zhang YY, Zeng A, Zou J, Zhu RF, Han XS, Shen N, Yang HT, Zhao XX, et al. Elevated microRNA-155 promotes foam cell formation by targeting HBP1 in atherogenesis. Cardiovasc Res. 2014; 103:100-10.

<https://doi.org/10.1093/cvr/cvu070>

[PMID:24675724](https://www.ncbi.nlm.nih.gov/pubmed/24675724)

224. Ni CW, Qiu H, Rezvan A, Kwon K, Nam D, Son DJ, Visvader JE, Jo H. Discovery of novel mechanosensitive genes in vivo using mouse carotid artery endothelium exposed to disturbed flow. Blood. 2010; 116:e66-73.

<https://doi.org/10.1182/blood-2010-04-278192>

[PMID:20551377](https://www.ncbi.nlm.nih.gov/pubmed/20551377)

225. Lee WH, Kim SH, Lee Y, Lee BB, Kwon B, Song H, Kwon BS, Park JE. Tumor necrosis factor receptor superfamily 14 is involved in atherogenesis by inducing proinflammatory cytokines and matrix metalloproteinases. Arterioscler Thromb Vasc Biol. 2001; 21:2004-10.

<https://doi.org/10.1161/hq1201.098945>

[PMID:11742877](https://www.ncbi.nlm.nih.gov/pubmed/11742877)

226. Shen J, Shang Q, Wong CK, Li EK, Wang S, Li RJ, Lee KL, Leung YY, Ying KY, Yim CW, Kun EW, Leung MH, Li M, et al. IL-33 and soluble ST2 levels as novel predictors for remission and progression of carotid plaque in early rheumatoid arthritis: A prospective study. Semin Arthritis Rheum. 2015; 45:18-27.

<https://doi.org/10.1016/j.semarthrit.2015.02.001>

[PMID:25798875](https://www.ncbi.nlm.nih.gov/pubmed/25798875)

227. Haley KJ, Lilly CM, Yang JH, Feng Y, Kennedy SP, Turi TG, Thompson JF, Sukhova GH, Libby P, Lee RT. Overexpression of eotaxin and the CCR3 receptor in human atherosclerosis: using genomic technology to identify a potential novel pathway of vascular inflammation. Circulation. 2000; 102:2185-89.

<https://doi.org/10.1161/01.CIR.102.18.2185>

[PMID:11056090](https://www.ncbi.nlm.nih.gov/pubmed/11056090)

228. Martin-Lorenzo M, Zubiri I, Maroto AS, Gonzalez-Calero L, Posada-Ayala M, de la Cuesta F, Mourino-Alvarez L, Lopez-Almodovar LF, Calvo-Bonacho E, Ruilope LM, Padial LR, Barderas MG, Vivanco F, et al. KLK1 and ZG16B proteins and arginine-proline metabolism identified as novel targets to monitor atherosclerosis, acute coronary syndrome and recovery. Metabolomics. 2015; 11:1056-1067. <https://doi.org/10.1007/s11306-014-0761-8> PMID:[26413039](https://pubmed.ncbi.nlm.nih.gov/26413039)

229. Medina I, Cougoule C, Drechsler M, Bermudez B, Koenen RR, Sluimer J, Wolfs I, Doring Y, Herias V, Gijbels M, Bot I, de Jager S, Weber C, et al. Hck/Fgr Kinase Deficiency Reduces Plaque Growth and Stability by Blunting Monocyte Recruitment and Intraplaque Motility. Circulation. 2015; 132:490-501.

<https://doi.org/10.1161/CIRCULATIONAHA.114.012316>

[PMID:26068045](https://www.ncbi.nlm.nih.gov/pubmed/26068045)

230. Malhotra R, Wunderer F, Barnes HJ, Bagchi A, Buswell MD, O'Rourke CD, Slocum CL, Ledsky CD, Peneyra KM, Sigurslid H, Corman B, Johansson KB, Rhee DK, et al. Hepcidin Deficiency Protects Against Atherosclerosis. Arterioscler Thromb Vasc Biol. 2019; 39:178-87.

<https://doi.org/10.1161/ATVBAHA.118.312215>

[PMID:30587002](https://www.ncbi.nlm.nih.gov/pubmed/30587002)

231. Li R, Paul A, Ko KW, Sheldon M, Rich BE, Terashima T, Dieker C, Cormier S, Li L, Nour EA, Chan L, Oka K. Interleukin-7 induces recruitment of monocytes/macrophages to endothelium. Eur Heart J. 2012; 33:3114-23.

<https://doi.org/10.1093/eurheartj/ehr245>

[PMID:21804111](https://www.ncbi.nlm.nih.gov/pubmed/21804111)

232. Tran-Lundmark K, Tran PK, Paulsson-Berne G, Friden V, Soininen R, Tryggvason K, Wight TN, Kinsella MG, Boren J, Hedin U. Heparan sulfate in perlecan promotes mouse atherosclerosis: roles in lipid permeability, lipid retention, and smooth muscle cell proliferation. Circ Res. 2008; 103:43-52.

<https://doi.org/10.1161/CIRCRESAHA.107.172833>

[PMID:18596265](https://www.ncbi.nlm.nih.gov/pubmed/18596265)

233. Liberale L, Bertolotto M, Carbone F, Contini P, Wust P, Spinella G, Pane B, Palombo D, Bonaventura A, Pende A, Mach F, Dallegri F, Camici GG, Montecucco F. Resistin exerts a beneficial role in atherosclerotic plaque inflammation by inhibiting neutrophil migration. Int J Cardiol. 2018; 272:13-19.

<https://doi.org/10.1016/j.ijcard.2018.07.112>

[PMID:30075966](https://www.ncbi.nlm.nih.gov/pubmed/30075966)

234. Liu J, Guo K, Hu L, Luo T, Ma Y, Zhang Y, Lai W, Guo Z. ZAP70 deficiency promotes reverse cholesterol transport through MAPK/ERK pathway in Jurkat cell. Mol Immunol. 2019; 107:21-28.

<https://doi.org/10.1016/j.molimm.2019.01.001>

[PMID:30639475](https://www.ncbi.nlm.nih.gov/pubmed/30639475)

235. Bamias G, Stamatelopoulos K, Zampeli E, Protogerou A, Sigala F, Papamichael C, Christopoulos P, Kitas GD, Sfikakis PP. Circulating levels of TNF-like cytokine 1A correlate with the progression of atheromatous lesions in patients with rheumatoid arthritis. Clin Immunol. 2013; 147:144-50.

<https://doi.org/10.1016/j.clim.2013.03.002>

[PMID:23598291](https://www.ncbi.nlm.nih.gov/pubmed/23598291)

236. Sanjurjo L, Aran G, Tellez E, Amezaga N, Armengol C, Lopez D, Prats C, Sarrias MR. CD5L Promotes M2 Macrophage Polarization through Autophagy-Mediated Upregulation of ID3. Front Immunol. 2018; 9:480.

<https://doi.org/10.3389/fimmu.2018.00480>

[PMID:29593730](https://www.ncbi.nlm.nih.gov/pubmed/29593730)

237. Goo YH, Son SH, Yechoor VK, Paul A. Transcriptional Profiling of Foam Cells Reveals Induction of Guanylate-Binding Proteins Following Western Diet Acceleration of Atherosclerosis in the Absence of Global Changes in Inflammation. J Am Heart Assoc. 2016; 5:e002663.

<https://doi.org/10.1161/JAHA.115.002663>

[PMID:27091181](https://www.ncbi.nlm.nih.gov/pubmed/27091181)

238. Lu YJ, Jan YJ, Ko BS, Liang SM, Chen L, Wu CC, Chin CH, Kuo CC, Yet SF, Liou JY. Expression of Nik-related kinase in smooth muscle cells attenuates vascular inflammation and intimal hyperplasia. Aging (Albany NY). 2020; 12:7511-33.

<https://doi.org/10.18632/aging.103104>

[PMID:32330120](https://www.ncbi.nlm.nih.gov/pubmed/32330120)

239. Gargalovic PS, Gharavi NM, Clark MJ, Pagnon J, Yang WP, He A, Truong A, Baruch-Oren T, Berliner JA, Kirchgessner TG, Lusis AJ. The unfolded protein response is an important regulator of inflammatory genes in endothelial cells. Arterioscler Thromb Vasc Biol. 2006; 26:2490-96.

<https://doi.org/10.1161/01.ATV.0000242903.41158.a1>

[PMID:16931790](https://www.ncbi.nlm.nih.gov/pubmed/16931790)

240. Adachi H, Kondo T, Koh GY, Nagy A, Oike Y, Araki E. Angptl4 deficiency decreases serum triglyceride levels in low-density lipoprotein receptor knockout mice and streptozotocin-induced diabetic mice. Biochem Biophys Res Commun. 2011; 409:177-80.

<https://doi.org/10.1016/j.bbrc.2011.04.110>

[PMID:21549101](https://www.ncbi.nlm.nih.gov/pubmed/21549101)

241. Polfus LM, Smith JA, Shimmin LC, Bielak LF, Morrison AC, Kardia SL, Peyser PA, Hixson JE. Genome-wide association study of gene by smoking interactions in coronary artery calcification. PLoS One. 2013; 8:e74642.

<https://doi.org/10.1371/journal.pone.0074642>

[PMID:24098343](https://www.ncbi.nlm.nih.gov/pubmed/24098343)

242. Mallat Z, Corbaz A, Scoazec A, Besnard S, Leseche G, Chvatchko Y, Tedgui A. Expression of interleukin-18 in human atherosclerotic plaques and relation to plaque instability. Circulation. 2001; 104:1598-603.

<https://doi.org/10.1161/hc3901.096721>

[PMID:11581135](https://www.ncbi.nlm.nih.gov/pubmed/11581135)

243. Dong J, Song C, Zhang L, Feng X, Feng R, Lu Q, Zhao Z, Bao J, Zhou J, Jing Z. Identified key genes related to carotid atheroma plaque from gene expression chip. Artif Cells Nanomed Biotechnol. 2017; 45:1-6.

<https://doi.org/10.1080/21691401.2016.1216858>

[PMID:27684644](https://www.ncbi.nlm.nih.gov/pubmed/27684644)

244. Zhao C, Ikeda S, Arai T, Naka-Mieno M, Sato N, Muramatsu M, Sawabe M. Association of the RYR3 gene polymorphisms with atherosclerosis in elderly Japanese population. BMC Cardiovasc Disord. 2014; 14:6.

<https://doi.org/10.1186/1471-2261-14-6>

[PMID:24423397](https://www.ncbi.nlm.nih.gov/pubmed/24423397)

245. Krivospitskaya O, Elmabsout AA, Sundman E, Soderstrom LA, Ovchinnikova O, Gidlof AC, Scherbak N, Norata GD, Samnegard A, Torma H, Abdel-Halim SM, Jansson JH, Eriksson P, et al. A CYP26B1 polymorphism enhances retinoic acid catabolism and may aggravate atherosclerosis. Mol Med. 2012; 18:712-18.

<https://doi.org/10.2119/molmed.2012.00094>

[PMID:22415012](https://www.ncbi.nlm.nih.gov/pubmed/22415012)

246. Ruan ZB, Fu XL, Li W, Ye J, Wang RZ, Zhu L. Effect of notch1,2,3 genes silicing on NF-kappaB signaling pathway of macrophages in patients with atherosclerosis. Biomed Pharmacother. 2016; 84:666-673. https://doi.org: 10.1016/j.biopha.2016.09.078 PMID:27697639

247. Kumar P, Raghavan S, Shanmugam G, Shanmugam N. Ligation of RAGE with ligand S100B attenuates ABCA1 expression in monocytes. Metabolism. 2013; 62:1149-58.

<https://doi.org/10.1016/j.metabol.2013.02.006>

[PMID:23523156](https://www.ncbi.nlm.nih.gov/pubmed/23523156)

248. Yoon S, Kuivaniemi H, Gatalica Z, Olson JM, Buttice G, Ye S, Norris BA, Malcom GT, Strong JP, Tromp G. MMP13 promoter polymorphism is associated with atherosclerosis in the abdominal aorta of young black males. Matrix biology : journal of the International Society for Matrix Biol. 2002;21:487-498.

<https://doi.org/10.1016/S0945-053X(02)00053-7> PMID:[12392760](https://pubmed.ncbi.nlm.nih.gov/12392760)

249. Liao J, Xie Y, Lin Q, Yang X, An X, Xia Y, Du J, Wang F, Li HH. Immunoproteasome subunit ?5i regulates diet-induced atherosclerosis through altering MERTK-mediated efferocytosis in Apoe knockout mice. J Pathol. 2020; 250:275-87.

<https://doi.org/10.1002/path.5368>

[PMID:31758542](https://www.ncbi.nlm.nih.gov/pubmed/31758542)

250. Wagsater D, Bjork H, Zhu C, Bjorkegren J, Valen G, Hamsten A, Eriksson P. ADAMTS-4 and -8 are inflammatory regulated enzymes expressed in macrophage-rich areas of human atherosclerotic plaques. Atherosclerosis. 2008; 196:514-22.

<https://doi.org/10.1016/j.atherosclerosis.2007.05.018>

[PMID:17606262](https://www.ncbi.nlm.nih.gov/pubmed/17606262)

251. Guo J, Liang W, Li J, Long J. Knockdown of FSTL1 inhibits oxLDL-induced inflammation responses through the TLR4/MyD88/NF-?B and MAPK pathway. Biochem Biophys Res Commun. 2016; 478:1528-33.

<https://doi.org/10.1016/j.bbrc.2016.08.138>

[PMID:27569284](https://www.ncbi.nlm.nih.gov/pubmed/27569284)

252. Manthey HD, Cochain C, Barnsteiner S, Karshovska E, Pelisek J, Koch M, Chaudhari SM, Busch M, Eckstein HH, Weber C, Koenen RR, Zernecke A. CCR6 selectively promotes monocyte mediated inflammation and atherogenesis in mice. Thromb Haemost. 2013; 110:1267-77.

<https://doi.org/10.1160/TH13-01-0017>

[PMID:24114205](https://www.ncbi.nlm.nih.gov/pubmed/24114205)

253. Wan W, Lim JK, Lionakis MS, Rivollier A, McDermott DH, Kelsall BL, Farber JM, Murphy PM. Genetic deletion of chemokine receptor Ccr6 decreases atherogenesis in ApoE-deficient mice. Circ Res. 2011; 109:374-81.

<https://doi.org/10.1161/CIRCRESAHA.111.242578>

[PMID:21680896](https://www.ncbi.nlm.nih.gov/pubmed/21680896)

254. Liu L, Zeng P, Yang X, Duan Y, Zhang W, Ma C, Zhang X, Yang S, Li X, Yang J, Liang Y, Han H, Zhu Y, et al. Inhibition of Vascular Calcification. Arterioscler Thromb Vasc Biol. 2018; 38:2382-95.

<https://doi.org/10.1161/ATVBAHA.118.311546>

[PMID:30354214](https://www.ncbi.nlm.nih.gov/pubmed/30354214)

255. Salic K, Morrison MC, Verschuren L, Wielinga PY, Wu L, Kleemann R, Gjorstrup P, Kooistra T. Resolvin E1 attenuates atherosclerosis in absence of cholesterol-lowering effects and on top of atorvastatin. Atherosclerosis. 2016; 250:158-65.

<https://doi.org/10.1016/j.atherosclerosis.2016.05.001>

[PMID:27236706](https://www.ncbi.nlm.nih.gov/pubmed/27236706)

256. Molusky MM, Hsieh J, Lee SX, Ramakrishnan R, Tascau L, Haeusler RA, Accili D, Tall AR. Metformin and AMP Kinase Activation Increase Expression of the Sterol Transporters ABCG5/8 (ATP-Binding Cassette Transporter G5/G8) With Potential Antiatherogenic Consequences. Arterioscler Thromb Vasc Biol. 2018; 38:1493-503.

<https://doi.org/10.1161/ATVBAHA.118.311212>

[PMID:29853564](https://www.ncbi.nlm.nih.gov/pubmed/29853564)

257. Tsaousi A, Williams H, Lyon CA, Taylor V, Swain A, Johnson JL, George SJ. Wnt4/?-catenin signaling induces VSMC proliferation and is associated with intimal thickening. Circ Res. 2011; 108:427-36.

<https://doi.org/10.1161/CIRCRESAHA.110.233999>

[PMID:21193738](https://www.ncbi.nlm.nih.gov/pubmed/21193738)

258. Rajamoorthi A, Lee RG, Baldan A. Therapeutic silencing of FSP27 reduces the progression of atherosclerosis in Ldlr^-/-^ mice. Atherosclerosis. 2018; 275:43-49.

<https://doi.org/10.1016/j.atherosclerosis.2018.05.045>

[PMID:29859472](https://www.ncbi.nlm.nih.gov/pubmed/29859472)

259. Turner AW, Nikpay M, Silva A, Lau P, Martinuk A, Linseman TA, Soubeyrand S, McPherson R. Functional interaction between COL4A1/COL4A2 and SMAD3 risk loci for coronary artery disease. Atherosclerosis. 2015; 242:543-52.

<https://doi.org/10.1016/j.atherosclerosis.2015.08.008>

[PMID:26310581](https://www.ncbi.nlm.nih.gov/pubmed/26310581)

260. Tousoulis D, Briasoulis A, Vogiatzi G, Valatsou A, Kourkouti P, Pantopoulou A, Papageorgiou N, Perrea D, Stefanadis C. Infusion of lin-/sca-1+ and endothelial progenitor cells improves proinflammatory and oxidative stress markers in atherosclerotic mice. Int J Cardiol. 2013; 167:1900-05.

<https://doi.org/10.1016/j.ijcard.2012.04.148>

[PMID:22622053](https://www.ncbi.nlm.nih.gov/pubmed/22622053)

261. Kim JB, Zhao Q, Nguyen T, Pjanic M, Cheng P, Wirka R, Travisano S, Nagao M, Kundu R, Quertermous T. Environment-Sensing Aryl Hydrocarbon Receptor Inhibits the Chondrogenic Fate of Modulated Smooth Muscle Cells in Atherosclerotic Lesions. Circulation. 2020; 142:575-90.

<https://doi.org/10.1161/CIRCULATIONAHA.120.045981>

[PMID:32441123](https://www.ncbi.nlm.nih.gov/pubmed/32441123)

262. Yang C, Lu M, Chen W, He Z, Hou X, Feng M, Zhang H, Bo T, Zhou X, Yu Y, Zhang H, Zhao M, Wang L, et al. Thyrotropin aggravates atherosclerosis by promoting macrophage inflammation in plaques. J Exp Med. 2019; 216:1182-98.

<https://doi.org/10.1084/jem.20181473>

[PMID:30940720](https://www.ncbi.nlm.nih.gov/pubmed/30940720)

263. Kong X, Fang M, Li P, Fang F, Xu Y. HDAC2 deacetylates class II transactivator and suppresses its activity in macrophages and smooth muscle cells. J Mol Cell Cardiol. 2009; 46:292-99.

<https://doi.org/10.1016/j.yjmcc.2008.10.023>

[PMID:19041327](https://www.ncbi.nlm.nih.gov/pubmed/19041327)

264. Wu H, Liu T, Hou H. Knockdown of LINC00657 inhibits ox-LDL-induced endothelial cell injury by regulating miR-30c-5p/Wnt7b/?-catenin. Mol Cell Biochem. 2020; 472:145-55.

<https://doi.org/10.1007/s11010-020-03793-9>

[PMID:32577947](https://www.ncbi.nlm.nih.gov/pubmed/32577947)

265. Wang H, Liu D, Zhang H. Investigation of the Underlying Genes and Mechanism of Macrophage-Enriched Ruptured Atherosclerotic Plaques Using Bioinformatics Method. J Atheroscler Thromb. 2019; 26:636-58.

<https://doi.org/10.5551/jat.45963>

[PMID:30643084](https://www.ncbi.nlm.nih.gov/pubmed/30643084)

266. Zhang L, Wang F, Wang J, Wang Y, Fang Y. Intestinal fatty acid-binding protein mediates atherosclerotic progress through increasing intestinal inflammation and permeability. J Cell Mol Med. 2020; 24:5205-12.

<https://doi.org/10.1111/jcmm.15173>

[PMID:32220004](https://www.ncbi.nlm.nih.gov/pubmed/32220004)

267. Zhou Z, Subramanian P, Sevilmis G, Globke B, Soehnlein O, Karshovska E, Megens R, Heyll K, Chun J, Saulnier-Blache JS, Reinholz M, van Zandvoort M, Weber C, Schober A. Lipoprotein-derived lysophosphatidic acid promotes atherosclerosis by releasing CXCL1 from the endothelium. Cell Metab. 2011; 13:592-600.

<https://doi.org/10.1016/j.cmet.2011.02.016>

[PMID:21531341](https://www.ncbi.nlm.nih.gov/pubmed/21531341)

268. Yoshino Y, Kohara K, Abe M, Ochi S, Mori Y, Yamashita K, Igase M, Tabara Y, Mori T, Miki T, Ueno S. Missense variants of the alanine: glyoxylate aminotransferase 2 gene correlated with carotid atherosclerosis in the Japanese population. J Biol Regul Homeost Agents. 2014; 28:605-14.

[PMID:25620171](https://www.ncbi.nlm.nih.gov/pubmed/25620171)

269. Lee SM, Nguyen D, Hu Z, Abbott GW. Kcne2 deletion promotes atherosclerosis and diet-dependent sudden death. J Mol Cell Cardiol. 2015; 87:148-51.

<https://doi.org/10.1016/j.yjmcc.2015.08.013>

[PMID:26307149](https://www.ncbi.nlm.nih.gov/pubmed/26307149)
